# Dynamics of TERT regulation via alternative splicing in stem cells and cancer cells

**DOI:** 10.1371/journal.pone.0289327

**Published:** 2023-08-02

**Authors:** Jeongjin J. Kim, Mohammed E. Sayed, Alexander Ahn, Aaron L. Slusher, Jeffrey Y. Ying, Andrew T. Ludlow

**Affiliations:** School of Kinesiology, University of Michigan, Ann Arbor, Michigan, United States of America; TotiCell Limited, Bangladesh, BANGLADESH

## Abstract

Part of the regulation of telomerase activity includes the alternative splicing (AS) of the catalytic subunit telomerase reverse transcriptase (*TERT*). Although a therapeutic window for telomerase/TERT inhibition exists between cancer cells and somatic cells, stem cells express TERT and rely on telomerase activity for physiological replacement of cells. Therefore, identifying differences in *TERT* regulation between stem cells and cancer cells is essential for developing telomerase inhibition-based cancer therapies that reduce damage to stem cells. In this study, we measured *TERT* splice variant expression and telomerase activity in induced pluripotent stem cells (iPSCs), neural progenitor cells (NPCs), and non-small cell lung cancer cells (NSCLC, Calu-6 cells). We observed that a NOVA1-PTBP1-PTBP2 axis regulates *TERT* alternative splicing (AS) in iPSCs and their differentiation into NPCs. We also found that splice-switching of *TERT*, which regulates telomerase activity, is induced by different cell densities in stem cells but not cancer cells. Lastly, we identified cell type-specific splicing factors that regulate *TERT* AS. Overall, our findings represent an important step forward in understanding the regulation of *TERT* AS in stem cells and cancer cells.

## Introduction

Telomeres are a repeated DNA sequence (5’-TTAGGGn-3’) at the ends of linear chromosomes [1]. During DNA replication the lagging strand of telomeric DNA is not fully copied resulting in telomere shortening with each cell division [2]. After many cell divisions, a critically shortened telomere length is reached by a subset of telomeres resulting in a DNA damage response at chromosome ends and cell cycle withdraw (i.e., the induction of cellular senescence). This barrier to replication is important in somatic cells because it acts as a potent tumor suppressive mechanism preventing continued cell growth of damaged or mutant cells [3]. However, in stem cells, improper telomere maintenance results in aging-related phenotypes and telomeropathies [4]. In certain cell types, an enzyme, telomerase, can maintain or even elongate the shortest telomeres resulting in continued cell divisions [5]. Stem cells possess telomerase activity to maintain chromosome ends so that normal physiological cell replacement can occur [6]. In a sense this is regulated telomerase activity. However, cancer cells must overcome telomere shortening to keep proliferating and 85% of cancer cells rely on the reactivation of telomerase to maintain telomere length [7]. We refer to this type of telomerase as dysregulated telomerase activity. Additionally, most somatic cells lack detectable telomerase activity [8]. The expression pattern of telomerase activity, off in most somatic cells but on in most cancer cells, has led to telomerase inhibition being sought after as a potential cancer therapeutic option [9]. While some success on this front has been made, additional biochemical information about telomerase regulation will lead to better and more efficacious therapeutic options.

Telomerase is a ribonucleoprotein enzyme minimally consisting of two parts: telomerase reverse transcriptase (TERT) and telomerase RNA component (TERC or TR) [10]. Telomerase activity is regulated by transcriptional and post-transcriptional regulation [11–16]. One aspect of post-transcriptional regulation is alternative splicing (AS) of *TERT*. AS is a post-transcriptional mechanism that enhances proteome diversity by generating multiple isoforms of the same transcriptional unit or pre-mRNA [17, 18]. *TERT* is a gene that consists of 16 exons with 15 introns, in which only the full-length (FL) *TERT* can be translated to form active telomerase with all 16 exons [19]. Regulation of telomerase by AS has been reported in various contexts. For instance, during fetal kidney development, both FL *TERT* (exons 7/8 including *TERT*) and minus beta *TERT* (exons 7/8 excluding *TERT*) are co-expressed in early stages, but FL *TERT* is not expressed in later stages leading to loss of telomerase activity [20]. Minus beta *TERT*, despite there being a frame shift and a premature termination codon in frame in exon 10, has also been reported to form a protein and to have dominant-negative effects in certain cancer cells [14]. Additionally, minus beta TERT has also been observed to protect breast cancer cells from chemotherapeutic insults [21]. In embryonic stem cell differentiation, exon 2 is spliced out (Del2 *TERT*) when cells are differentiated to fibroblasts resulting in loss of FL TERT and telomerase activity [15]. In addition to the role that AS plays in turning off telomerase activity-coding FL *TERT*, isoforms generated by AS have different functions. Minus alpha (exon 6 partial exclusion) TERT is a dominant-negative isoform by sequestering TERC from FL TERT [22]. It has been proposed that minus gamma (exon 11 exclusion) TERT has dominant-negative effects on telomerase activity [23]. INS3 (159 bp insertion of intron 14 at the end of exon 14) and INS4 (600 bp insertion of the entire intron 14) are also dominant-negative isoforms of telomerase [24]. Delta 4–13 (exons 4–13 exclusion) was shown to stimulate cell proliferation seemingly by interacting with WNT/beta-catenin pathway [13]. Moreover, intron 11 retention appeared to be the driving force for nuclear detention of unspliced *TERT* mRNAs and to regulate telomerase activity to only dividing cells [25].

Measurement of *TERT* splice variants by RT-PCR is a challenge that must be addressed. Short fragment PCR detects exon splicing events quantitatively, but the detection and quantification of full-length intact mRNA splice variants (5’ mRNA cap to poly A tail) remains difficult to do. Further, most PCR methods that detect *TERT*, capture multiple splice variants rather than a single splice variant. For instance, the assay we use to quantify exons 7/8 including *TERT* captures more than just FL *TERT*. This assay also detects other *TERT* splice variants such as intron 11 retention, minus and plus gamma, minus alpha, INS3 and INS4. However, it should be noted that the exons 7/8 assay is moderately correlated to telomerase enzyme activity [16]. The best estimates of the number of molecules per cell of telomerase coding *TERT* mRNA indicate that FL *TERT* is a minor splice variant and the majority of the transcripts are alternatively spliced to either degraded, dominant-negative, or splice variants with unknown functions [26]. Despite the difficulties of measuring *TERT* splice variants, we can very carefully quantify specific exon events (exons 7/8, exons 6/9, etc.) and we call the exons 7/8 including *TERT*, “potential full-length *TERT*” due to the correlation with telomerase enzyme activity measures. We also measure minus beta *TERT* using an assay that detects the novel exon junction made between exons 6 and 9, which we refer to as exons 6/9 minus beta or exons 7/8 exclusion. While our assays only detect single exon events, they do not rule out the significance of full-length mRNAs with multiple combinations of these events together. Using these assays, we explore the regulatory mechanisms of *TERT* AS (i.e., inclusion of exons 7/8 or exclusion of exons 7/8) in multiple contexts (i.e., differentiation to specific cell types, specific tumor types and under different growth stresses) as they remain elusive.

In this study, we set out to identify regulated and dysregulated *TERT* splice variant expression to identify a potential cancer therapeutic window. We first determined if the *TERT* AS was regulated by a NOVA1-PTBP1-PTBP2 axis, during stem cell differentiation into neural progenitor cells, and indeed observed it was. We previously identified in cancer cells that *TERT* AS was regulated by a NOVA1-PTBP1-PTBP2 axis [16, 27]. Next, we made a serendipitous observation that stem cell density impacted *TERT* splice variant expression but that cancer cells did not seem to utilize this mechanism. Finally, based on public database analysis, correlational analysis, and experimental observations, we identified splicing factors (SFs) that may have cell type-specific roles in *TERT* AS regulation and telomerase activity.

## Materials and methods

### Cell culture and cell lines

Calu-6 cells (RRID:CVCL_0236) were cultured at 37°C in 5% CO_2_ in 4:1 DMEM:Medium 199 containing 10% calf serum (HyClone, Logan, UT). Cell lines were obtained as a kind gift from Drs. John Minna and Adi Gazdar.

### iPSC culture and NPC differentiation protocol

Cellartis® Human iPSC Lines from Takara (ChiPSC22, Cat. No. Y00320) were cultured with strict adherence to manufacturer’s protocols and manuals. Cellartis® DEF-CS 500 (Y30017) culture system was employed to maintain iPSC cultures (thawing, passages, media changes and cryopreservation).

Generation and culturing of Neural Progenitor Cells (NPCs) was achieved with the STEMdiff® Neural System from STEMCELL Technologies. Briefly, STEMdiff® SMADi Neural Induction kit (Cat. No. 08581) was used to treat iPSC in culture according to manufacturer’s protocol that generates CNS-type NPCs. Following induction, NPC cultures were maintained with STEMdiff® Neural Progenitor Medium system (Cat. No. 05833). We performed the induction and selection according to the “monolayer culture protocol”. We considered Day 10 post-NPC induction as early-stage NPCs and day 29 as late-stage NPCs. Pellets were collected and population doublings were determined post-differentiation (~Day 15–20).

### Transient siRNA experiments

iPSCs were plated in 6-well plates (450,000 cells per well) and were transfected with non-silencing controls (Santa Cruz Biotechnology, sc-37007) or a pool of siRNAs targeting (Santa Cruz Biotechnology, PTBP1 sc-38280, NOVA1 sc-42142, PTBP2 sc-78824, SRSF2 sc-38317, U2AF2 sc-37667, and HNRNPM sc-38286). The iPSCs and Calu-6 cells were plated 24 h prior to transfections and transfection complexes were prepared with 10 nM of siRNAs using Opti-MEM (Gibco) and RNAi max (Invitrogen) following the manufacturer’s procedures. Following 72 h of exposure to siRNAs, cells were washed, trypsinized, counted and pelleted for downstream assays.

### Cell density experiments

iPSCs were plated on 6-well plates with 6 different cell densities: 250,000 cells; 400,000 cells; 500,000 cells; 750,000 cells; 1,000,000 cells; and 1,500,000 cells. Calu-6 cells were plated on 10-cm plates with three different cell densities: 816,000 cells; 1,600,000 cells; and 3,200,000 cells. After 48 hours, cell pellets were collected for analysis.

### Western blot analysis

**Fig 1:**
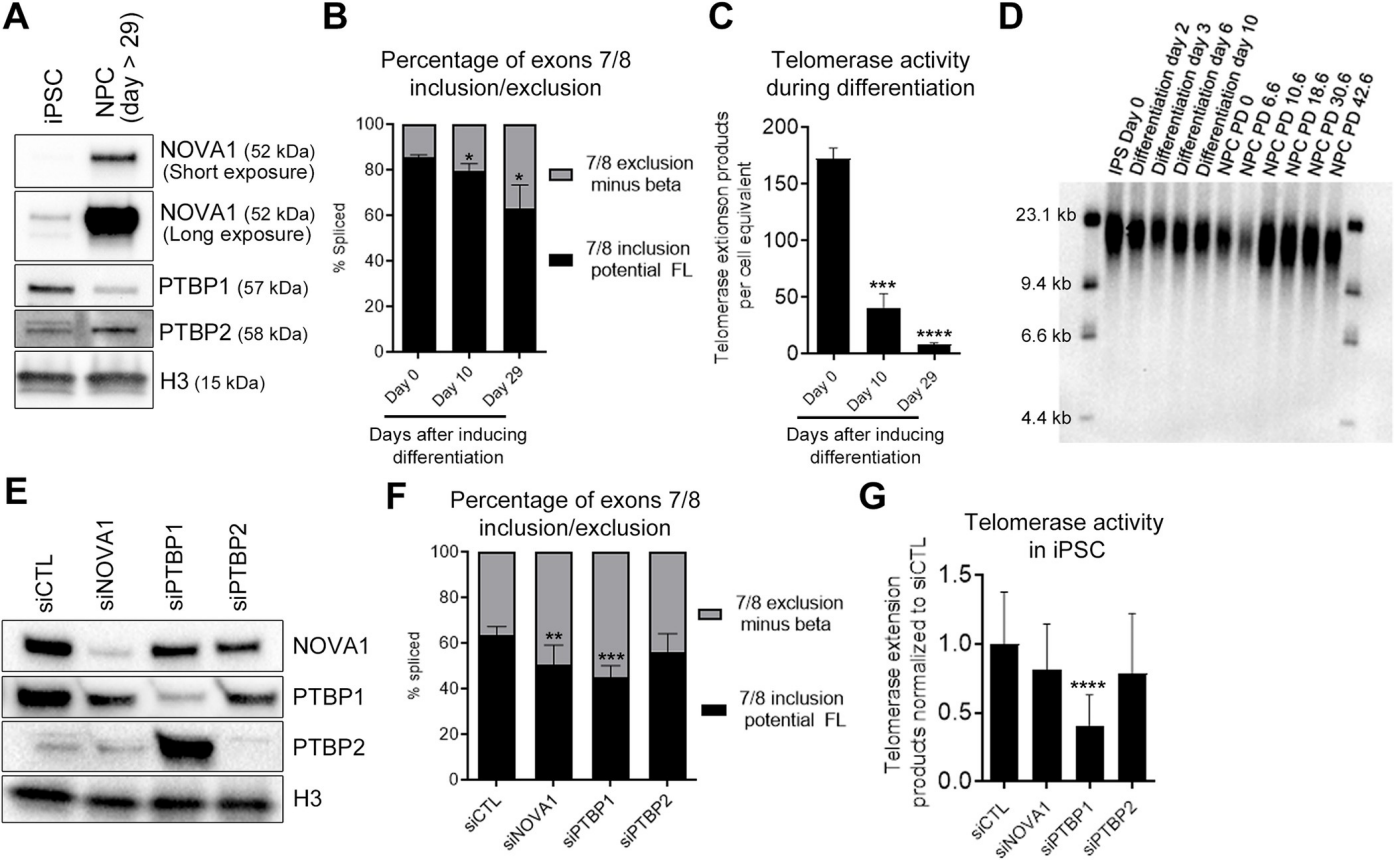
NOVA1-PTBP1-PTBP2 axis regulates *TERT* splice variant expression in differentiation of induced pluripotent stem cell (iPSC) to neural progenitor cell (NPC). A) Western blot of NOVA1, PTBP1 and PTBP2 in iPSC differentiation into NPC. H3 protein expression was used as a loading control for western blots. B) Reduction of exons 7/8 including *TERT* splice variant expression during differentiation compared to iPSCs (day 0) (determined by ddPCR; n = 3 biological replicates per condition). C) Reduction of telomerase activity during differentiation compared to iPSCs (day 0) (determined by ddTRAP; n = 3 biological replicates per condition). D) Terminal restriction fragment (TRF) Southern blot analysis displaying telomere lengths in iPSC at day 0 through NPCs PD 42.6. First and last lanes are molecular weight markers used to determine the sizes of TRFs in samples during the differentiation time course. iPSC was fully differentiated into NPC by day 10. E) Western blot image of siRNA-induced NOVA1, PTBP1 and PTBP2 knockdown in human iPSC. F) Transient siRNA-induced knockdown of NOVA1 and PTBP1, but not PTBP2, significantly shifted *TERT* gene expression from potential FL *TERT* (exons 7/8 included) to alternatively spliced minus beta *TERT* (exons 7/8 excluded) in iPSCs (determined by ddPCR; n = 5 biological replicates per condition). G) Transient knockdown of PTBP1, not NOVA1 nor PTBP2, significantly reduced telomerase enzyme activity in iPSCs (determined by ddTRAP; n = 5 biological replicates per condition). One-way ANOVA with uncorrected Fisher’s LSD for post hoc comparisons of treatments were used to compare Day 10 and Day 29 with Day 0 (B, C) and siRNA-treated conditions with siControl (F, G; *, *P* < 0.05; **, *P* < 0.01; ***, *P* < 0.001; ****, *P* < 0.0001). Data are presented as means ± standard deviations where applicable.

Cell pellets were collected and lysed in 40 μL of lysis buffer (NP40 based buffer) per 1 x 10^6^ cells. Total protein lysates were further treated with 40 μL of 2 x Laemmli buffer (Bio-Rad) and boiled for 10 mins at 95°C. Each protein lysate was loaded onto a polyvinylidene fluoride (PVDF) membrane by equal volume. Each blot was probed for beta-actin and based upon beta-actin band densitometry, lysate loading volumes were adjusted for all subsequent target protein westerns. Prepared lysates were resolved by SDS-polyacrylamide gel electrophoresis, transferred to PVDF membranes, and detected with antibodies for NOVA1 (rabbit monoclonal [EPR13847], Abcam, ab183024, 1:1000 dilution in 5% NFDM), PTBP1 (rabbit monoclonal [EPR9048B], Abcam, ab133734, 1:10,000 dilution in 5% NFDM), PTBP2 (Abcam, EPR9891, ab154853, 1:1000 dilution in 5% NFDM), SRSF2 (rabbit polyclonal [EPR12238], Abcam, ab204916, 1:1000 in 5% BSA), U2AF2 (rabbit polyclonal, Sigma-Aldrich, HPA041943, 1:1000 in 5% BSA), SRPK1 (rabbit polyclonal, Abcam, ab90527, 1:1000 in 5% BSA), CDC40 (rabbit monoclonal [EPR12539], Abcam, ab175924, 1:1000 in 5% BSA), HNRNPA1 (mouse monoclonal [9H10], Sigma-Aldrich, R4528, 1:1000 in 5% BSA), HNRNPA2B1 (mouse monoclonal [DP3B3], Abcam, ab6102, 1:1000 in 5% BSA), HNRNPCL1 (rabbit polyclonal, Abcam, ab129762, 1:1000 in 5% BSA), HNRNPH/HNRNPH1 (rabbit polyclonal [50–249], Acris Antibodies, AP19044PU-N, 1:2000 in 5% BSA), and HNRNPM (mouse monoclonal [1D8], Thermo Fisher Scientific, MA1-34981, 1:1000 in 5% BSA). Protein loading was determined with antibodies against histone H3 (Anti-Histone H3 antibody produced in rabbit, H0164; Sigma) for Fig 1, and beta-actin (mouse monoclonal [8H10D10], Cell Signaling Technology, 3700, 1:1000 in 5% BSA) and GAPDH (rabbit monoclonal [14C10], Cell Signaling Technology, 2118, 1:1000 in 5% BSA) for the rest experiments. Blots were imaged with Bio-Rad ChemiDoc XRS+ Molecular Imager and quantified with Bio-Rad Image Lab software. Normalized splicing factor (SF) protein expression levels were used for correlational assays (Fig 5 and S4 Fig). Once all lysates were probed for target genes, stripped membranes were probed for beta-actin or GAPDH to confirm equal loading. Values from the loading control blots were averaged and used to normalize the target protein quantification. These normalized protein expression values were expressed relative to the 400,000-cell density condition to generate the final values analyzed in the correlations. All images that were used to quantify the SF protein expression levels are included in S1 Raw images.

### Droplet digital TRAP assay (telomerase activity)

Droplet digital telomerase repeated amplification protocol (ddTRAP) assay was performed as described in Ludlow et al. [28]. Briefly, cells were lysed, diluted, and added to the extension reactions for 60 min followed by a 5-min heat inactivation of telomerase at 95°C. An aliquot of the extension product was amplified in a droplet digital PCR (ddPCR) for 40 cycles. Fluorescence intensity was measured, and droplets were counted on the droplet reader (QX200, Bio-Rad). Data was then calculated to represent telomerase extension products per cell equivalent and/or normalized to control conditions.

### Reverse transcription-ddPCR

RNA was extracted from frozen cell pellets using RNeasy® Plus Universal Mini Kit (Qiagen, 73404) according to manufacturer’s protocol. For TERT splicing analyses we used SuperScript IV First-Strand Synthesis System (Thermo Fisher) to generate cDNAs and used within 48 hours of production in ddPCR measures. 1 μg of RNA was used to synthesize cDNAs. All cDNAs were diluted to 1:4 (20 μL of cDNA + 60 μL nuclease-free water) before use and stored at -20°C. Primer sequences to target *TERT* splice variants (potential FL, minus beta, minus alpha, INS3, and INS4) and methods for calculating percent spliced *TERT* transcripts are from Ludlow et al. [16]. Total amount of *TERT* transcript was estimated by summing transcript level of exons 7/8 including (potential FL) and excluding (minus beta) *TERT*. Primers to measure intron 11 retention and intron 14 retention of *TERT* are from Dumbović et al. [25]. Primers to target minus gamma are Forward: 5’-ACATGGAGAACAAGCTGTTTGCG-3’ targeting exon 9 and Reverse: 5’-CGGGCATAGCTGAGGAAGGT-3’ targeting exons 10/12 junction. Primers to target plus gamma are Forward: 5’-ACATGGAGAACAAGCTGTTTGCG-3’ targeting exon 9 and Reverse: 5’-GGAAGTTCACCACTGTCTTCCGC-3’ targeting exon 11. Primers to measure Del2 *TERT* are Forward: 5’-TACCGCGAGGTGCTGCCGCTGGCCACGTTC-3’ targeting exon 1 and Reverse: 5’-CAGGATCTCCTCACGCAGCA-3’ targeting exon 3. To quantify skipping of *TERT* exon 2 (Del2 *TERT*) we used two separate PCR reactions with 5’ hydrolysis probes targeting either the exons 1/3 junction to detect Del2: 5’ 6-FAM-TCCTTCCGC/ZEN/CAGGGGTTGGCTGTG/blackhole quencher or a probe targeting exon 2: 5’ HEX-CAGCCGAAG/ZEN/TCTGCCGTTGCCCAAGA/black hole quencher. Primer validations using 2x EmeraldAmp® MAX HS PCR Master Mix (TaKaRa, RR330) are included in supporting information (S1 File). cDNAs for OCT4, NANOG, NES, and SOX1 were generated using iScript Advanced (Bio-Rad) and used in the same manner as mentioned above for *TERT* (except cDNAs for quantification of OCT4 and NANOG which cDNAs were diluted 1:50).

### Telomere length analysis

The average length of telomeres (terminal restriction fragment lengths) was measured as described in Mender and Shay’s study [29] with the following modifications: DNA was transferred to Hybond-N+ membranes (GE Healthcare, Piscataway, NJ) using overnight gravity transfer. The membrane was briefly air-dried and DNA was fixed by UV-crosslinking. Membranes were then probed for telomeres using a digoxigenin (DIG)-labeled telomere probe generated in-house [30] detected with a horseradish peroxidase-linked anti-DIG antibody (Roche, Cat. No. 11093274910), and exposed with CDP-star (Roche, Cat. No. 11759051001) and were imaged with Bio-Rad ChemiDoc XRS+ Molecular Imager.

### Bioinformatics and statistical analyses

Unless otherwise noted, one-way ANOVA with uncorrected Fisher’s LSD for post hoc comparisons were used to determine statistical significance between experimental groups. For bioinformatics analysis, RSEM values of each gene from 58 patients were downloaded from The Splicing variant Database [31] and Log2-transformed to generate box plots. Paired Student’s *t* test was used for two group comparisons (normal tissue versus solid tumor in Fig 4 and S3 Fig). Pearson’s correlations were utilized to examine the relationship of two factors (S2 Fig, ratio of exons 7/8 including *TERT* vs telomerase activity; Fig 5 and S4 Fig, ratio of exons 7/8 including *TERT* and expression levels of SF proteins). Statistical significance was defined as a *p* value ≤ 0.05.

## Results

### NOVA1-PTBP1-PTBP2 axis regulates *TERT* exons 7/8 alternative splicing during differentiation of induced pluripotent stem cells to neural progenitor cells

Sayed et al. [27] previously elucidated an alternative splicing (AS) regulatory mechanism of *TERT* by a NOVA1-PTBP1-PTBP2 axis in NSCLC cells. Briefly, NOVA1 recruits PTBP1 to DR8 (*cis*-element of *TERT* located in intron 8, direct repeat 8 (DR8)) to promote full-length (FL) *TERT* production. When PTBP1 is reduced, PTBP2 becomes abundant due to reciprocal regulation of PTBP2 by PTBP1 (PTBP1 represses expression of PTBP2 in non-neuronal tissues [32]). Then NOVA1 recruits PTBP2 to DR8 instead of PTBP1 to induce exon skipping of *TERT* resulting in reduced FL *TERT* splicing and reduced telomerase activity. We investigated whether this *TERT* AS regulatory mechanism is conserved during differentiation of induced pluripotent stem cells (iPSCs) to neural progenitor cells (NPCs) because NOVA1 and PTBP2 are highly expressed in neuronal cells [33–35]. Differentiation of iPSC to NPC was confirmed by loss of stem cell pluripotency markers (OCT4 and NANOG) and increased NPC markers (SOX1 and NES) using immunocytochemistry (S1A Fig) and RT-PCR (S1B–S1E Fig). Differentiation increased NOVA1 as expected, PTBP1 expression decreased, and PTBP2 expression increased in NPCs compared to iPSCs (Fig 1A). We observed fewer exons 7/8 including (potential FL) *TERT* transcripts with higher expression of NOVA1 and PTBP2 (Fig 1B). In addition, both total *TERT* expression (S1F Fig) and telomerase activity decreased during differentiation (Fig 1C and S1G Fig). We also measured Del2 *TERT* during differentiation and observed a significant increase in Del2 (S1H Fig). Next, we measured telomere length using terminal restriction fragment (TRF) analysis from the first day of differentiation until ~40 population doublings of NPCs. While iPSCs were able to maintain their telomeres at around 18 kb, telomere length started to shorten as they progressed into the NPC lineage (Fig 1D). To mechanistically confirm that the NOVA1-PTBP1-PTBP2 axis regulates *TERT* AS in iPSCs, loss-of-function (siRNA) experiments were performed. We confirmed siRNA knockdown efficiency by western blotting, indicating robust reduction in target protein levels (Fig 1E, S1I–S1K Fig). When NOVA1 is reduced by 80%, PTBP1 expression also significantly decreased by 39% (S1I and S1J Fig). When PTBP1 was reduced by 82%, PTBP2 expression significantly increased by 6.5-fold compared to control-treated iPSCs, as expected (S1J and S1K Fig) [32]. When PTBP2 is reduced by 84%, NOVA1 and PTBP1 expressions also significantly decreased by 42% and 47%, respectively (S1I–S1K Fig). When treated with PTBP1- or NOVA1-targeting siRNAs, the ratio and transcript level of potential FL *TERT* (exons 7/8 including *TERT*) were also significantly reduced compared to control-treated cells (Fig 1F, S1L Fig). Only PTBP1 knockdown resulted in significantly reduced telomerase activity compared to control-treated iPSCs (Fig 1G). On the other hand, we did not observe significant changes of *TERT* or telomerase when PTBP2 was knocked down (Fig 1F and 1G, S1L and S1M Fig). In summary, these results support that the NOVA1-PTBP1-PTBP2 axis regulates *TERT* AS in iPSCs and during differentiation of iPSCs to NPCs.

### Impact of iPSC cell density on expression of *TERT* splice variants

**Fig 2:**
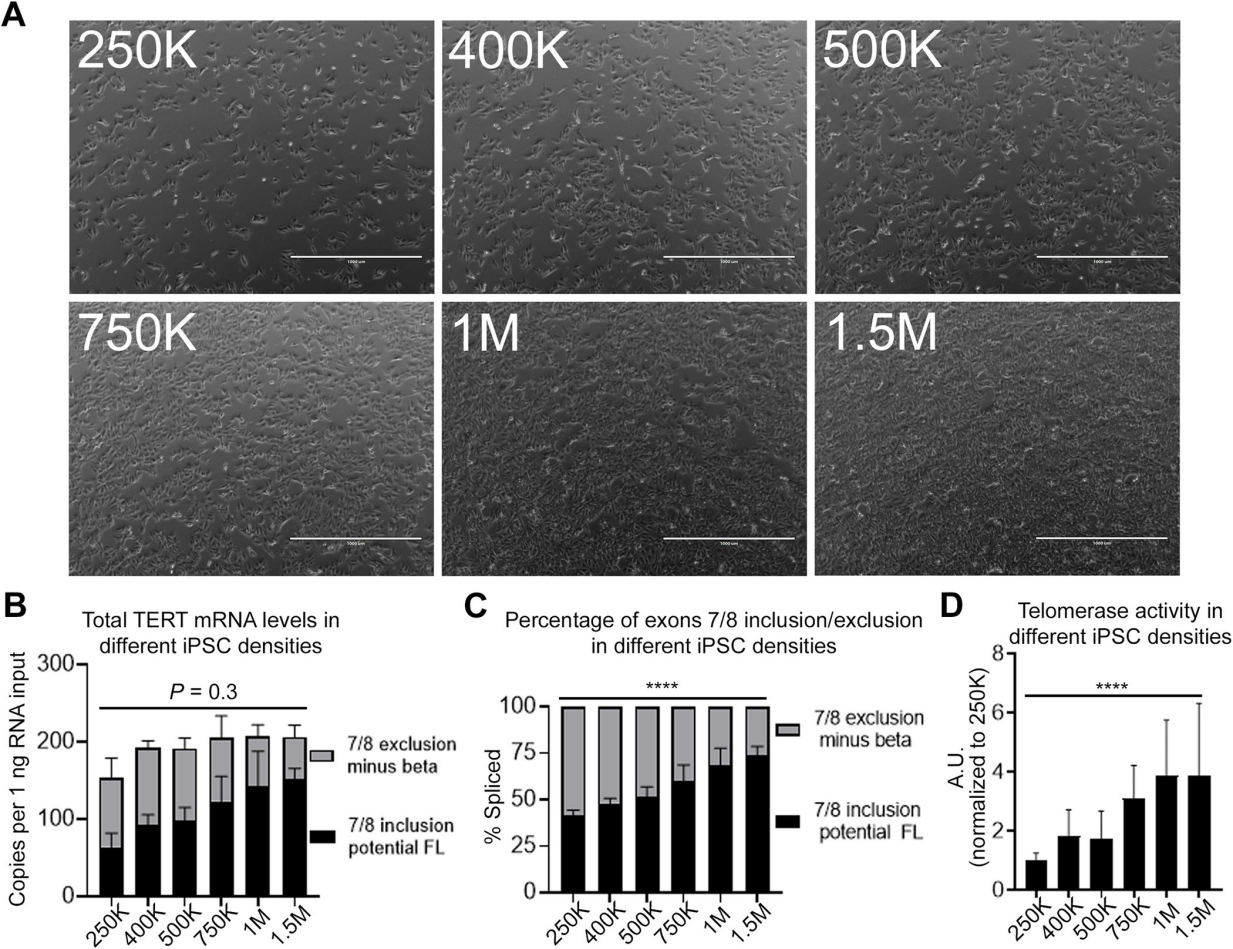
Impact of iPSC cell density on expression of *TERT* splice variants. A) Representative phase contrast micrograph, 24 hours after seeding. The number of seeded iPSCs are indicated. Scale bar for image is 1000 μm. B and C) *TERT* gene expression is shifted from alternatively spliced, exons 7/8 excluding *TERT* (minus beta), to exons 7/8 including *TERT* (potential FL) with higher iPSCs density (determined by ddPCR; n = 4 biological replicates per condition). Transcript copies per 1 ng RNA input (B) and splicing ratio (C) were quantified. D) Telomerase activity significantly increased with higher iPSC density compared to lowest cell density condition (determined by ddTRAP; n = 4 biological replicates per condition). One-way ANOVA was performed to compare total amount of *TERT* including/excluding exons 7/8 (B), ratio of exons 7/8 including *TERT* splice variants (C), and telomerase activity (D) of all conditions (****, *P* < 0.0001). Data are presented as means ± standard deviations where applicable.

Understanding the regulation of *TERT*’s reverse transcriptase domain (exons 4–13) is important for understanding the generation of telomerase active *TERT*. Therefore, in the following experiments, we focused on alternative splicing within this region. While we were testing different cell seeding densities of iPSCs for siRNA knockdown experiments, we noticed that the ratio of exons 7/8 including *TERT* appeared to change depending on cell density. We hypothesized that higher cell density resulted in higher percentage of exons 7/8 including *TERT*. To test our hypothesis, we seeded different numbers of iPSCs and collected the cells 48 hours after (Fig 2A). Although the amount of total *TERT* transcripts (sum of exons 7/8 inclusion (potential FL) and exons 7/8 exclusion (minus beta)) did not change significantly (Fig 2B), the ratio of exons 7/8 including *TERT* increased along with cell density (Fig 2C). With higher cell density, telomerase activity was also increased and positively correlated to the ratio of exons 7/8 including *TERT* (Fig 2D, S2H Fig), supporting that the increase of exons 7/8 including *TERT* transcripts represents increased production of FL *TERT* transcripts (i.e., telomerase coding *TERT*). We also measured other known *TERT* splice variants that include exons 7/8 such as minus alpha (exon 6 partial exclusion), INS3, INS4, minus gamma (exon 11 exclusion), plus gamma (exon 11 inclusion), intron 11 retention, and intron 14 retention to rule out changes in other splice variants explaining our observations. Expression of the tested splice variants that also contain exons 7/8 were not impacted significantly by cell density (S2A–S2G Fig). In summary, these data suggest that alternative splicing regulation of exons 7/8 skipping in iPSC is impacted by cell density. Higher cell densities induce higher expression of FL *TERT* resulting in higher telomerase activity without an increase in total *TERT* expression.

### Impact of Calu-6 cell density on expression of *TERT* splice variants

**Fig 3:**
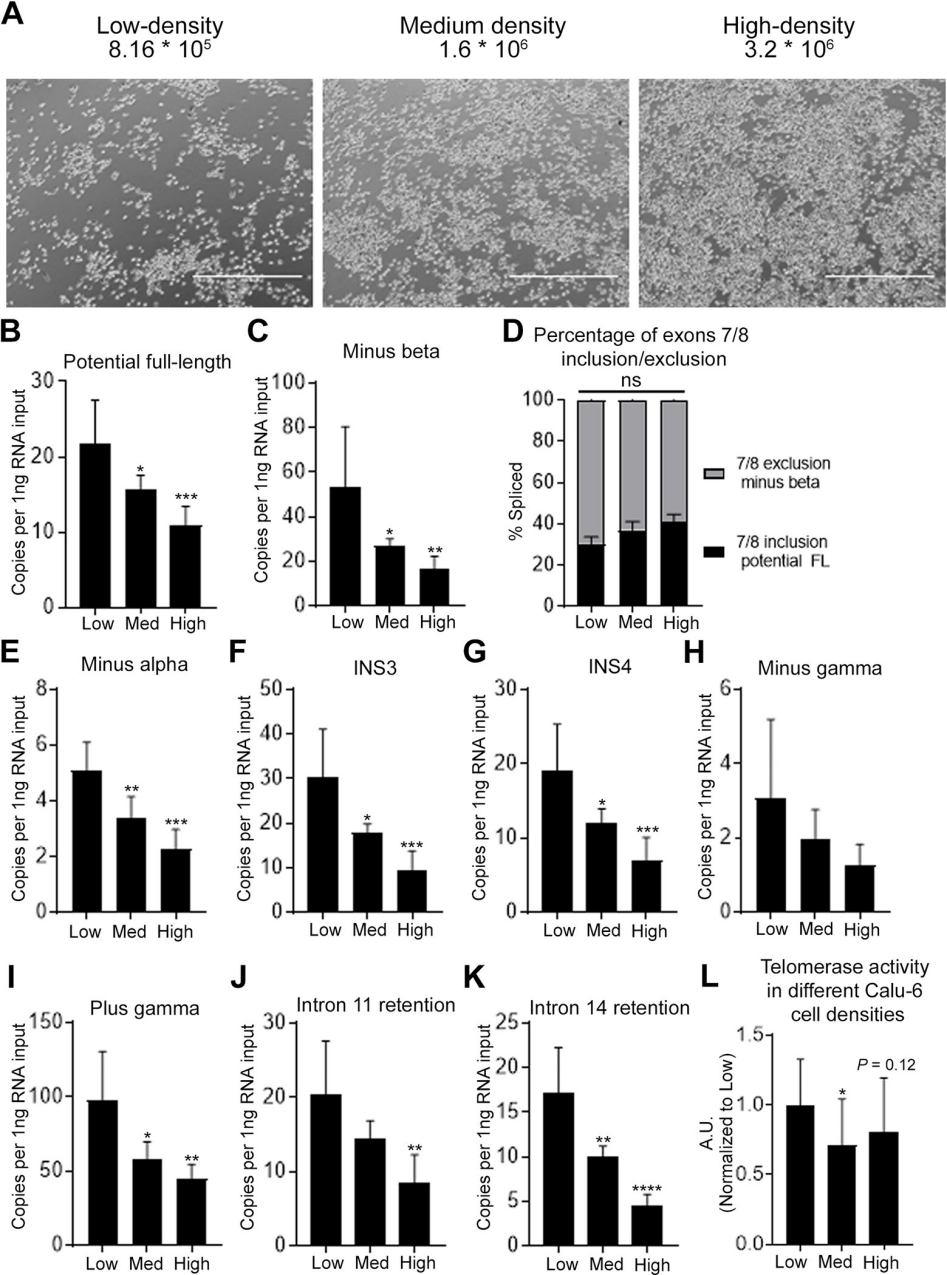
Impact of Calu-6 cell density on expression of *TERT* splice variants. A) Representative phase contrast micrograph images 24 hours after seeding. The number of seeded Calu-6 cells are indicated. Scale bar for image is 1000 μm. B,C, and E-K) Expression of *TERT* splice variants are reduced with higher cell density. Potential FL (B), minus beta (C), minus alpha (E), INS3 (F), INS4 (G), minus gamma (H), plus gamma (I), intron 11 retention (J), and intron 14 retention (K) transcripts were quantified (determined by ddPCR; n = 6 biological replicates per condition). D) Exons 7/8 alternative splicing ratio did not change significantly in different cell densities. L) Telomerase activity in different cell densities (determined by ddTRAP; n = 6 biological replicates per condition). One-way ANOVA with uncorrected Fisher’s LSD for *post hoc* comparisons were used to compare medium (Med) and high-density (High) conditions with low-density (Low) condition (B-L; ns, *P* > 0.05; *, *P* < 0.05; **, *P* < 0.01; ***, *P* < 0.001; ****, *P* < 0.0001). Data are presented as means ± standard deviations where applicable.

Next, we aimed to determine if cell density impacted cancer cell *TERT* splice variant expression similar to iPSCs. To test our hypothesis, we seeded different numbers of Calu-6 lung cancer cells and collected 48 hours after (Fig 3A). We observed significant reduction in both exons 7/8 including and excluding *TERT* transcripts (Fig 3B and 3C) while we did not observe significant changes in the splicing ratio of exons 7/8 (Fig 3D). Next, we measured the same *TERT* splice variants that we measured in iPSCs, and all splice variants were decreased in the medium and high-density conditions compared to the low-density condition (Fig 3E–3K). Telomerase activity was significantly reduced in medium density compared to low-density, but low- and high-density were not significantly different from each other (Fig 3L). Clearly, overall *TERT* transcripts decreased with an increased cell density in Calu-6 cells. Also, higher cell density resulted in reduction of telomerase activity in Calu-6 cells, which is opposite to our observations in iPSCs.

### Identification of candidate splicing factors that potentially regulate *TERT* splice variant expression differently between iPSCs and lung cancer cells

**Fig 4:**
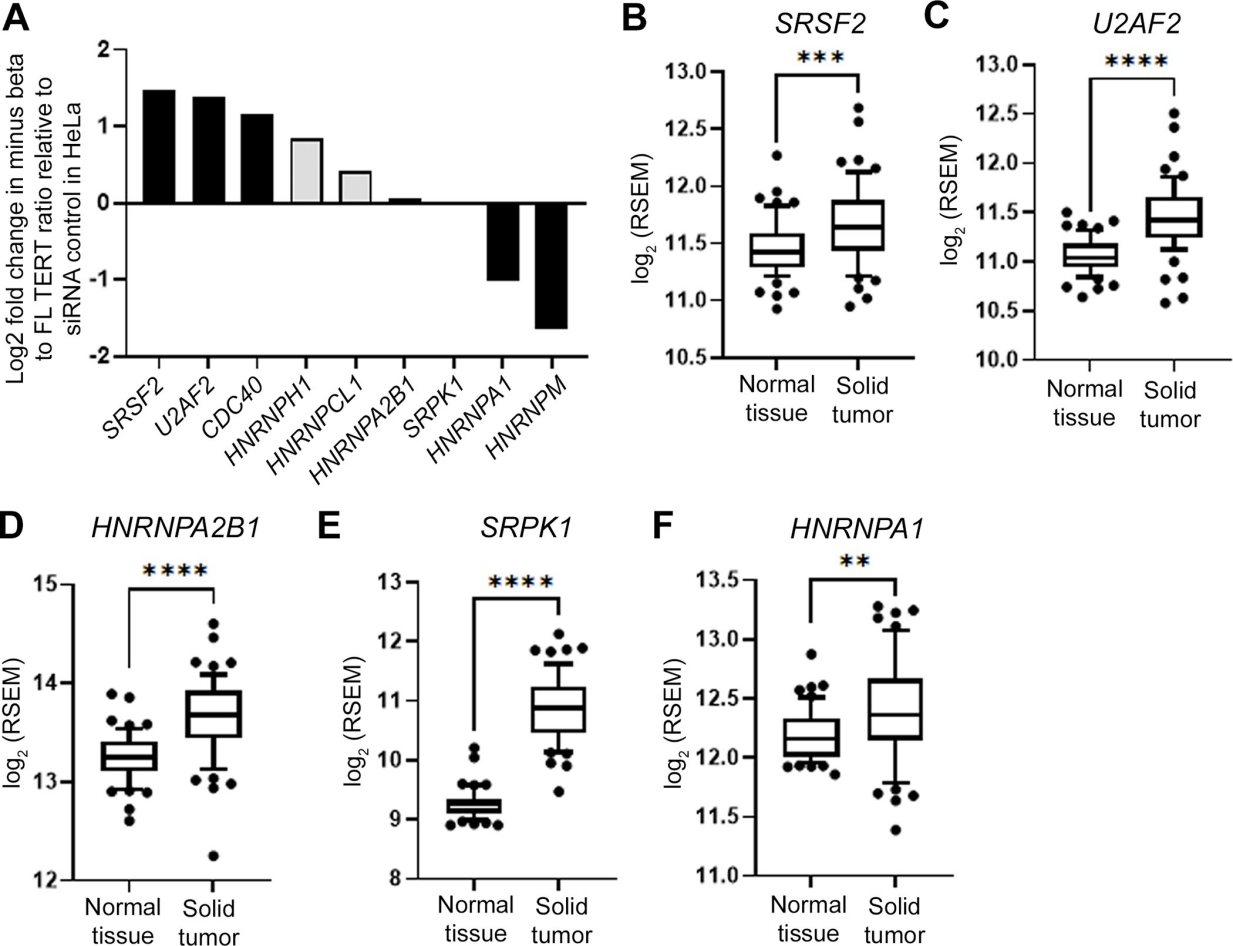
Analysis of *TERT* AS minigene loss-of-function screen and patient tumor expression profiles of splicing factors identifies top candidate SFs related to *TERT* splicing in lung cancer cells. A) *TERT* minigene data of selected SFs showing effect of SF knockdown on *TERT* exons 7/8 splicing in HeLa cells [16]. B-F) Log2-transformed RSEM values of SFs gene-expression levels (n = 58 matched LUAD patient samples). SRSF2 (B), U2AF2 (C), HNRNPA2B1 (D), SRPK1 (E), and HNRNPA1 (F) are significantly upregulated in tumor tissues from LUAD patients compared to tumor-adjacent normal tissues. Paired Student’s *t* test set at *P* ≤ 0.05 for significance compared with normal tissue controls (B-F; ** *P* < 0.01; *** *P* < 0.001; **** *P* < 0.0001). In the box plots, the lower boundary of the box indicates the 25th percentile, a line within the box marks the median and the higher boundary of the box indicates the 75th percentile. Whiskers above and below the box indicate the 10th and 90th percentiles. Points above and below the whiskers indicate data outside the 10th and 90th percentiles.

We utilized our *TERT* minigene loss-of-function (siRNA) screening data in HeLa cells from Ludlow et al. [16] and publicly available TCGA (The Cancer Genome Atlas) data from LUAD (lung adenocarcinoma) patients [31] to select candidates regulating *TERT* AS for further study. Briefly, Ludlow et al. [16] measured the ratio of luciferase expression that indicated inclusion or exclusion of exons 7/8 from a *TERT* reporter minigene after siRNA knockdown of splicing factors in HeLa cells to find enhancers/repressors of telomerase. From the minigene data, we selected three SFs (SRSF2, U2AF2, and CDC40) that promoted FL *TERT* production more than 2-fold and had predicted binding sites in the *TERT* gene between exons 5–9. We also selected two SFs (HNRNPA1 and HNRNPM) because they promoted minus beta *TERT* production more than 2-fold and have predicted binding sites in the *TERT* gene between exons 5–9 (Fig 4A). We also included four additional splicing regulatory genes (HNRNPA2B1, HNRNPH1, HNRNPCL1, and SRPK1) because they are either related to cancer in general or have been associated with telomere biology (HNRNPA2B1: [36]; HNRNPH1: [37]; HNRNPCL1: [38]; SRPK1: [39]).

The TCGA data provides gene expression levels (RSEM values from RNA-Seq) from tumor tissues and tumor-adjacent normal tissues of LUAD patients. Since we recently published that only TCGA LUAD tumor samples express FL *TERT* and tumor adjacent normal tissues do not express FL *TERT* [40], we surmised that highly expressed splicing factors would be related to the re-emergence of FL *TERT* in the cancerous tissue. Using this logic, we investigated the expression of the minigene-selected SFs in the paired-normal-tumor-samples from the TCGA data (Fig 4B–4F). Among the selected SFs, we identified five SFs that had significantly increased expression in the tumor samples compared to normal tissues (SRSF2 (*P* < 0.01), U2AF2 (*P* < 0.01), HNRNPA2B1 (*P* < 0.01), SRPK1 (*P* < 0.01), and HNRNPA1 (*P* < 0.01), Fig 4B–4F). Four SFs were not significantly different between matched normal and tumor samples (CDC40, HNRNPH1, HNRNPCL1, and HNRNPM, S3A–S3D Fig). We used these data as the foundation for an antibody expression screen of the same proteins in our cell density experiments with the iPSCs as a means to identify SFs that regulate TERT differently or similarly between stem and cancer cells.

### Impact of iPSC cell density on SF expression of *TERT* regulating candidate SFs

**Fig 5:**
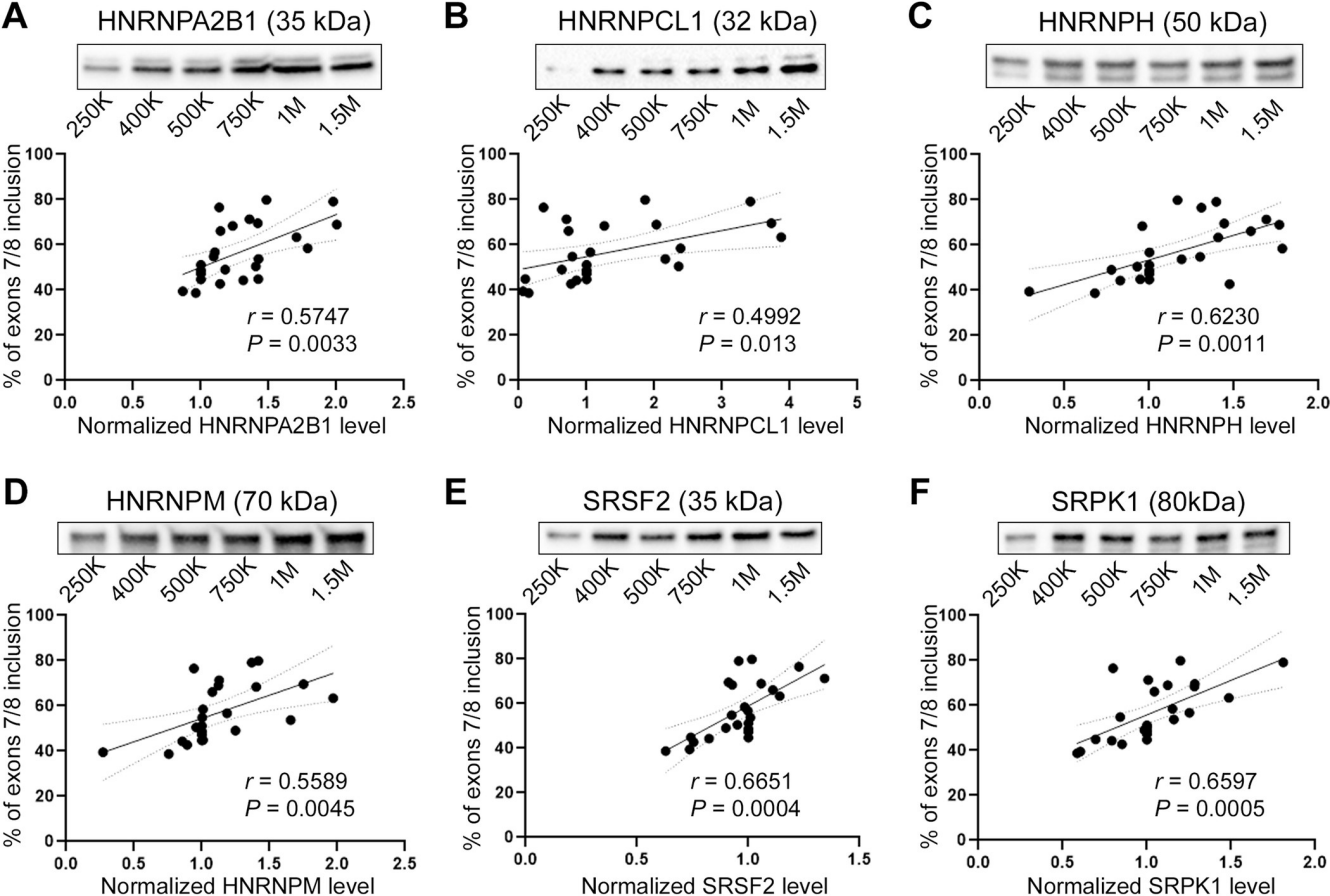
Impact of iPSC cell density on SF expression of *TERT* AS candidate SFs. A-F) Western blots of splicing factors and correlation analyses in different iPSC density. Top are representative images and bottom are scatter plots showing correlations between housekeeping gene-normalized SF expression levels and percentage of *TERT* exons 7/8 inclusion (n = 4 biological replicates per condition). Antibodies targeting HNRNPA2B1 (A), HNRNPCL1 (B), HNRNPH (C), HNRNPM (D), SRSF2 (E), and SRPK1 (F) were used for the western blots. 95% Confidence bands, Pearson’s correlation coefficient (*r*) and *p* values are shown. For the correlation analysis, 24 data points are included (six conditions x four replicates).

Since *TERT* AS was significantly altered in the iPSC cell density experiment, we surmised that SFs that correlated with changes in potential FL *TERT* would potentially regulate *TERT* AS in stem cells. We measured protein levels of the nine SFs (SRSF2, U2AF2, CDC40, HNRNPA1, HNRNPM, HNRNPA2B1, HNRNPH1, HNRNPCL1, and SRPK1) in the iPSCs cell density experiment. Using SF protein level data and the *TERT* mRNA splice variant expression data we carried out correlational analysis to reveal potential relationships between percentage of exons 7/8 including *TERT* and SFs. Expression level of six splicing factors (HNRNPA2B1, HNRNPCL1, HNRNPH, HNRNPM, SRSF2, and SRPK1) showed significant positive correlations with the percentage of exons 7/8 inclusion (potential FL *TERT*; Pearson’s correlation *r* = 0.5747, *P* = 0.0033; *r* = 0.4992, *P* = 0.013; *r* = 0.6230, *P* = 0.0011; *r* = 0.5589, *P* = 0.0045; *r* = 0.6651, *P* = 0.0004; *r* = 0.6597, *P* = 0.0005, respectively; Fig 5A–5F), whereas three splicing factors (HNRNPA1, U2AF2, and CDC40) did not show significant correlation (S4A–S4C Fig).

Based on the *TERT* minigene study in HeLa cells, TCGA data in LUAD patients, and iPSC data, we classified the SFs into either FL *TERT*-promoting (green color), minus beta *TERT*-promoting (red color), or non-effector (no color) to summarize their predicted behavior in iPSCs and cancer cells (S1 Table). From this table, we selected HNRNPM as a stem cell-specific FL *TERT* promoter because it was only related to FL *TERT* expression in stem cells; SRSF2 as a FL *TERT* promoter in both stem cells and cancer cells because it was related to FL *TERT* expression in all three data sets; and U2AF2 as a cancer cell specific FL *TERT* promoter because it was only related to FL *TERT* expression in the minigene and TCGA data (yellow color highlighted in S1 Table). We also investigated correlation between the percentage of exons 7/8 including *TERT* and three RNA binding proteins: PTBP1, NOVA1, and PTBP2. However, we did not find a correlation in different cell densities (S4E–S4G Fig).

### Knockdown of candidate splicing factors (SFs) in cancer cells (Calu-6) results in expected shifts in *TERT* AS

**Fig 6:**
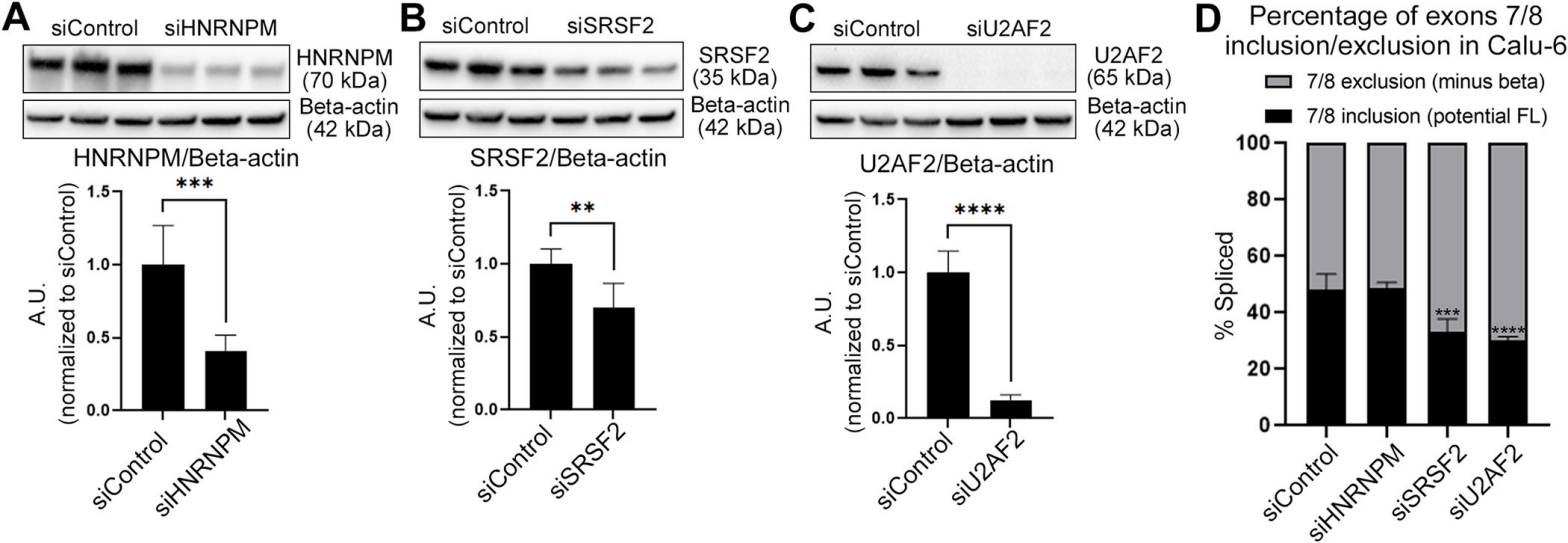
Knockdown of candidate splicing factors (SFs) in cancer cells (Calu-6) resulted in expected shifts in *TERT* splice variant expression. A-C) Knockdown of HNRNPM (A), SRSF2 (B), and U2AF2 (C) was confirmed by western blot. Representative images of selected SFs and beta-actin (loading control) (top panel). Knockdown was quantified by normalization with beta-actin then expressed relative to siRNA control (n = 6 biological replicates per condition; bottom panel). D) *TERT* splice variant expression ratio of exons 7/8 inclusion (potential FL) to exons 7/8 exclusion (minus beta) was reduced by knockdown of SRSF2 and U2AF2, not HNRNPM (determined by ddPCR; n = 6 biological replicates per condition). Student’s t test set at *P* ≤ 0.05 for significance compared with siRNA-treated conditions with siControl (A-C; **, *P* < 0.01; *** *P* < 0.001; **** *P* < 0.0001). One-way ANOVA with uncorrected Fisher’s LSD for *post hoc* comparisons of siRNA treatments were used to compare siRNA-treated conditions with siControl (D; ***, *P* < 0.001; ****, *P* < 0.0001). Data are presented as means ± standard deviations where applicable.

Based on our candidate selection process, three SFs are expected to be FL *TERT* promoters (HNRNPM in iPSCs; SRSF2 in both iPSCs and cancer cells; U2AF2 in cancer cells). To confirm our predictions, we performed loss-of-function (siRNA) studies in Calu-6 lung cancer cells. Knockdown by siRNAs of each splicing factor in Calu-6 cells was confirmed by western blotting (Fig 6A–6C). When HNRNPM expression was reduced, the ratio of exons 7/8 including *TERT* to exons 7/8 excluding *TERT* expression did not change significantly compared to control-treated cells, matching our predictions (Fig 6D). When expression of SRSF2 or U2AF2 was reduced, the ratio of exons 7/8 including *TERT* to exons 7/8 excluding *TERT* was reduced, indicating a reduction in FL *TERT* expression (Fig 6D). These outcomes of *TERT* AS also matched our predictions that SRSF2 and U2AF2 were FL *TERT* promoters in cancer cells, while HNRNPM would not impact splicing of *TERT* exons 7/8. The absolute expression of exons 7/8 including *TERT* transcripts were reduced with knockdown of all SFs compared to the scramble siRNA-treated cells (S5A Fig). Exons 7/8 excluding *TERT* transcripts were significantly reduced in HNRNPM and SRSF2 knockdown cells, while U2AF2 cells had similar expression levels compared to controls (S5B Fig). When compared to the scramble siRNA-treated cells, telomerase activity was significantly reduced by knockdown of all splicing factors (S5C Fig). This indicates that HNRNPM knockdown was indirectly reducing *TERT* expression, while SRSF2 or U2AF2 knockdown was likely more directly shifting *TERT* splice variant expression ratio, resulting in reduced telomerase activity. When protein expression levels of the three SFs were compared in iPSCs to NPCs, SRSF2 expression increased significantly whereas HNRNPM or U2AF2 expression did not change significantly (S5D–S5F Figs).

## Discussion

**Fig 7:**
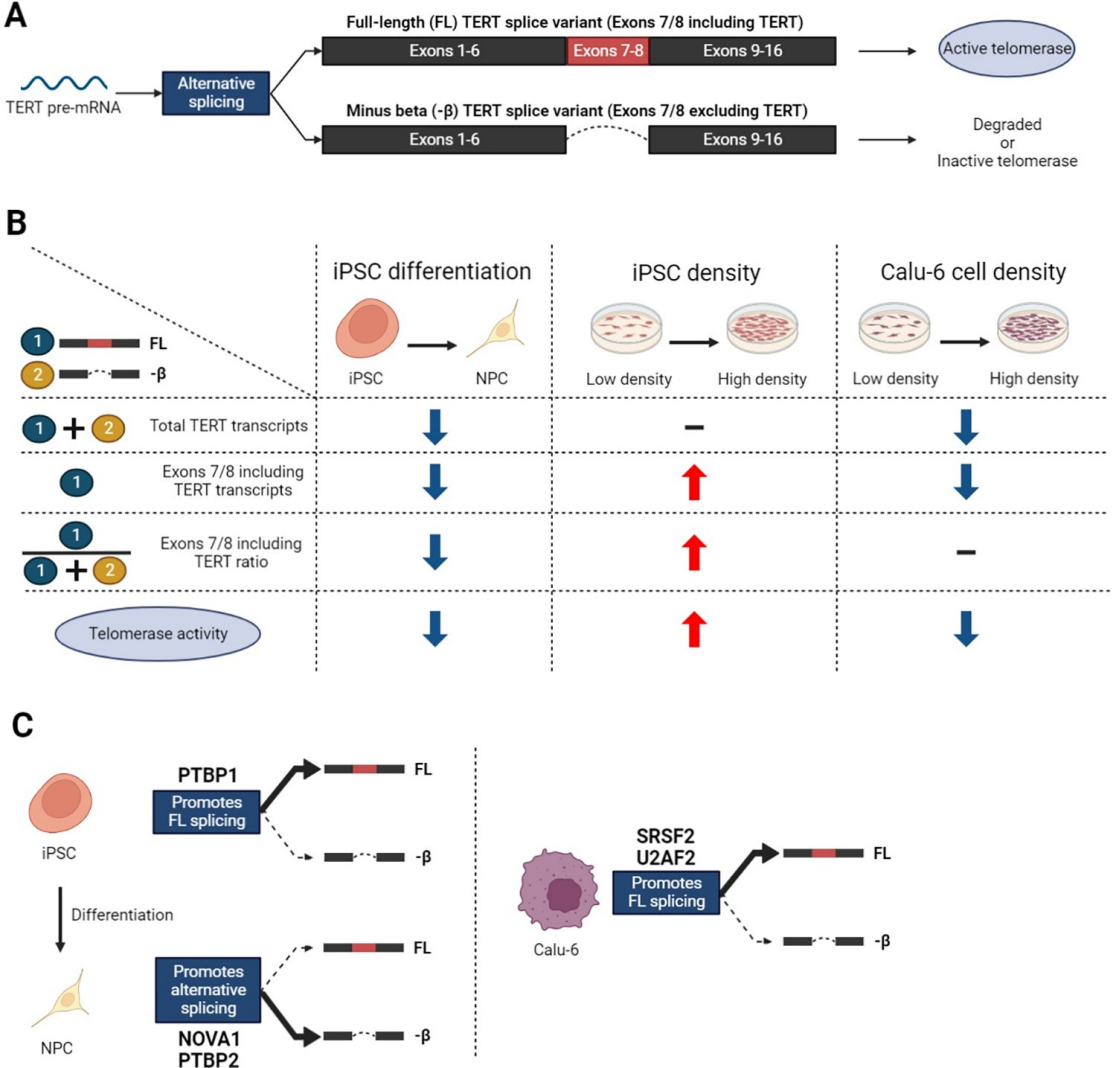
Working model figure describing splicing events of *TERT* observed in this study. A) Alternative splicing of *TERT* pre-mRNA produces splice variants. Only full-length *TERT* with all 16 exons can be translated into active telomerase. When exons are spliced out (e.g., exclusion of exons 7–8 in red box), transcripts are degraded or form inactive telomerase. B) *TERT* expression and telomerase activity in three different contexts (iPSC differentiation into NPC, increase in iPSC density, and Calu-6 cell density). Increase (red arrow) or decrease (blue arrow) or no change (—) are indicated for total *TERT* transcripts (FL *TERT* (7/8 included) plus minus beta *TERT* (7/8 excluded); ➀+➁), absolute amount of FL *TERT* transcript (7/8 included, ➀), percentage of FL *TERT* transcripts compared to total (➀/(➀+➁)), and telomerase activity. C) (Left panel) *TERT* AS regulators in iPSC differentiation into NPC or in calu-6 cells. PTBP1 promotes FL *TERT* in iPSCs whereas NOVA1 and PTBP2 promote minus beta *TERT* in NPCs. (Right panel) Three splicing factors were selected as cell type specific FL *TERT*-promoting candidates (HNRNPM in iPSCs; SRSF2 in both iPSCs and cancer cells; U2AF2 in cancer cells). *TERT* AS in Calu-6 cells was not affected by HNRNPM knockdown. On the other hand, SRSF2 or U2AF2 knockdown significantly reduced the amount and percentage of FL *TERT* (7/8 included). This figure was created with BioRender.com.

Understanding the precise control and regulation of telomerase activity is critical to both cancer and regenerative biology. Recent evidence from our laboratory and others has pointed out that in addition to transcriptional regulation, post-transcription mechanisms such as alternative RNA splicing are critical to the generation of telomerase-active TERT. Specifically, *TERT* pre-mRNAs are alternatively spliced to form various splice variants and only full-length *TERT* with all 16 exons can be translated into active telomerase (Fig 7A). On the other hand, other splice variants (such as minus beta *TERT*) are degraded by nonsense-mediated decay or translated into inactive telomerase that cannot synthesize telomeres [41]. In this research, we confirmed that *TERT* AS is regulated by the NOVA1-PTBP1-PTBP2 axis in iPSCs and iPSC differentiation into NPC. Next, we determined that *TERT* AS is impacted by cell density in stem cells (iPSCs) but not in cancer cells (Calu-6). Lastly, we identified splicing factors that are predicted to impact *TERT* splice variant expression only in cancer cells, only in stem cells, or in both cell types. Overall, our findings indicate that we may be able to decipher a regulated *TERT* AS code that controls telomerase in stem cells, and that cancer cells probably use several different codes to induce FL *TERT* and telomerase via alternative RNA splicing factor expression.

NOVA1 and PTBP2 are brain- and neural-specific splicing factors that help determine neural cell fate, while PTBP1 is a more ubiquitous splicing factor. While PTBP1 and PTBP2 share overlapping target genes, they also have separate targets and may induce opposite effects on shared target genes [42]. We have previously shown that when PTBP1 is knocked down, FL *TERT* and telomerase levels are reduced in non-small cell lung cancer (NSCLC) cells [27]. Specifically, we determined that PTBP1 interacts with NOVA1 to promote FL *TERT* and telomerase activity in lung cancer cells. However, when PTBP1 was knocked down in lung cancer cells, PTBP2 levels went up to compensate for reduced PTBP1 levels, and PTBP2 interacts with NOVA1 [43] and resulted in reduced FL *TERT* and telomerase. When we differentiated stem cells into NPCs we also observed increased NOVA1, increased PTBP2, reduced PTBP1, and reduced *TERT*. The amount of total *TERT* transcripts, the amount and percentage of exons 7/8 including *TERT* (FL *TERT*), and telomerase activity decreased with the differentiation (iPSC differentiation in Fig 7B), mimicking what we observed in NSCLC cells when PTBP1 was knocked down. We then tested this observation in loss-of-function studies in stem cells. We were able to replicate that when PTBP1 is reduced, PTPB2 is increased and likely interacts with NOVA1 to repress TERT and telomerase activity. In addition to PTBP1 knockdown, we also knocked down NOVA1 and PTBP2 by siRNA treatment in iPSCs. While the PTBP1 knockdown results replicated our previous study in cancer cells [27], NOVA1 knockdown did not result in significant reduction of telomerase activity in iPSCs despite the significant reduction of NOVA1 expression level. It only resulted in the reduction of the amount and percentage of exons 7/8 including *TERT*. When NOVA1 was knocked down, PTBP1 level also decreased significantly (~39%) whereas PTBP2 level did not change significantly. Considering the abundance of PTBP1 and NOVA1 in iPSCs, the reduction of exons 7/8 including *TERT* is likely to be mainly associated with PTBP1 reduction induced by indirect effects from NOVA1 knockdown. It also can be interpreted that either 1) the reduced level of NOVA1 expression is still sufficient to recruit PTBP1 to promote FL *TERT* or 2) NOVA1 does not affect the recruitment of PTBP1 in iPSCs. Further studies (e.g., overexpression of NOVA1 in iPSCs) are needed to confirm the recruitment of PTBP1 by NOVA1 in iPSCs. Overall, PTBP1 is an important regulator of *TERT* AS in iPSCs. When PTBP2 was knocked down, it resulted in non-significant changes in telomerase activity or *TERT* AS. In addition to the decrease in PTBP2 expression level, expression levels of NOVA1 and PTBP1 were also modestly reduced by PTBP2 knockdown. No reduction in exons 7/8 including *TERT* was observed despite the significant reduction of NOVA1 and PTBP1 expression, likely due to there being enough PTBP1 and NOVA1 protein remaining to maintain FL *TERT* expression levels. This indicates that there is likely a threshold of PTBP1 or NOVA1 reduction that must be reached before FL *TERT* is impacted. Despite the challenges of data interpretation due to apparent interdependent protein expression between NOVA1-PTBP1-PTBP2 the following conclusions can be still made: 1) a threshold of exons 7/8 including *TERT* reduction is needed to result in significant telomerase activity reduction; 2) PTBP1 is a major FL *TERT* promoter in iPSCs; 3) The increase in NOVA1 and PTBP2 with neuronal cell lineage differentiation is likely a strong cell fate determining mechanism that results in repressed *TERT* in neural tissues of humans (left panel in Fig 7C).

When we were empirically determining the cell density to seed stem cells at for siRNA loss-of-function studies, we noted that FL *TERT* splice variant expression tended to track with cell density. Indeed, when we tested different densities, we observed a striking switching in splice variant expression from mostly minus beta (exons 7/8 excluded) to mostly potential FL *TERT* (exons 7/8 included) with little changes in total *TERT* transcripts of the reverse transcriptase domain (exons 4–13) (iPSC density in Fig 7B). Next, we wanted to confirm if this cell density dependent switch in splicing was conserved in lung cancer cells. We observed reduced *TERT* total transcripts with little to no switching in splice variant expression in higher cell densities of Calu-6 lung cancer cells (Calu-6 density Fig 7B). These data indicate that regulation of *TERT* expression in stem cells may rely more heavily on *TERT* splice variant ratios compared to the lung cancer cells that relies more on transcriptional regulation to promote or repress TERT. This observation should be tested in more cancer cell lines to determine a general versus cell line specific phenomenon.

Next, we wanted to build upon the idea that there is a regulated *TERT* splicing code in stem cells and a dysregulated *TERT* splicing code in cancer cells. To do this we utilized our previously published *TERT* minigene splicing factor loss-of-function screen [16], TCGA public RNA-Seq data of splicing factor expression, and an antibody screen of *TERT* related splicing factors in stem cells. Of the nine splicing factors that we screened to be potential *TERT* regulatory factors, we observed that five splicing factors were related to *TERT* expression in lung cancer patient samples. Then we counter screened all nine factors in our stem cell density model by measuring their protein expression and correlated *TERT* expression to splicing factor expression. This analysis revealed that six splicing factors were correlated with FL *TERT* expression. The combination of these analyses revealed stem cell- and cancer cell-specific TERT regulatory factors (S1 Table). Based on our current (Fig 1) and previous observations, we also investigated the correlation between *TERT* expression and NOVA1, PTBP1, and PTBP2. However, the expression of these *TERT* AS regulators was not correlated to the expression of *TERT* in different stem cell densities. This indicates that the shifting of *TERT* AS in different cell density is not dictated by the NOVA1-PTBP1-PTBP2 axis.

Based on our screening data we next wanted to test our predictions of *TERT* cell type-specific regulators. We performed loss-of-function experiments in Calu-6 lung cancer cells. Our data revealed that our methods accurately predicted splicing factors that would impact *TERT* splice variant expression ratios in cancer cells (right panel in Fig 7C). To investigate whether the three splicing factors (HNRNPM, SRSF2, U2AF2) regulate *TERT* AS in stem cell differentiation into NPC, we compared protein expression levels of the splicing factors in iPSCs and NPCs. However, differentiation did not induce significant changes in the expression levels of HNRNPM and U2AF2. Moreover, increased SRSF2 expression in NPC indicates that SRSF2 is unlikely a FL *TERT*-promoting splicing factor in NPC. In addition to the three splicing factors we tested, other splicing factors and RNA binding proteins that regulate *TERT* alternative splicing have been identified by other studies. In studies using cancer cells, splicing of the *TERT* reverse transcriptase domain has been focused on because of its importance in telomerase activity [40, 41]. In stem cells, it was shown that skipping of *TERT* exon 2 is a developmental switch for TERT expression and is regulated by the splicing co-factor SON [15]. Finding conserved or different *TERT* AS regulation in multiple cell and tissue types will result in the identification of therapeutic approaches by which specific manipulation of telomerase activity can be achieved in certain cell types (e.g., cancer cells versus stem cells). For example, we recently published that knockdown of SF3B4 induces reduction of FL *TERT* in NSCLC cells resulting in decreased telomerase activity, cell viability, and proliferation of cancer cells [40]. Conversely, when SF3B4 was knocked down in non-cancerous cells (human bronchial epithelial cells, HBECs) it did not reduce viability or proliferation of cells proposing a new approach for cancer cell specific therapy.

These data point out that *TERT* AS is regulated by both tissue specific and general RNA binding proteins. By prediction and or empirical identification of where these RNA binding proteins interact with their motifs on the *TERT* pre-mRNAs, we might be able to develop antisense oligonucleotide (ASO) type drugs to block the binding of the RNA binding proteins to switch the splicing of *TERT* from telomerase coding TERT to degraded or dominant-negative type TERTs. Indeed, research has already pointed out that SRSF2 binding is predicted at the intron 6/exon 7 boundary and groups have targeted this region using ASOs to induce minus beta splicing [44]. Further, we and others have utilized the binding motif of NOVA1 in direct repeat 8 to develop ASOs as well [11, 16, 45]. As we continue to elucidate the RNA binding proteins of *TERT* we will identify additional and potentially more potent motifs to target with splicing switching ASOs aimed at reducing telomerase activity for anticancer purposes. A provocative idea would be to target FL *TERT* repressor motifs with ASOs to promote FL *TERT* splicing and increase telomerase activity for regenerative medicine purposes.

Our data is not without limitations. Since we only tested a single stem cell line and a single cancer cell line, our data should therefore be interpreted with caution, and further research including additional cell lines should be performed to see if our predictions and models hold. Moreover, although we measured multiple *TERT* splice variants in this study, we did not measure all known *TERT* splice variants. To date, 21 *TERT* splice variants have been identified [13] and determining expression levels of all different splice variants is extremely challenging. Measurement of other known *TERT* splice variants or novel splice variants in different cell types will provide insights to fully understand *TERT* AS regulation. Another limitation of this study is that we did not perform the siRNA loss-of-function studies in the stem cells. Since our laboratory is mainly focused on inhibition of telomerase activity as a cancer therapeutic approach, we focused on dysregulated telomerase in this research. For future research, identification of the *TERT* splicing code should be performed as well as functional outcomes (telomerase activity, telomere length, cell survival, etc.) in different cell types that are characterized by regulated (i.e., stem cells) or dysregulated (i.e., cancer cells) alternative splicing.

Overall, our data represent an advance in our understanding of *TERT* regulation in stem cells and cancer cells. We utilized a novel model to investigate *TERT* splicing regulation in stem cells and contrasted this with associations of SFs in cancer cells. Our data revealed that certain SFs are dysregulated in cancer and do not seem to play a role in *TERT* regulation in stem cells. These data and subsequent studies may reveal a splicing factor(s) or their binding site(s) that could be targeted with small molecule drugs or antisense oligonucleotides, respectively, to reduce telomerase activity in cancer cells and promote durable cancer remissions.

## Supporting information

S1 Fig A) Representative phase contrast and fluorescent microscopy images support iPSC differentiation into NPC. B-E) mRNA expression levels of stem cell pluripotency markers (B,C) and NPC markers (D,E) support iPSC differentiation into NPC (determined by ddPCR; n = 3 biological replicates per condition). F) mRNA expression level of potential FL *TERT* (exons 7/8 inclusion) and minus beta (exons 7/8 exclusion) were measured in differentiation (determined by ddPCR; n = 3 biological replicates per condition). G) Reduction of telomerase activity in differentiation (determined by ddTRAP; n = 3 biological replicates per condition). H) Del 2 *TERT* (exon 2 exclusion) splice variant expression during differentiation increased compared to iPSCs (day 0) (determined by ddPCR; n = 3 biological replicates per condition). I-K) NOVA1 (I), PTBP1 (J), and PTBP2 (K) protein expression levels normalized by H3 protein expression (determined by western blot; n = 3 biological replicates per condition). L and M) Average *TERT* gene expression levels determined by ddPCR (n = 6 biological replicates per condition) in siRNA treated iPSC. Exons 7/8 including TERT (potential FL; L) and exons 7/8 excluding *TERT* (minus beta; M) splice variants were measured. One-way ANOVA with uncorrected Fisher’s LSD for post hoc comparisons were used to compare Day 10 and Day 29 with Day 0 (B-E, H; *, *P* < 0.05; **, *P* < 0.01; ***, *P* < 0.001; ****, *P* < 0.0001). For F and G, only One-way ANOVA was performed on the number of total TERT transcripts including/excluding exons 7/8 (F) and telomerase activity (G). One-way ANOVA with uncorrected Fisher’s LSD for post hoc comparisons were used to compare siRNA-treated conditions with siControl (siCTL; I-M; *, *P* < 0.05; **, *P* < 0.01; ***, *P* < 0.001; ****, *P* < 0.0001). Data are presented as means ± standard deviations where applicable.
(PDF)

S2 Fig A-G) Average *TERT* splice variant expression levels determined by ddPCR. minus alpha (A), INS3 (B), INS4 (C), minus gamma (D), plus gamma (E), intron 11 retention (F), and intron 14 retention (G) transcripts were quantified (determined by ddPCR; n = 4 biological replicates per condition). H) Pearson correlation analysis shows that changes of telomerase activity and ratio of exons 7/8 inclusion (potential FL) by iPSC cell density are positively and significantly correlated. 95% Confidence bands, Pearson’s correlation coefficient (*r*) and *p* value are shown. One-way ANOVA was performed to compare total amount of *TERT* splice variants from all conditions, but none of them had significantly different expression (A-G). For correlation analysis, 24 data points are included (H; six conditions x four replicates). Data are presented as means ± standard deviations where applicable.
(PDF)

S3 Fig A-D) Log2-transformed (A,B, and D) or raw (C) RSEM values of SFs gene-expression levels (n = 58 matched patient samples). CDC40 (A), HNRNPH1 (B), HNRNPCL1 (C), and HNRNPM (D) are not significantly differentially expressed in tumor tissue from LUAD patients. HNRNPCL1 was detected in only one sample out of 116 samples (C; 58 tumors and 58 normal tissue). Student t test set at *P* ≤ 0.05 for significance compared with normal tissue controls (all *P* > 0.05). In the box plots, the lower boundary of the box indicates the 25 th percentile, a line within the box marks the median and the higher boundary of the box indicates the 75 th percentile. Whiskers above and below the box indicate the 10 th and 90 th percentiles. Points above and below the whiskers indicate outliers outside the 10 th and 90 th percentiles.
(PDF)

S4 Fig A-C and E-G) Western blot of splicing factors and correlation analyses in different iPSC density. Top are representative images and bottoms are scatter plots showing correlation. 95% Confidence bands, Pearson’s correlation coefficient (*r*) and *p* value are shown. Antibodies targeting HNRNPA1 (A), U2AF2 (B), CDC40 (C), beta actin or GAPDH (D; loading control), PTBP1 (E), NOVA1 (F), and PTBP2 (G) were used for western blot (n = 4 for A-D and n = 3 for E-G biological replicates per condition). Pearson’s linear correlational analysis was performed between splicing factors and *TERT* exons 7/8 inclusion expression percentage of total TERT. D) Western blot of beta actin and GAPDH used for normalization of target genes. Bottom panel shows quantifications of beta actin and GAPDH normalized by average of six conditions. Statistical significance was not found by one-way analysis of variance (ANOVA) comparing all conditions (D; *P* = 0.78). Data are presented as means ± standard deviations where applicable. For correlation analysis, 24 data points (A-C; six conditions x four replicates) or 18 data points (E-G; six conditions x three replicates) are included.
(PDF)

S5 Fig A-B) the expression of *TERT* transcripts with exons 7/8 (potential FL; A) and without exons 7/8 (minus beta; B) were measured after knockdown using siRNAs (determined by ddPCR; n = 6 biological replicates per condition). C) Telomerase activity was reduced by all siRNA treatment (determined by ddTRAP; n = 6 biological replicates per condition). D-F) Western blot of HNRNPM (D), SRSF2 (E), and U2AF2 (F) and protein expression quantifications normalized by beta actin. For knockdown experiments, one-way ANOVA with uncorrected Fisher’s LSD for post hoc comparisons of siRNA treatments were used to compare siRNA-treated conditions with siControl (A-C; **, *P* < 0.01; ***, *P* < 0.001; ****, *P* < 0.0001). For comparisons of splicing factor expression, Student t test was used to determine statistical significance (D-F; ns, *P* > 0.5; ***, *P* < 0.001). Data are presented as means ± standard deviations where applicable.
(PDF)

S1 Table Summary of SFs from three different approaches. Green color indicates potential FL TERT promoters, and red color indicates potential minus beta promoters.
(PDF)

S1 File Validation of primers targeting minus and plus gamma *TERT*. (PDF)

S1 Raw images (PDF)
