# Supplementary figures and images for: Dynamics of TERT regulation via alternative splicing in stem cells and cancer cells
Source: PLoS One. 2023 Aug 2;18(8):e0289327. doi: 10.1371/journal.pone.0289327 (PMC10395990; doi:10.1371/journal.pone.0289327)

# S1 Fig.

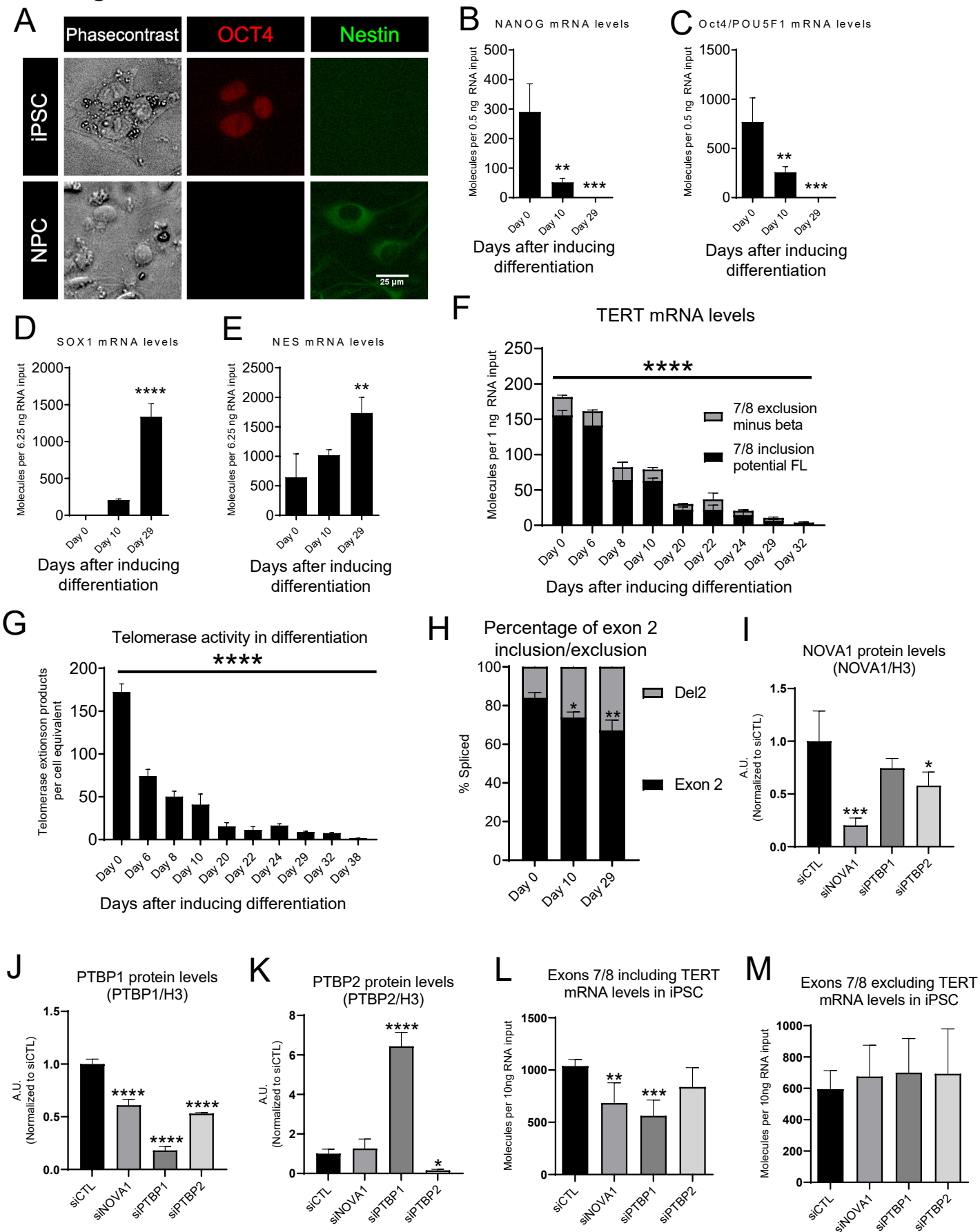

Supplement: S1 Fig — A) Representative phase contrast and fluorescent microscopy images support iPSC differentiation into NPC. B-E) mRNA expression levels of stem cell pluripotency markers (B,C) and NPC markers (D,E) support iPSC differentiation into NPC (determined by ddPCR; n = 3 biological replicates per condition). F) mRNA expression level of potential FL TERT (exons 7/8 inclusion) and minus beta (exons 7/8 exclusion) were measured in differentiation (determined by ddPCR; n = 3 biological replicates per condition). G) Reduction of telomerase activity in differentiation (determined by ddTRAP; n = 3 biological replicates per condition). H) Del 2 TERT (exon 2 exclusion) splice variant expression during differentiation increased compared to iPSCs (day 0) (determined by ddPCR; n = 3 biological replicates per condition). I-K) NOVA1 (I), PTBP1 (J), and PTBP2 (K) protein expression levels normalized by H3 protein expression (determined by western blot; n = 3 biological replicates per condition). L and M) Average TERT gene expression levels determined by ddPCR (n = 6 biological replicates per condition) in siRNA treated iPSC. Exons 7/8 including TERT (potential FL; L) and exons 7/8 excluding TERT (minus beta; M) splice variants were measured. One-way ANOVA with uncorrected Fisher’s LSD for post hoc comparisons were used to compare Day 10 and Day 29 with Day 0 (B-E, H; *, P < 0.05; **, P < 0.01; ***, P < 0.001; ****, P < 0.0001). For F and G, only One-way ANOVA was performed on the number of total TERT transcripts including/excluding exons 7/8 (F) and telomerase activity (G). One-way ANOVA with uncorrected Fisher’s LSD for post hoc comparisons were used to compare siRNA-treated conditions with siControl (siCTL; I-M; *, P < 0.05; **, P < 0.01; ***, P < 0.001; ****, P < 0.0001). Data are presented as means ± standard deviations where applicable. (PDF) [file pone.0289327.s001.pdf]

S2 Fig.

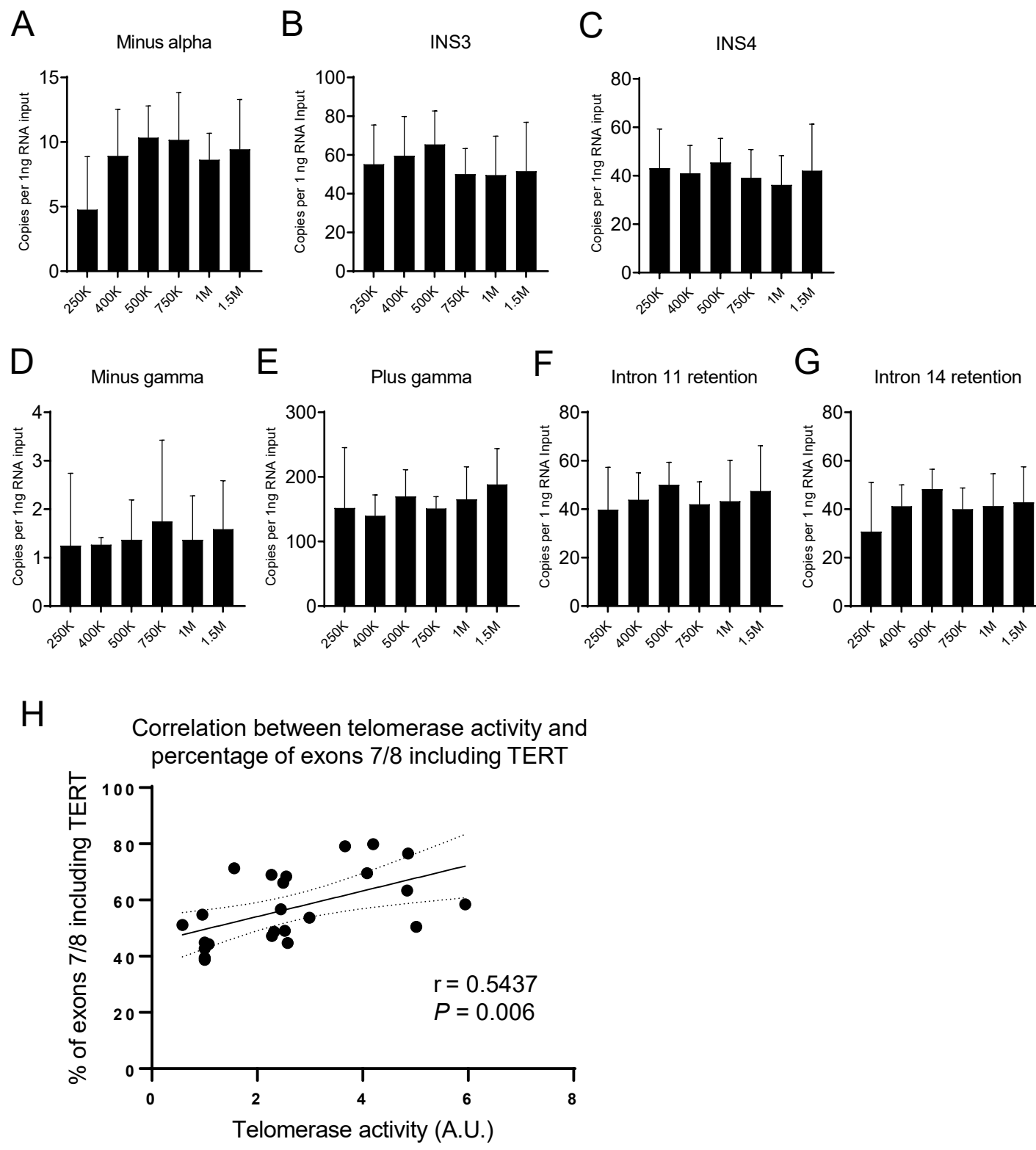

Supplement: S2 Fig — A-G) Average TERT splice variant expression levels determined by ddPCR. minus alpha (A), INS3 (B), INS4 (C), minus gamma (D), plus gamma (E), intron 11 retention (F), and intron 14 retention (G) transcripts were quantified (determined by ddPCR; n = 4 biological replicates per condition). H) Pearson correlation analysis shows that changes of telomerase activity and ratio of exons 7/8 inclusion (potential FL) by iPSC cell density are positively and significantly correlated. 95% Confidence bands, Pearson’s correlation coefficient (r) and p value are shown. One-way ANOVA was performed to compare total amount of TERT splice variants from all conditions, but none of them had significantly different expression (A-G). For correlation analysis, 24 data points are included (H; six conditions x four replicates). Data are presented as means ± standard deviations where applicable. (PDF) [file pone.0289327.s002.pdf]

S3 Fig.

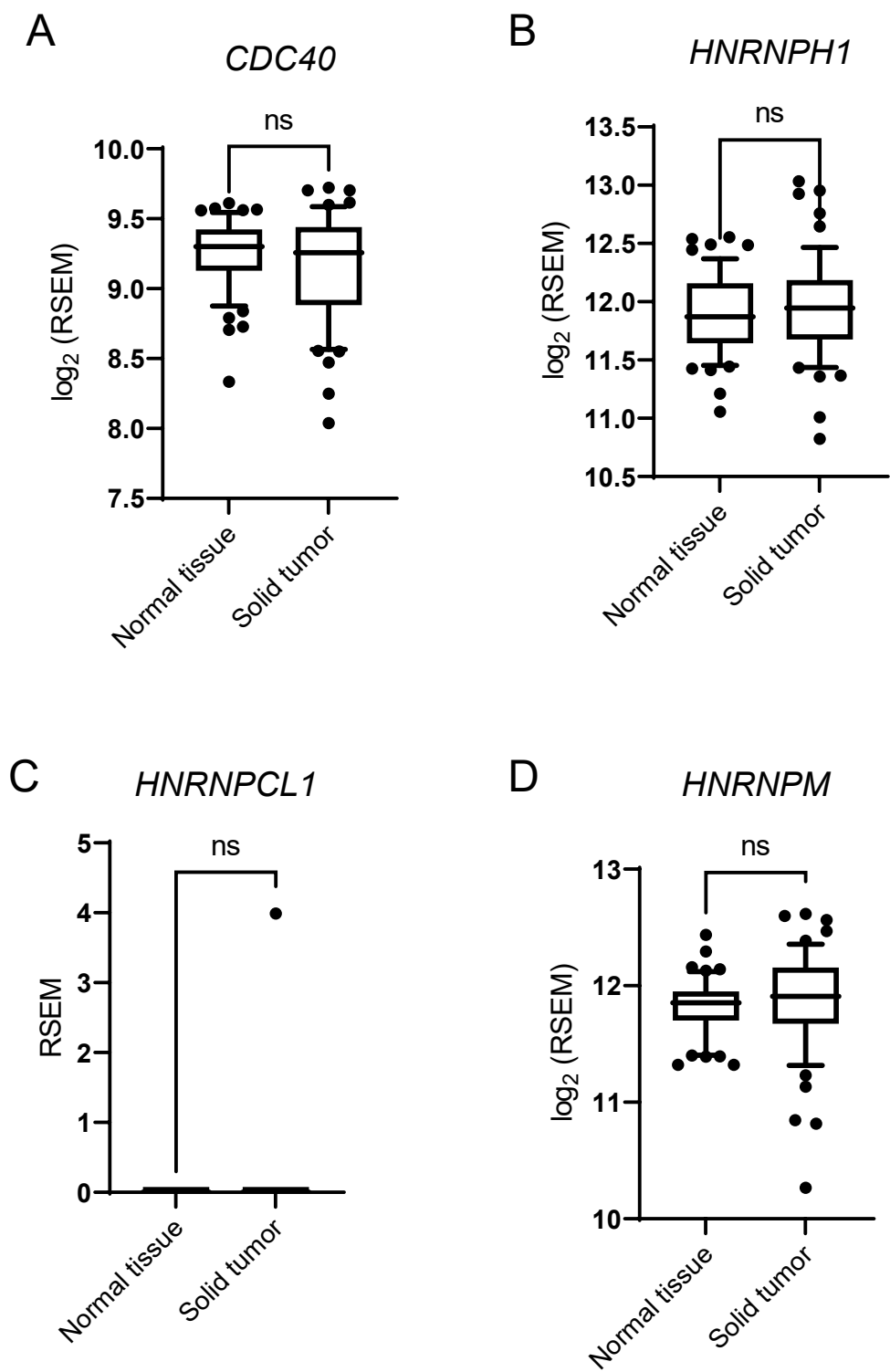

Supplement: S3 Fig — A-D) Log2-transformed (A,B, and D) or raw (C) RSEM values of SFs gene-expression levels (n = 58 matched patient samples). CDC40 (A), HNRNPH1 (B), HNRNPCL1 (C), and HNRNPM (D) are not significantly differentially expressed in tumor tissue from LUAD patients. HNRNPCL1 was detected in only one sample out of 116 samples (C; 58 tumors and 58 normal tissue). Student t test set at P ≤ 0.05 for significance compared with normal tissue controls (all P > 0.05). In the box plots, the lower boundary of the box indicates the 25 th percentile, a line within the box marks the median and the higher boundary of the box indicates the 75 th percentile. Whiskers above and below the box indicate the 10 th and 90 th percentiles. Points above and below the whiskers indicate outliers outside the 10 th and 90 th percentiles. (PDF) [file pone.0289327.s003.pdf]

# S4 Fig.

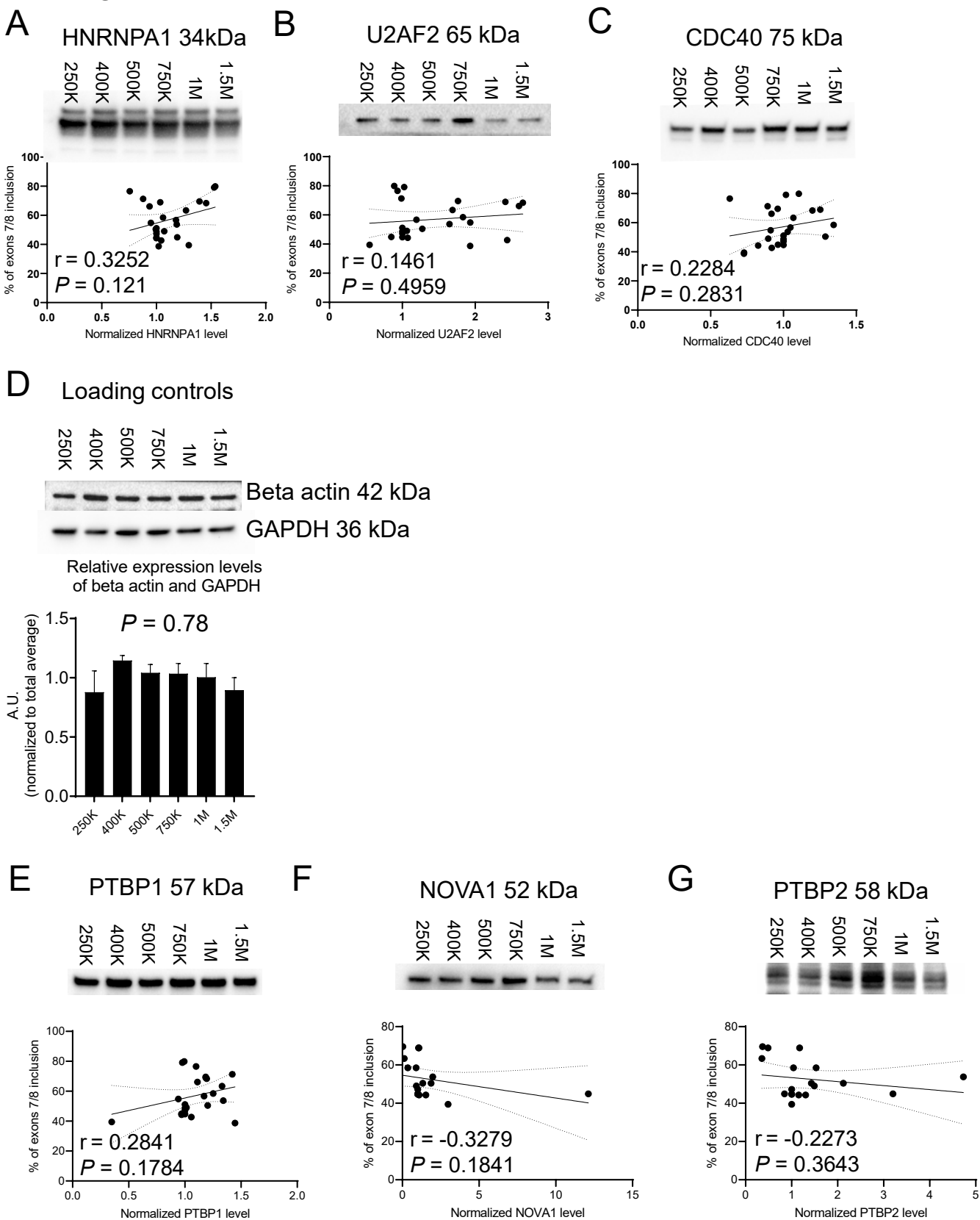

Supplement: S4 Fig — A-C and E-G) Western blot of splicing factors and correlation analyses in different iPSC density. Top are representative images and bottoms are scatter plots showing correlation. 95% Confidence bands, Pearson’s correlation coefficient (r) and p value are shown. Antibodies targeting HNRNPA1 (A), U2AF2 (B), CDC40 (C), beta actin or GAPDH (D; loading control), PTBP1 (E), NOVA1 (F), and PTBP2 (G) were used for western blot (n = 4 for A-D and n = 3 for E-G biological replicates per condition). Pearson’s linear correlational analysis was performed between splicing factors and TERT exons 7/8 inclusion expression percentage of total TERT. D) Western blot of beta actin and GAPDH used for normalization of target genes. Bottom panel shows quantifications of beta actin and GAPDH normalized by average of six conditions. Statistical significance was not found by one-way analysis of variance (ANOVA) comparing all conditions (D; P = 0.78). Data are presented as means ± standard deviations where applicable. For correlation analysis, 24 data points (A-C; six conditions x four replicates) or 18 data points (E-G; six conditions x three replicates) are included. (PDF) [file pone.0289327.s004.pdf]

S5 Fig.

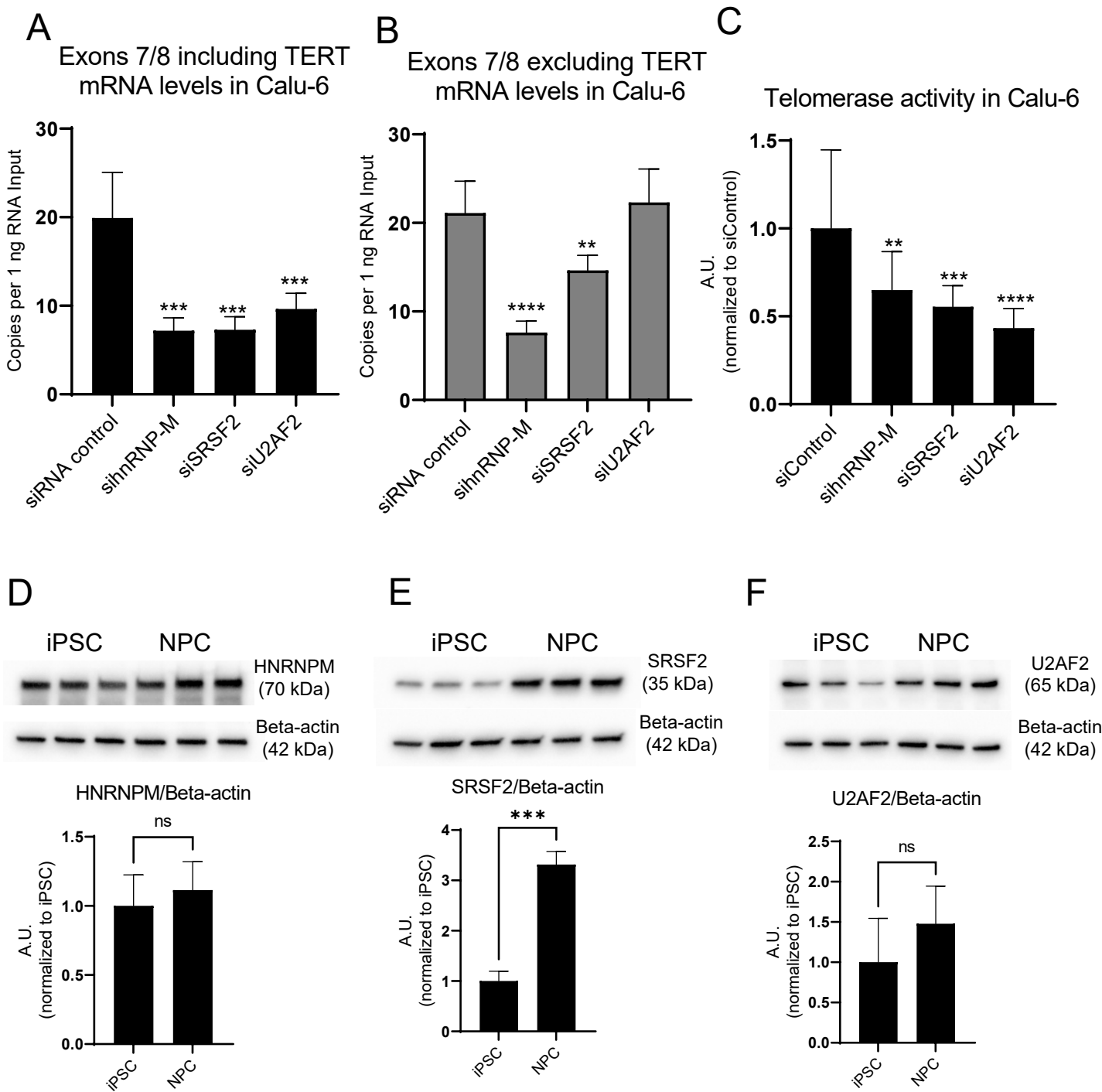

Supplement: S5 Fig — A-B) the expression of TERT transcripts with exons 7/8 (potential FL; A) and without exons 7/8 (minus beta; B) were measured after knockdown using siRNAs (determined by ddPCR; n = 6 biological replicates per condition). C) Telomerase activity was reduced by all siRNA treatment (determined by ddTRAP; n = 6 biological replicates per condition). D-F) Western blot of HNRNPM (D), SRSF2 (E), and U2AF2 (F) and protein expression quantifications normalized by beta actin. For knockdown experiments, one-way ANOVA with uncorrected Fisher’s LSD for post hoc comparisons of siRNA treatments were used to compare siRNA-treated conditions with siControl (A-C; **, P < 0.01; ***, P < 0.001; ****, P < 0.0001). For comparisons of splicing factor expression, Student t test was used to determine statistical significance (D-F; ns, P > 0.5; ***, P < 0.001). Data are presented as means ± standard deviations where applicable. (PDF) [file pone.0289327.s005.pdf]

Plus gamma  
Expected size: 180bp  
PCR: 30 cycles

Calu-6

NTC

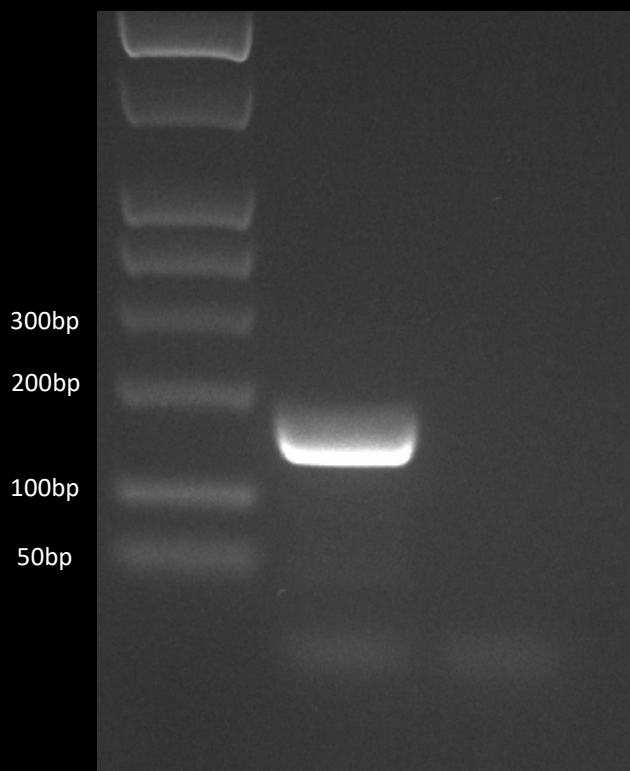

Minus gamma  
Expected size: 121 bp  
PCR: 40 cycles

Calu-6

NTC

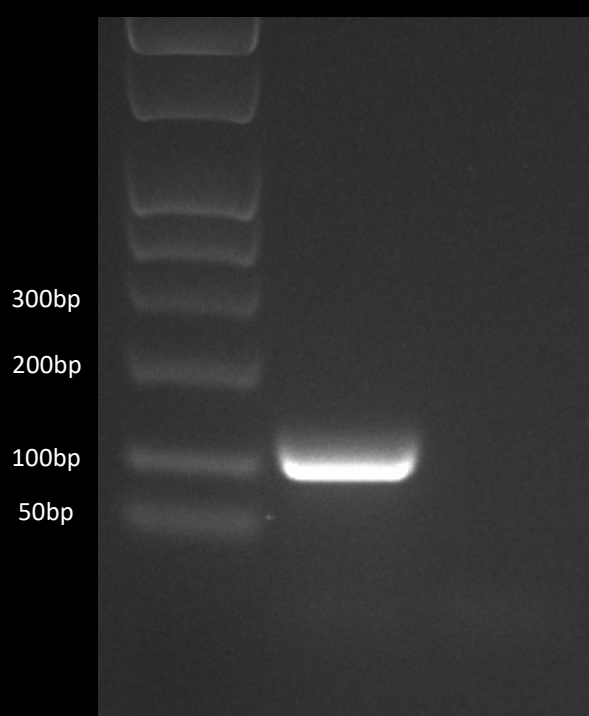

Supplement: S1 File — (PDF) [file pone.0289327.s007.pdf]
