# Supplementary material for: Dynamics of TERT regulation via alternative splicing in stem cells and cancer cells
Source: PLoS One. 2023 Aug 2;18(8):e0289327. doi: 10.1371/journal.pone.0289327 (PMC10395990; doi:10.1371/journal.pone.0289327)
Supplement: S1 Table — Green color indicates potential FL TERT promoters, and red color indicates potential minus beta promoters. (PDF) [file pone.0289327.s006.pdf]

Table S1. Summary of SFs from three different approaches

Green (SFs potentially promote FL)
SFs that have positive correlation with potential FL TERT in iPSC ($p < 0.05$)
SFs that increase exon 7/8 skipping more than 2-fold when KD in HeLa
SFs that are upregulated significantly in LUAD

Red (SFs potentially promote minus beta)
SFs that have negative correlation with potential FL TERT in iPSC ($p < 0.05$)
SFs that decrease exon 7/8 skipping more than 2-fold when KD in HeLa
SFs that are downregulated significantly in LUAD

Splicing Factors	iPSC density	Minigene in HeLa	LUAD patients data of TCGA	Type
hnRNP-CL1	P <0.05	x	NS	1
hnRNP-H1	P < 0.01	x	NS	1
SRPK1	P < 0.01	x	Up in LUAD	2
hnRNP-A2B1	P < 0.01	x	Up in LUAD	2
hnRNP-M	P < 0.01	Promote -beta	NS	3
SRSF2	P < 0.01	Promote FL	Up in LUAD	4
U2AF2	NS	Promote FL	Up in LUAD	5
hnRNP-A1	NS	Promote -beta	Up in LUAD	6
CDC40	NS	Promote FL	NS	7
