# Supplementary material for: Dynamics of TERT regulation via alternative splicing in stem cells and cancer cells
Source: PLoS One. 2023 Aug 2;18(8):e0289327. doi: 10.1371/journal.pone.0289327 (PMC10395990; doi:10.1371/journal.pone.0289327)

Figure 1D – TRF

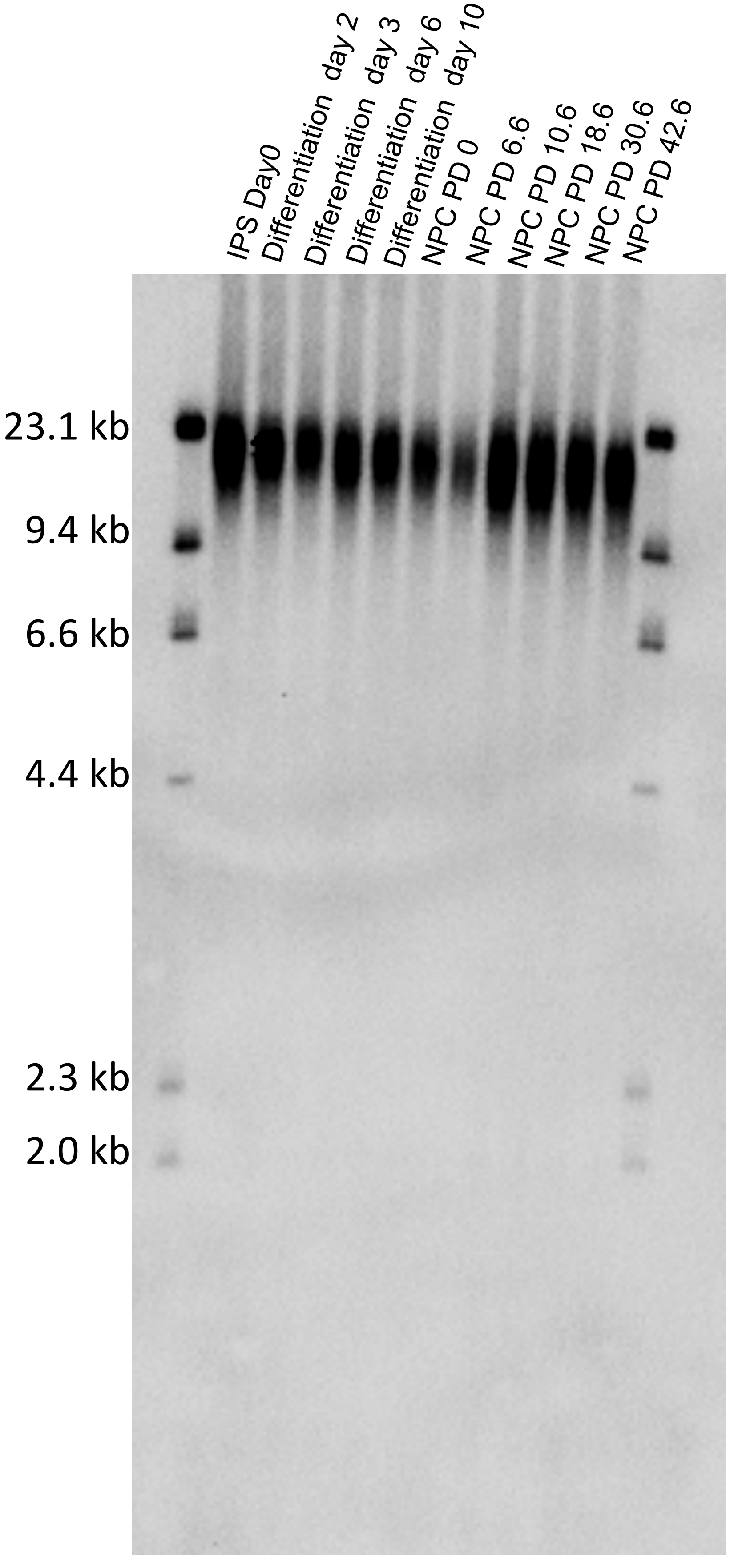

Figure 1A – Immunoblot – iPSC to NPC

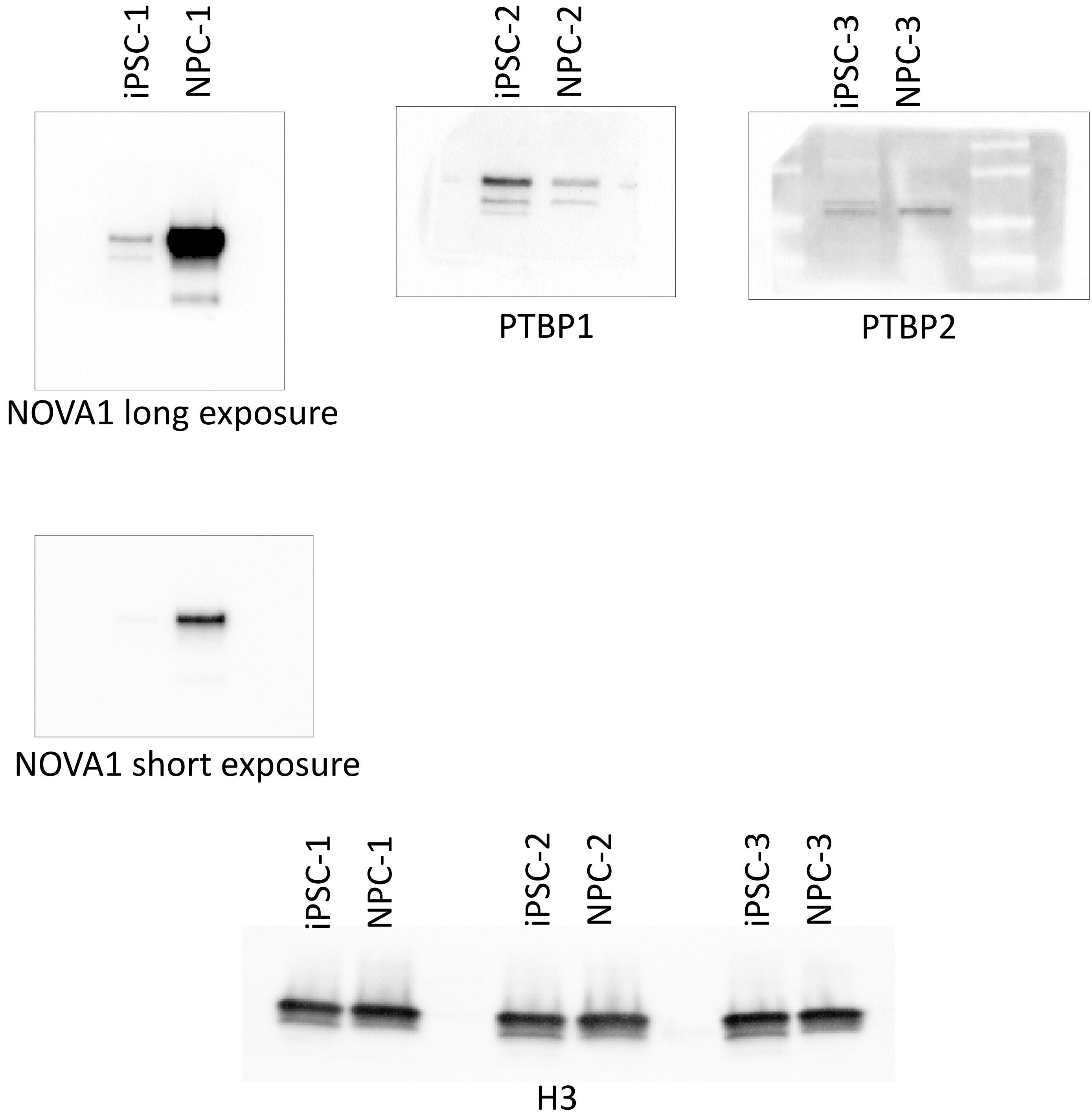

Figure 1E Immunoblot – siRNA treated iPSCs first repeat – used for representative image (Fig 1E) and quantification (S1I-K Figs)

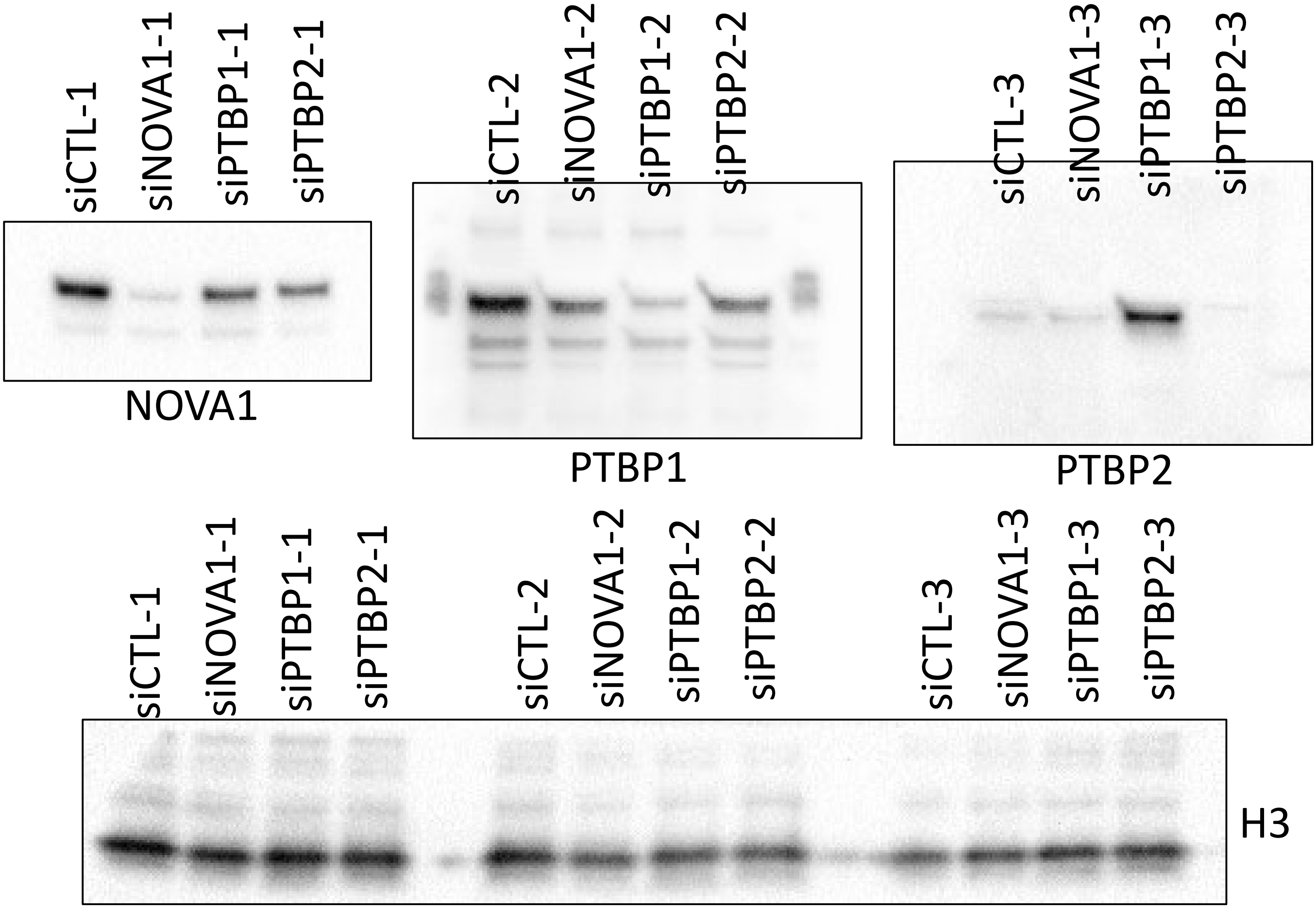

Blots merged to check size ladder (Dual Color Standards)

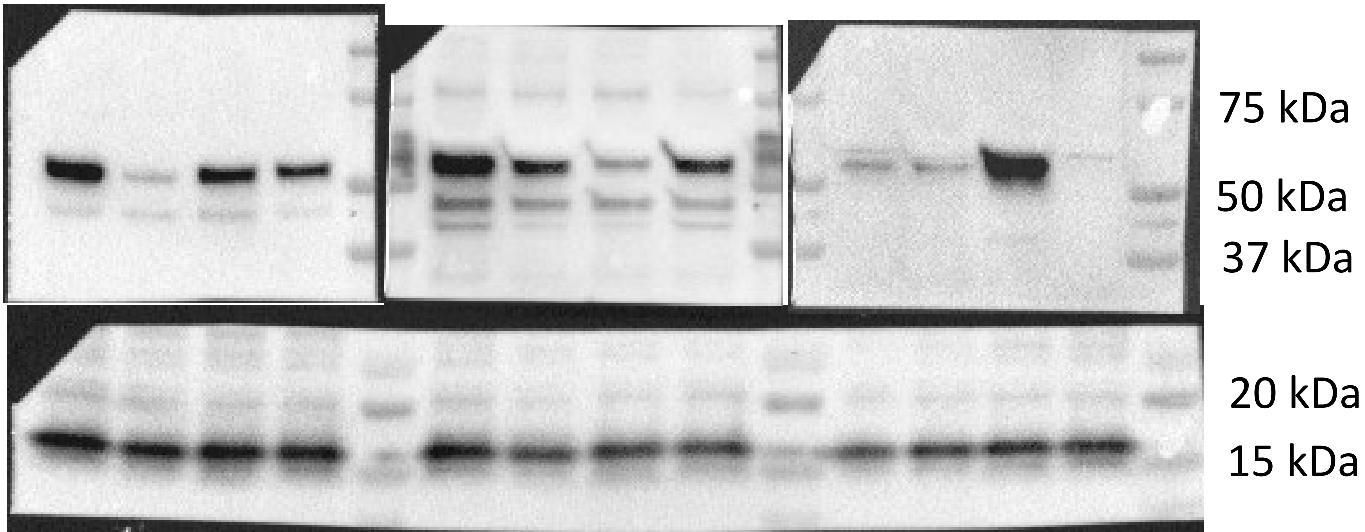

Figure 1E Immunoblot – siRNA treated iPSCs second repeat – used for quantification (S1I-K Figs)

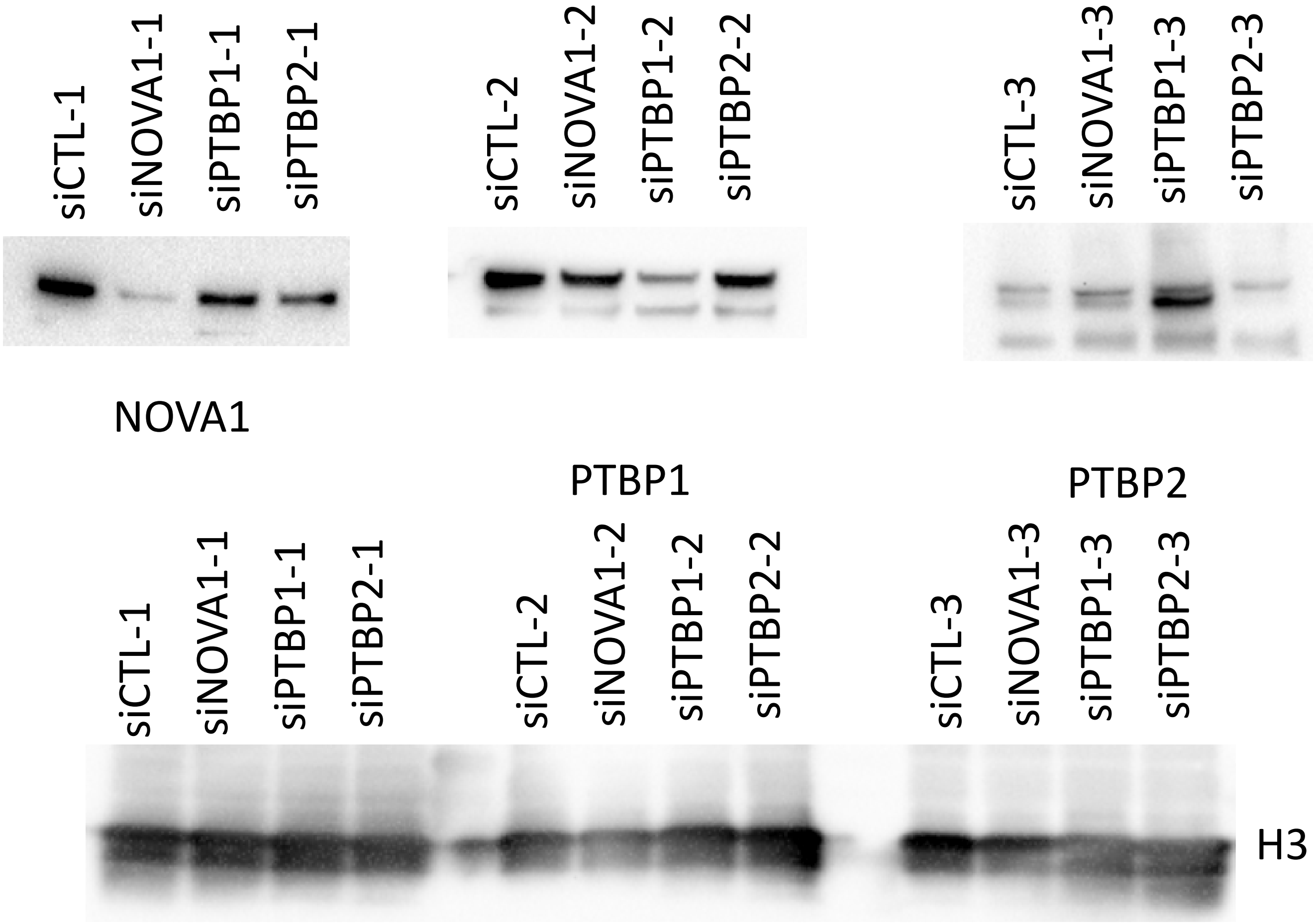

Blots merged to check size ladder (Dual Color Standards)

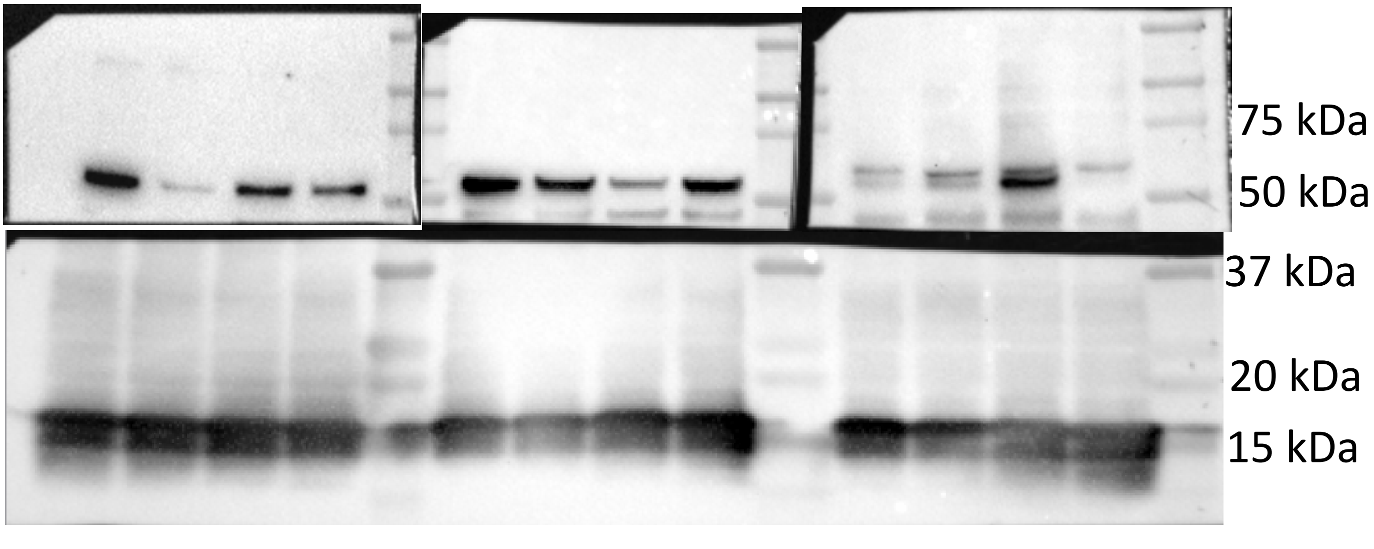

Figure 1E Immunoblot – siRNA treated iPSCs third repeat – used for quantification (S1I-K Figs)

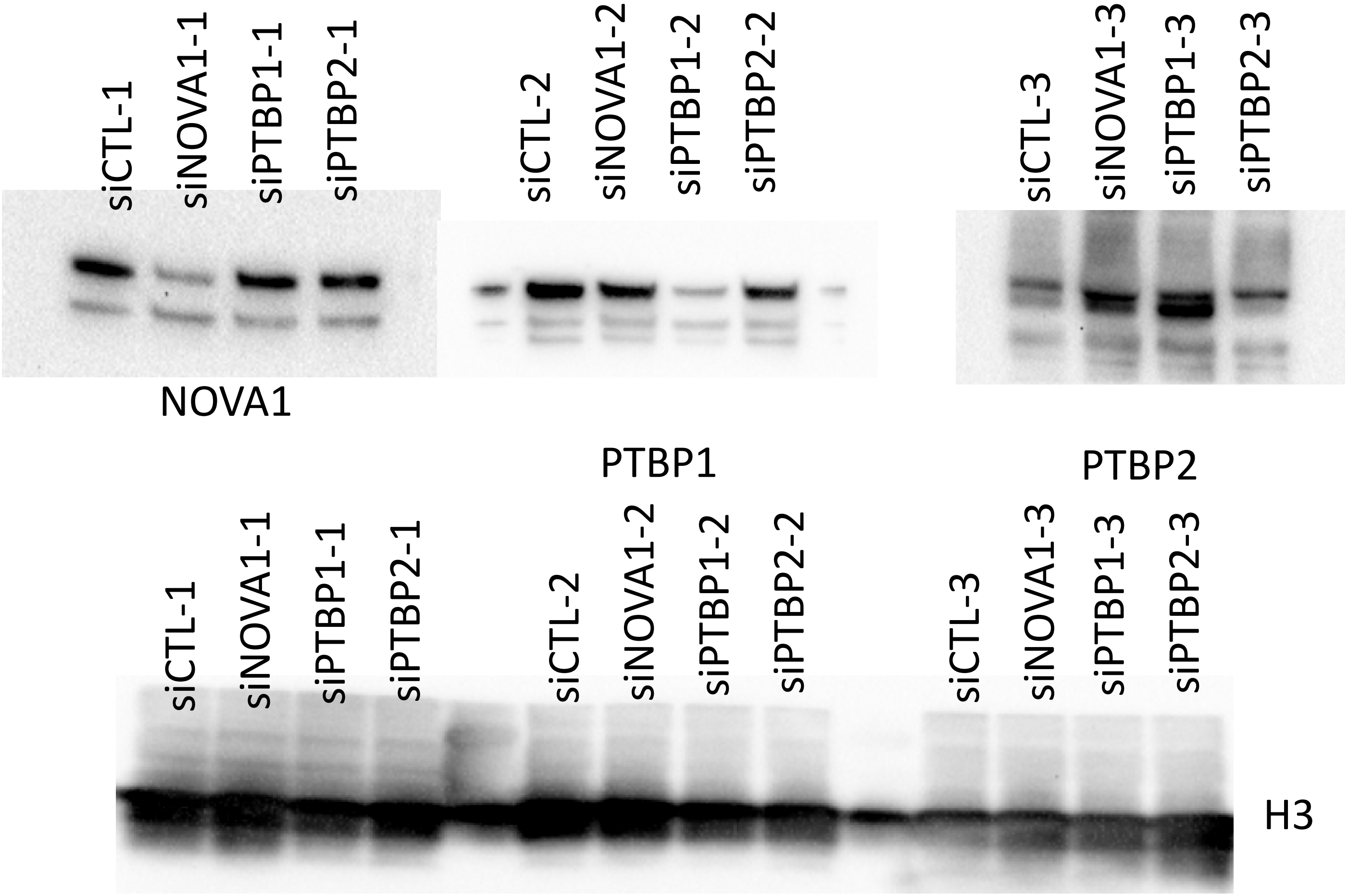

Blots merged to check size ladder (Dual Color Standards)

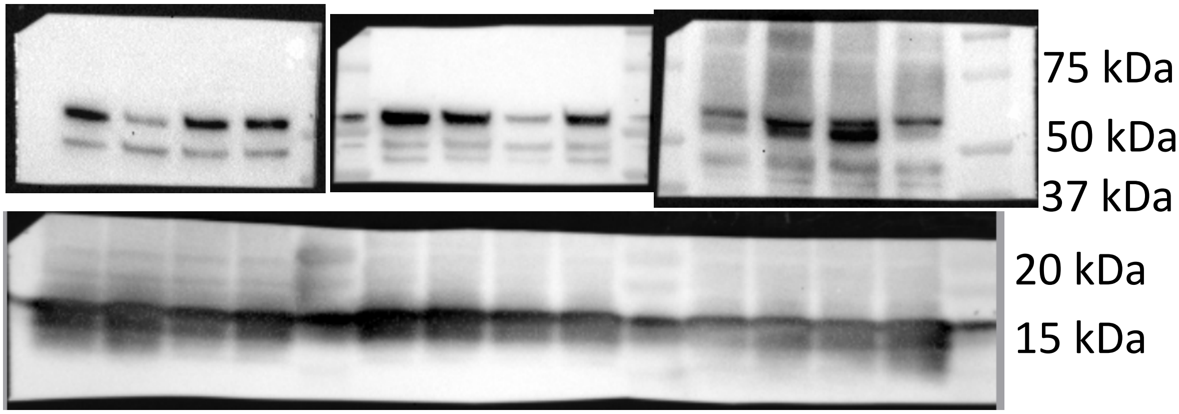

Figure 5 Immunoblot – splicing factors

6 Samples are loaded in the same order for each repeat
250K-400K-500K-750K-1M-1.5M

Only 250K and 1.5M are indicated to reduce complexity

Dual Color Standards

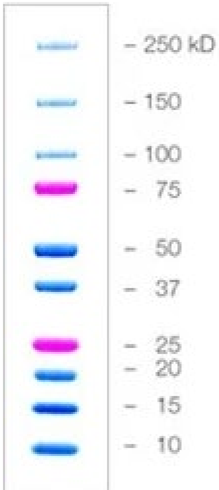

Figure 5 Immunoblot – splicing factors HNRNPA2B1

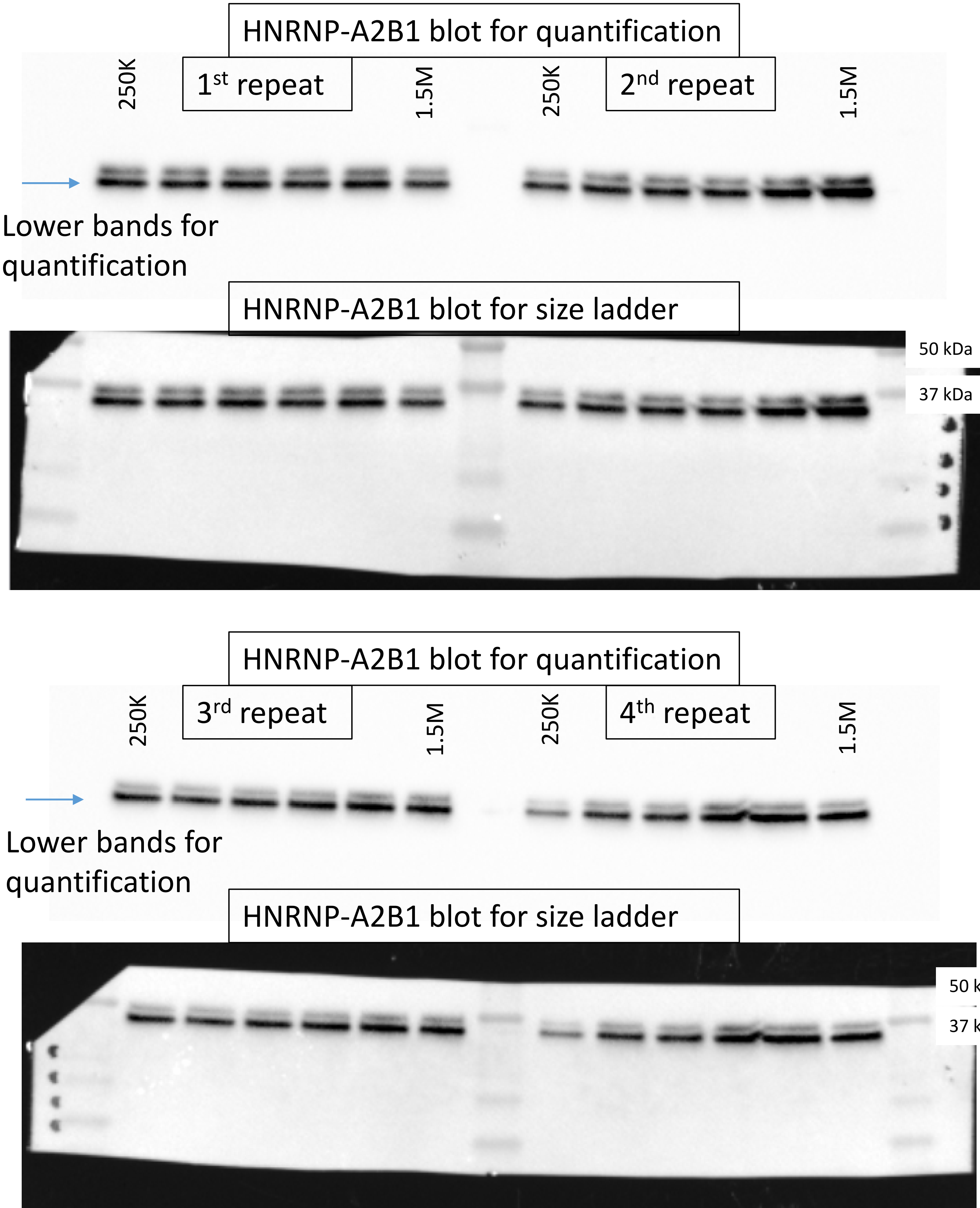

Figure 5 Immunoblot – splicing factors HNRNPCL1

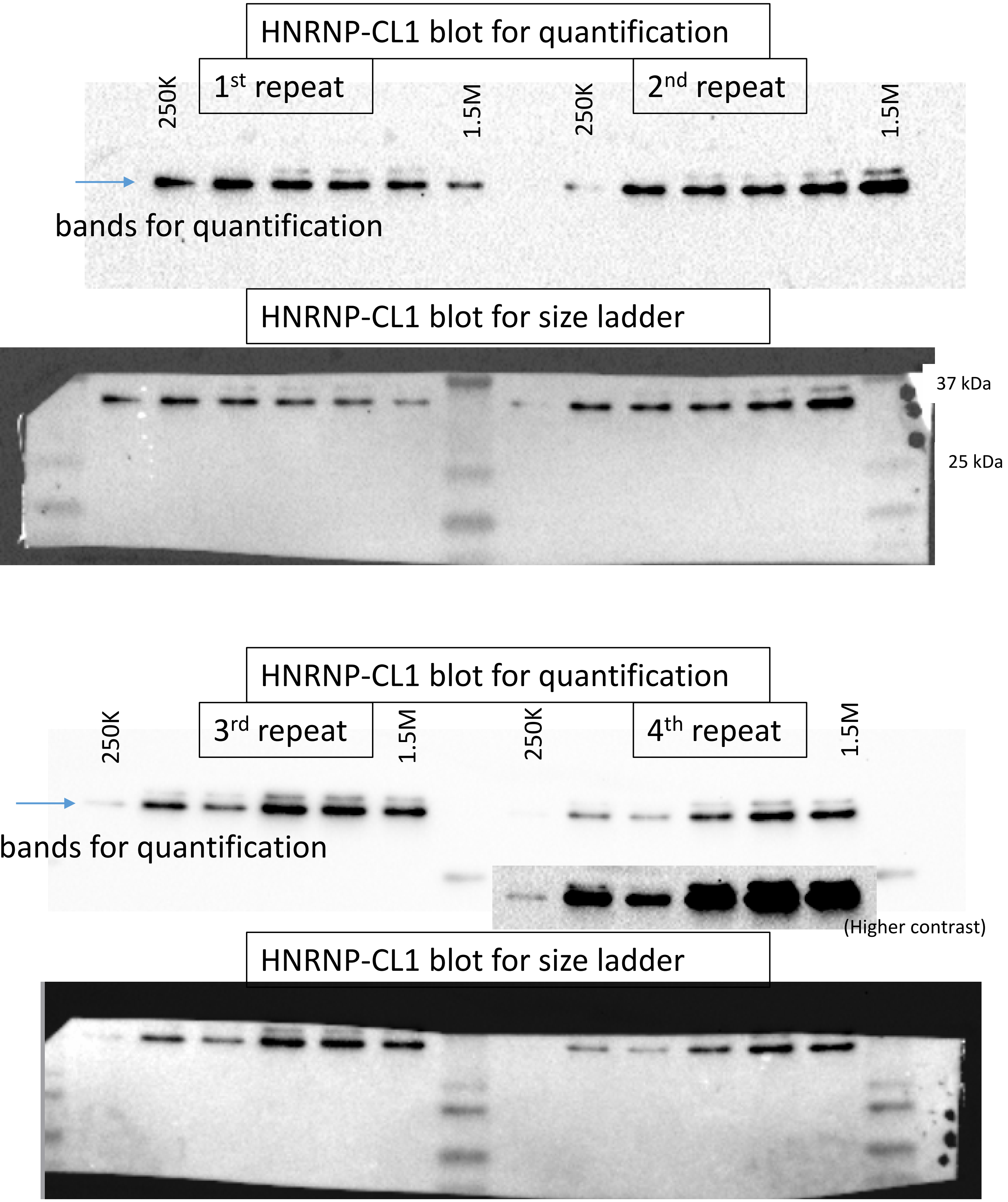

Figure 5 Immunoblot – splicing factors HNRNPH

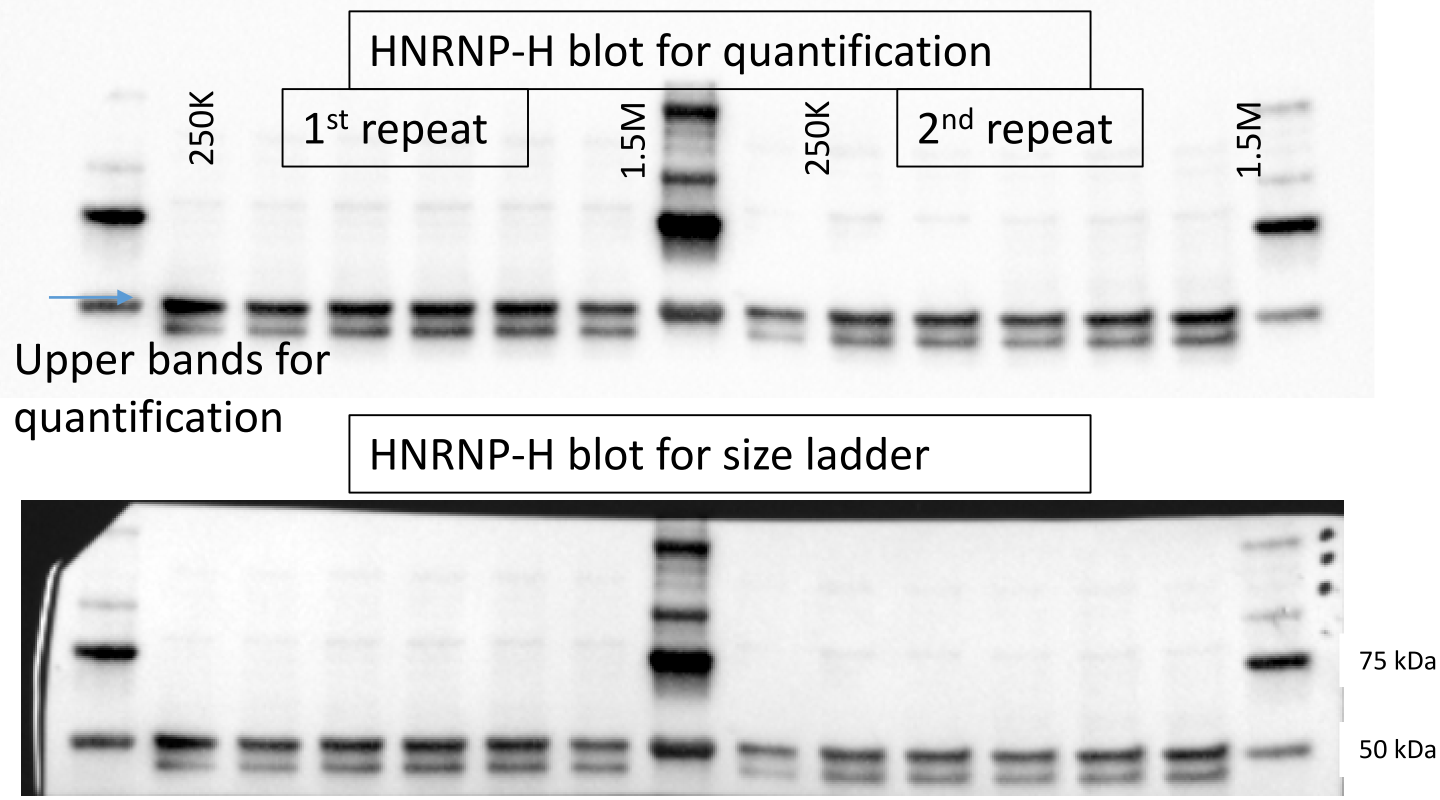

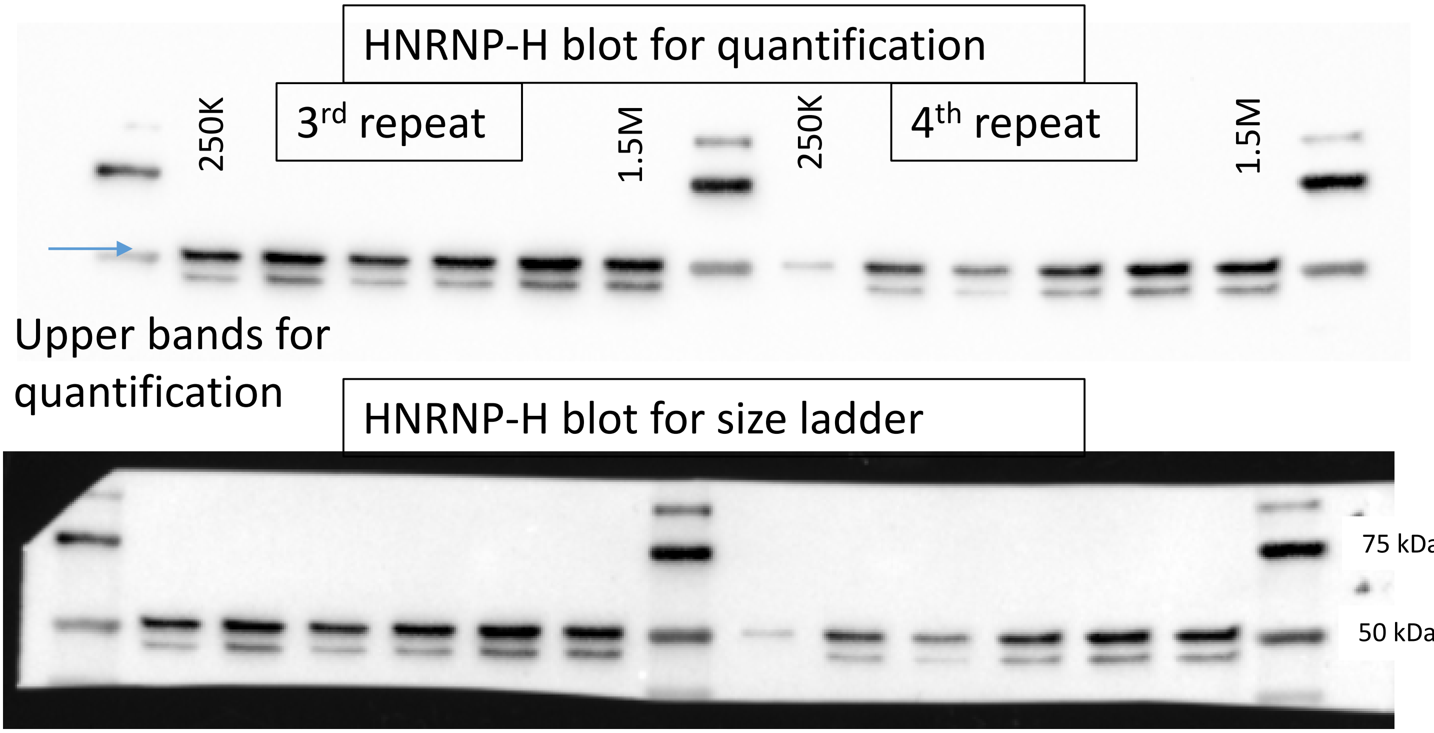

Figure 5 Immunoblot – splicing factors HNRNPM

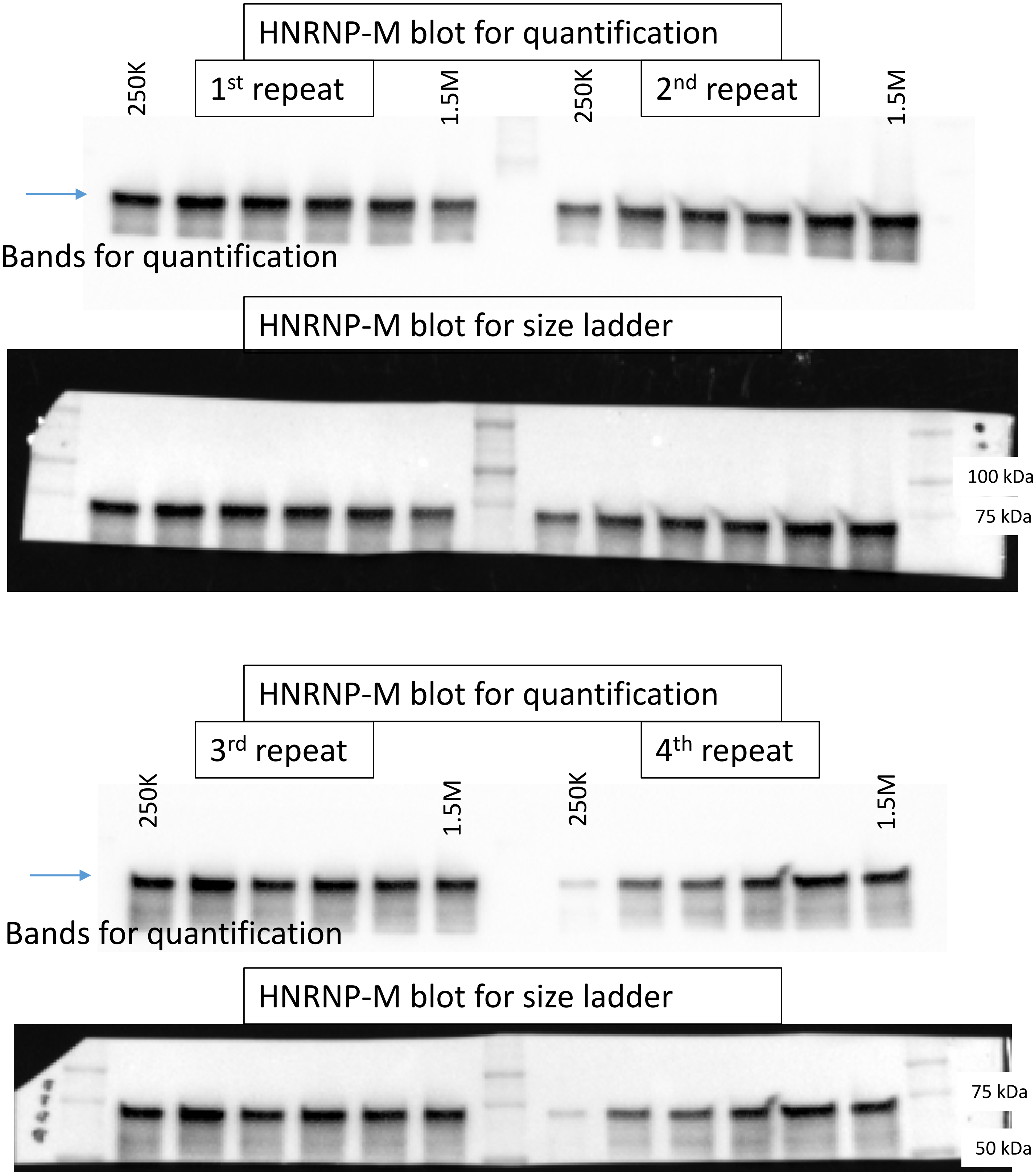

Figure 5 Immunoblot – splicing factors SRSF2

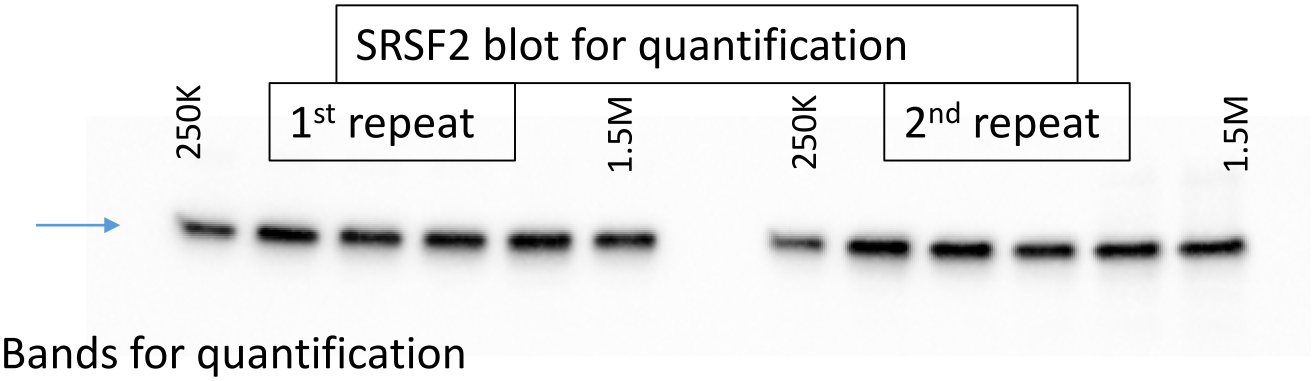

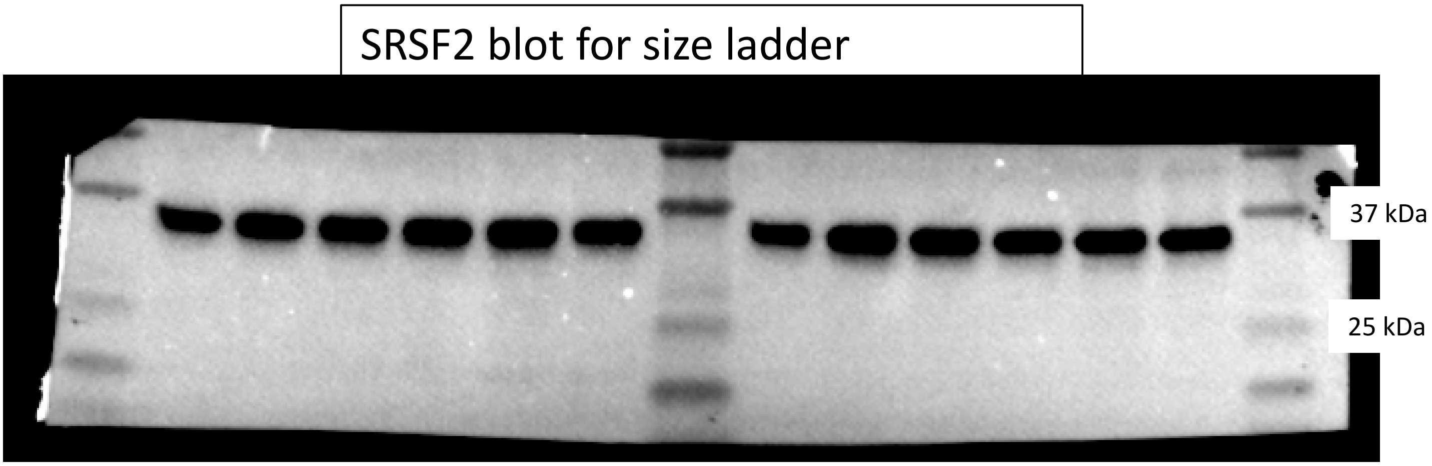

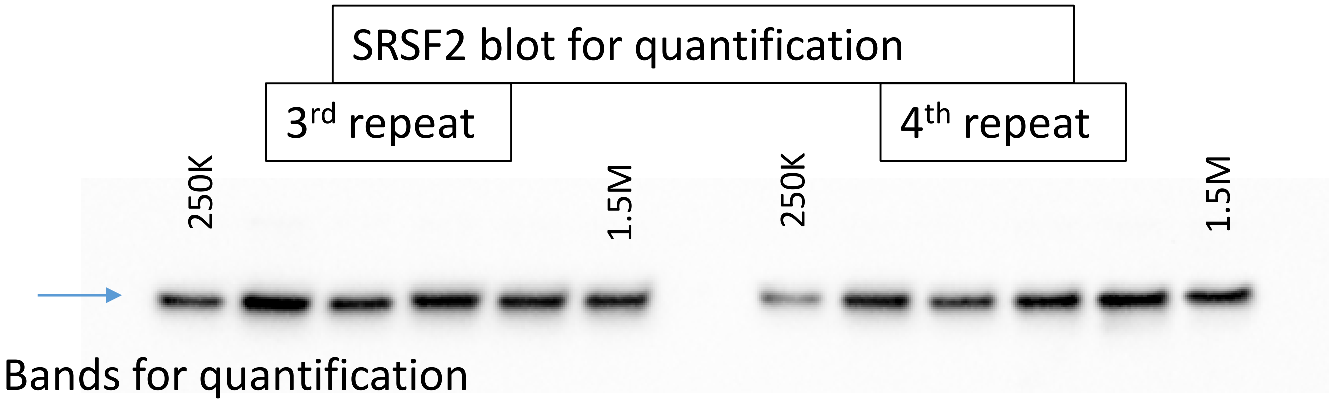

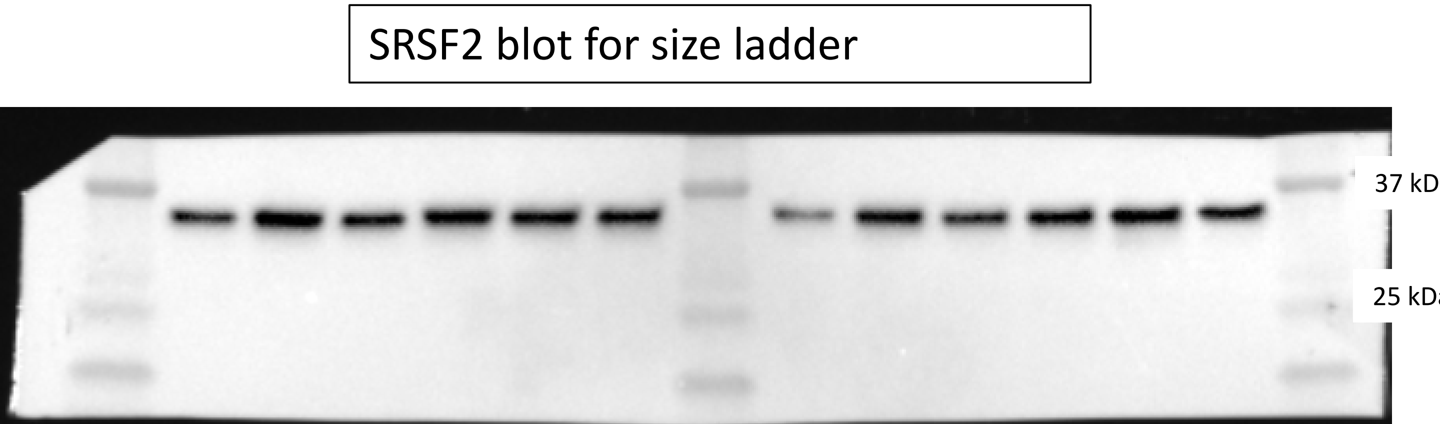

Figure 5 Immunoblot – splicing factors
SRPK1

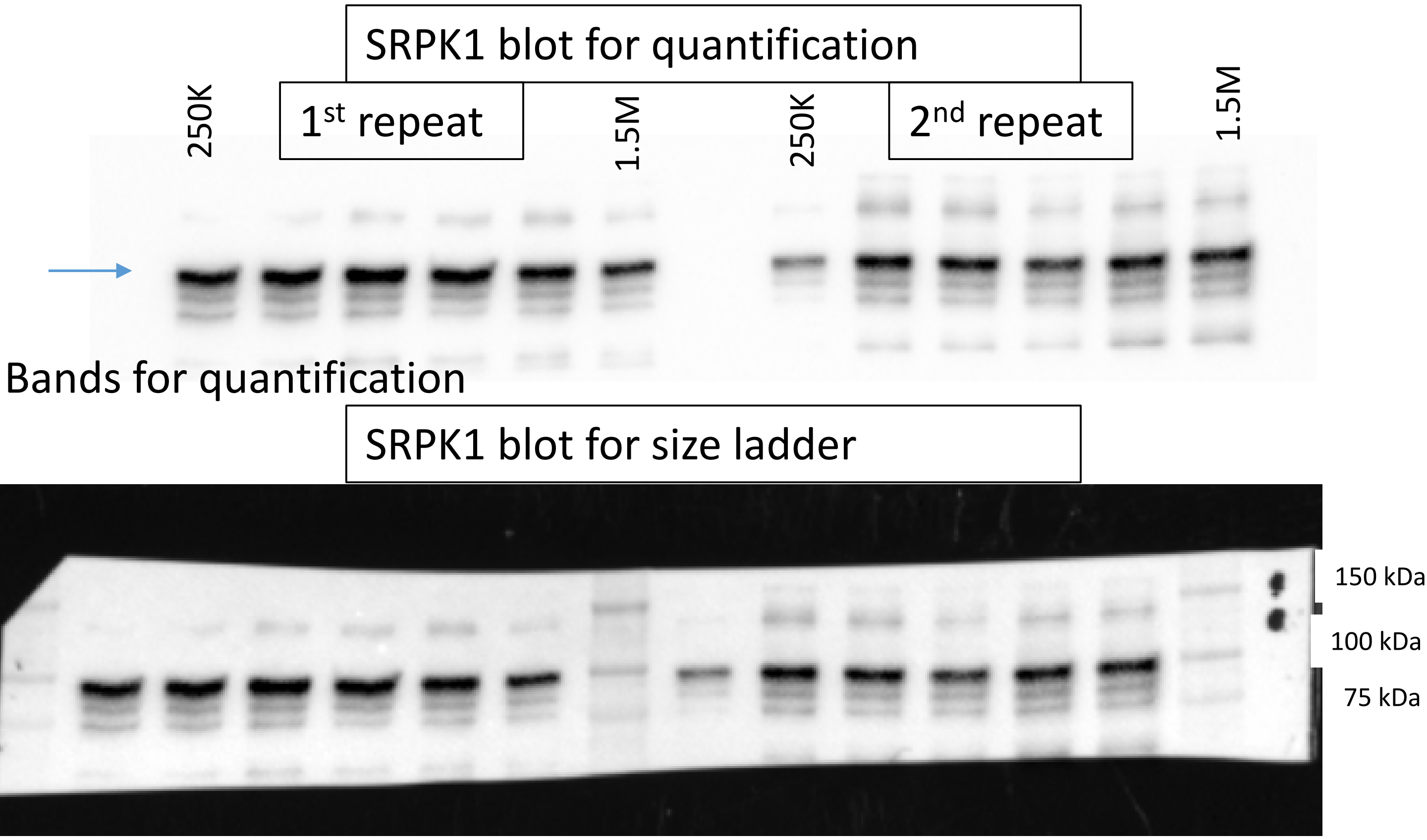

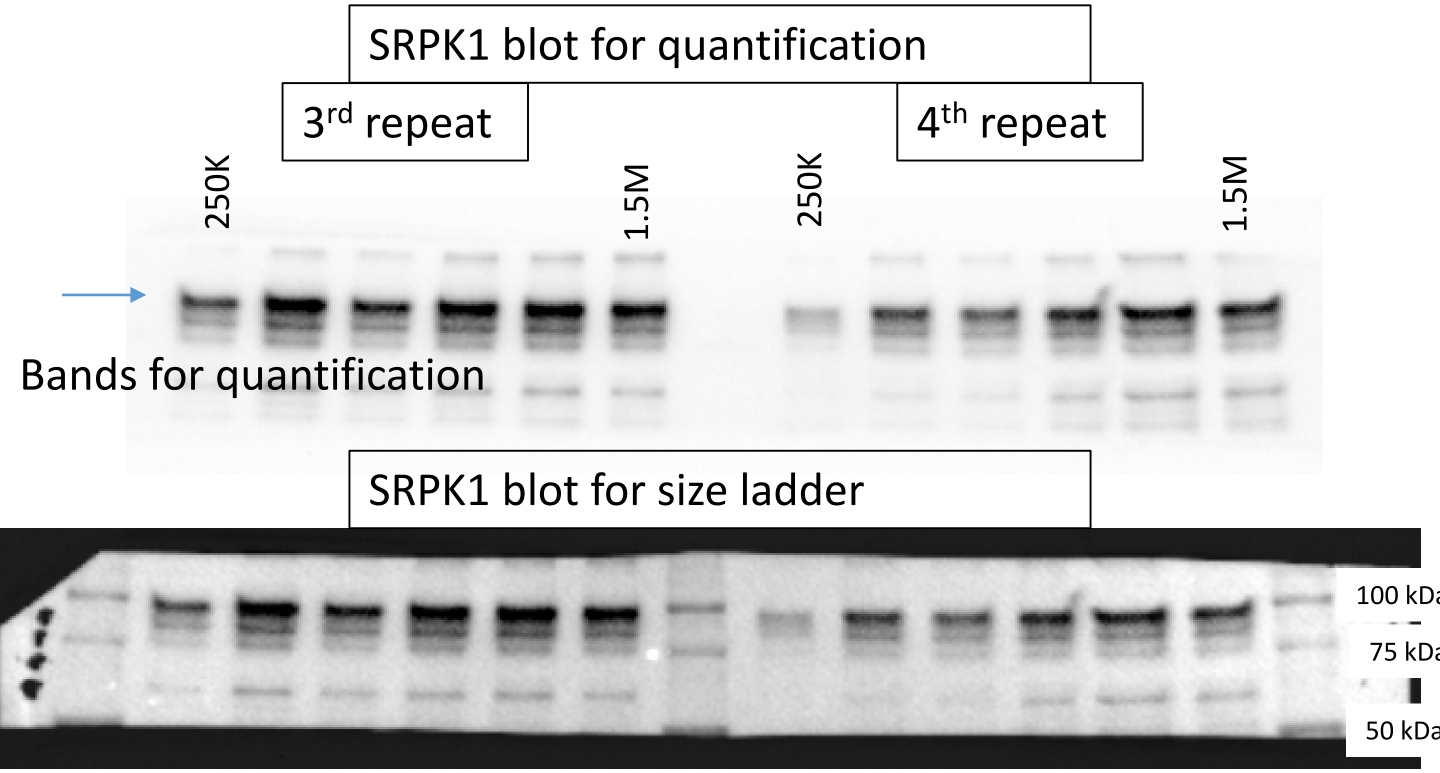

Figure 5 Immunoblot – splicing factors HNRNPA1

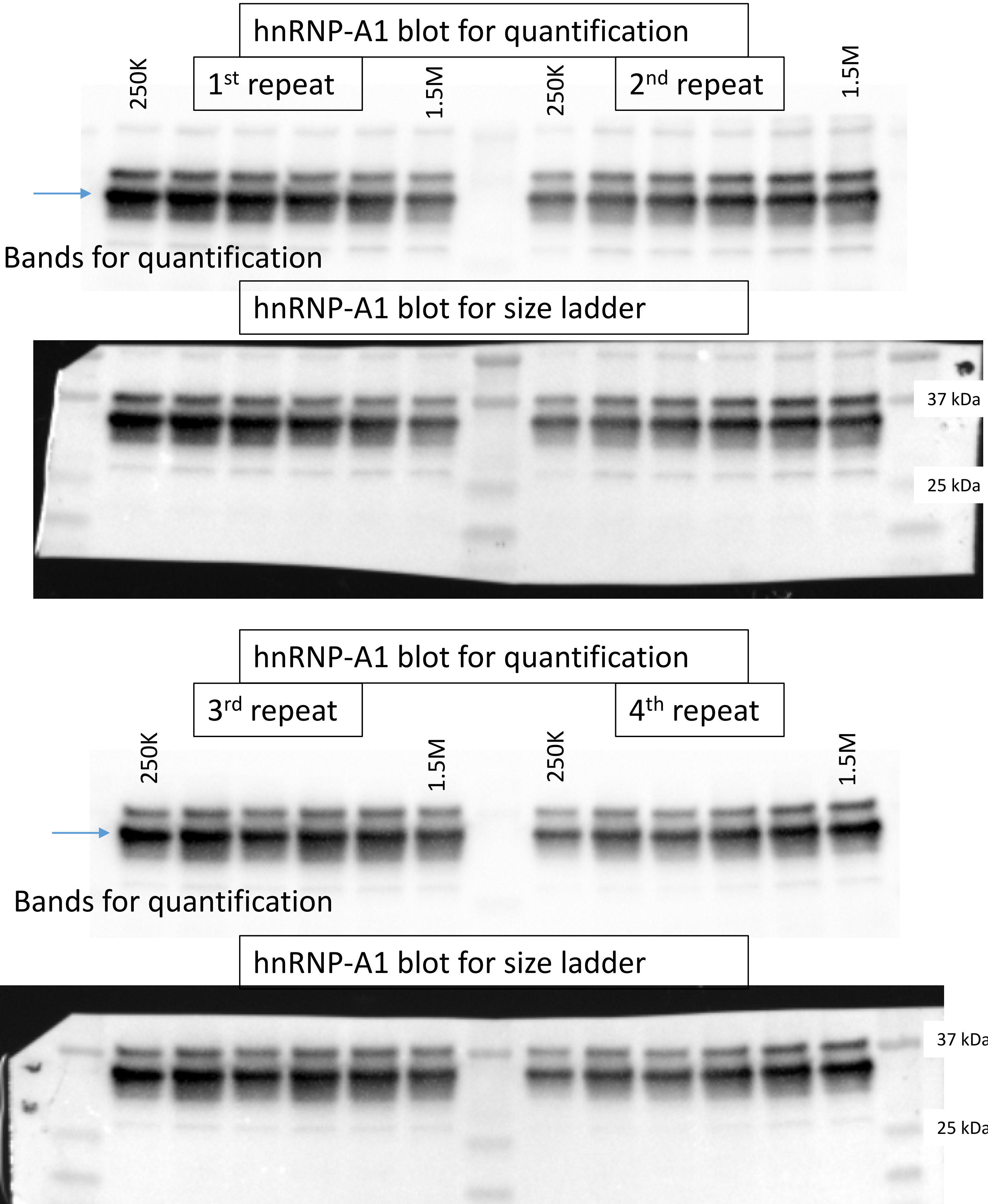

Figure 5 Immunoblot – splicing factors U2AF2

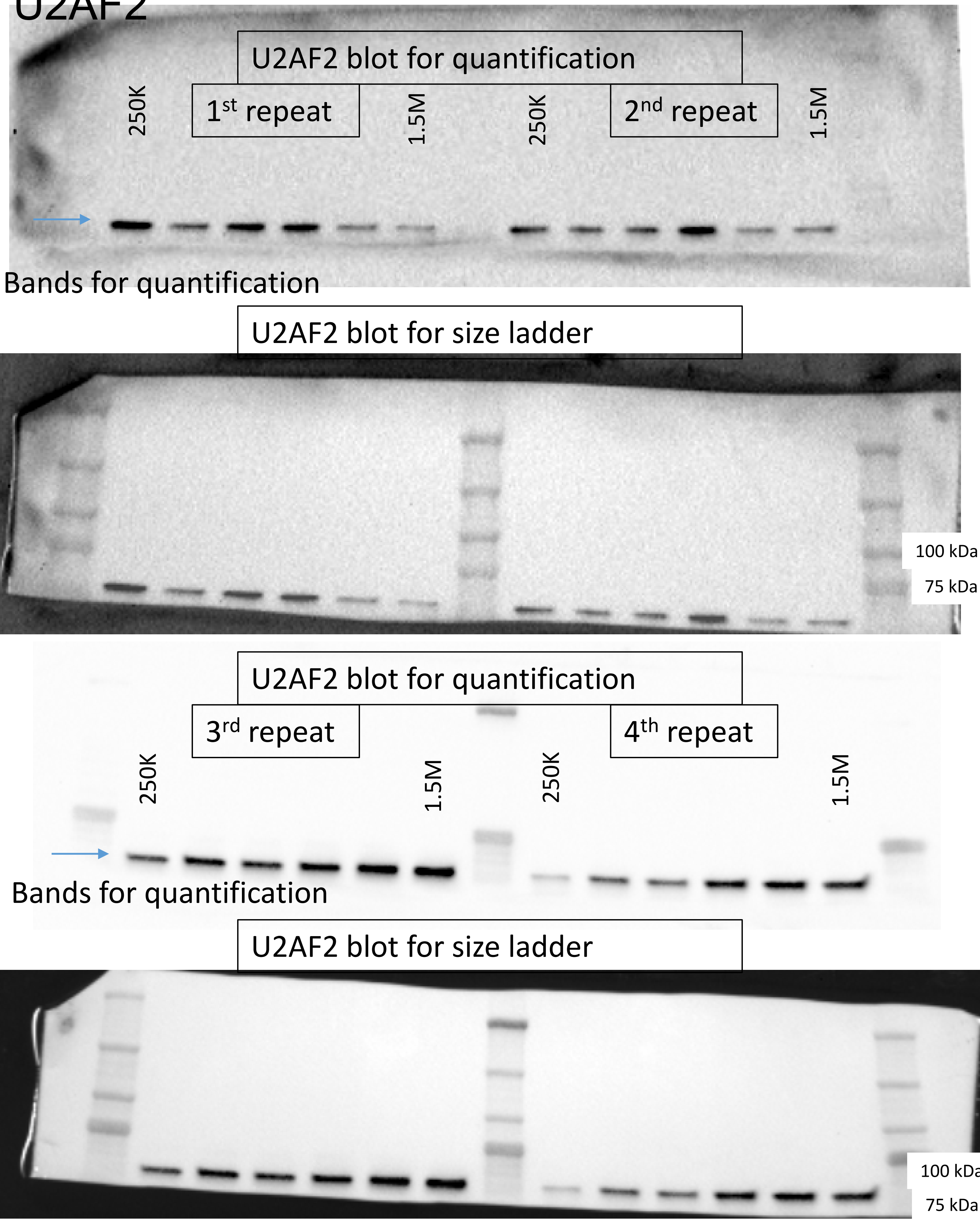

Figure 5 Immunoblot – splicing factors CDC40

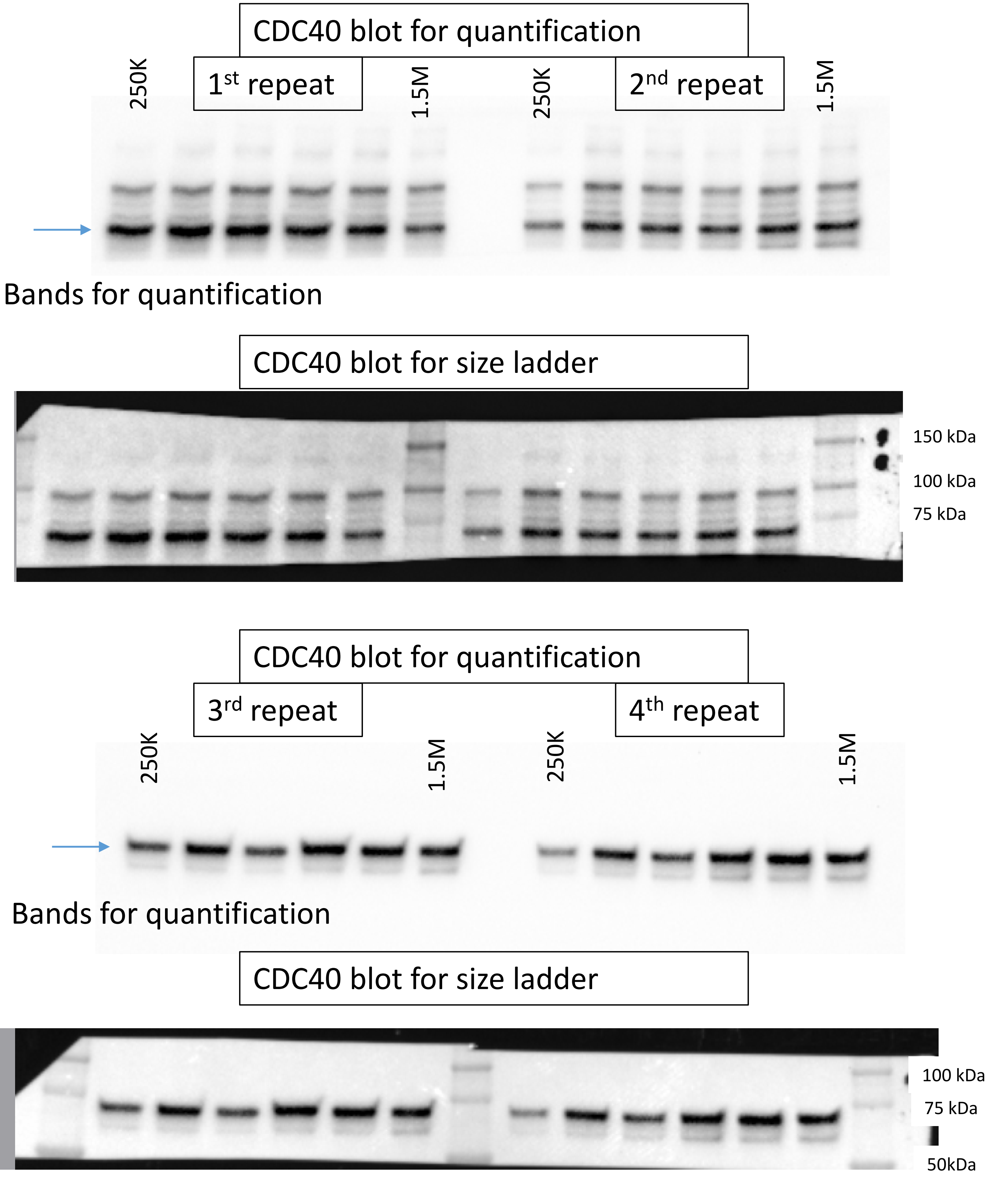

CDC40 top blot (1st and 2nd repeat) further explanation

Cut membrane was stained by SRPK1 antibody (rabbit polyclonal, Abcam, ab90527, 1:1000 in 5% BSA), stripped and stained by CDC40 antibody (rabbit monoclonal [EPR12539], Abcam, ab175924, 1:1000 in 5% BSA)

SRPK1 blot for size ladder

1st repeat

2nd repeat

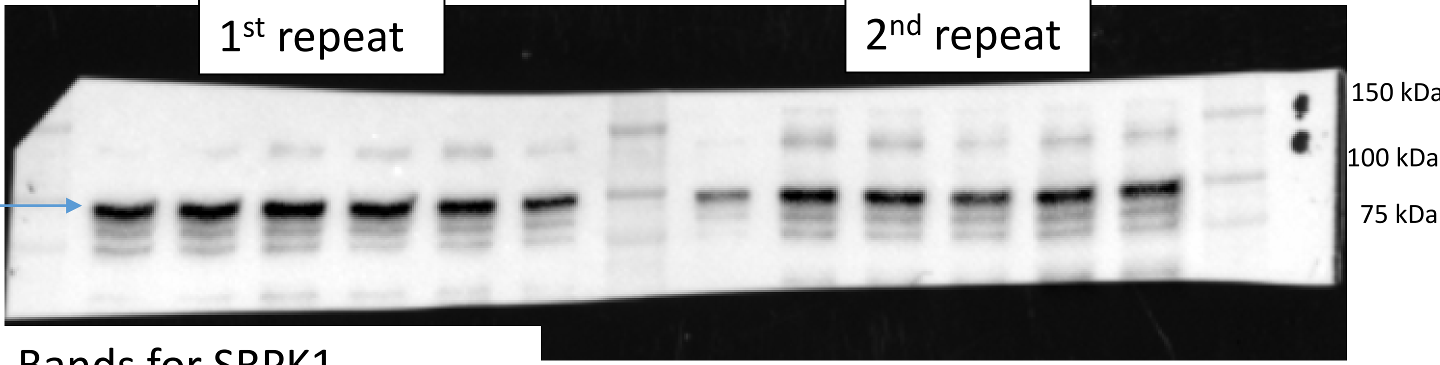

Bands for SRPK1 quantification

Stripping

CDC40 blot for size ladder

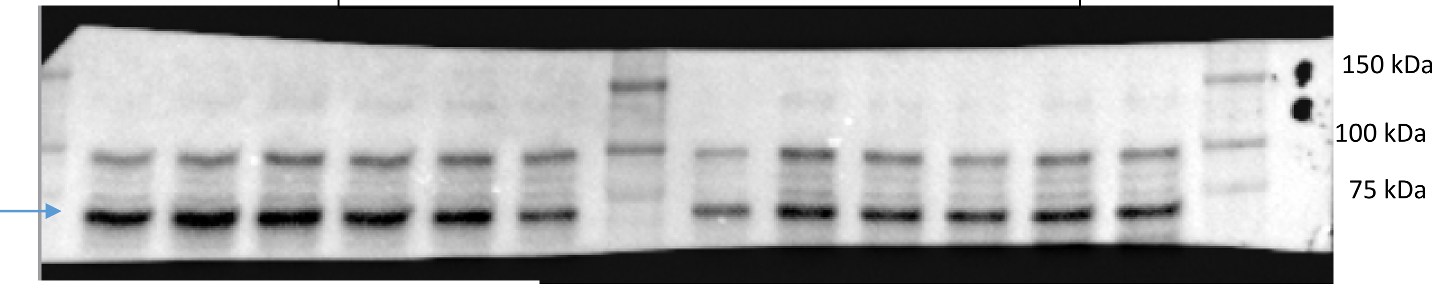

Bands for CDC40 quantification

Figure 5 Immunoblot – splicing factors
Beta-actin (loading control for 1st and 2nd repeat)

Beta-actin blot-1 for quantification

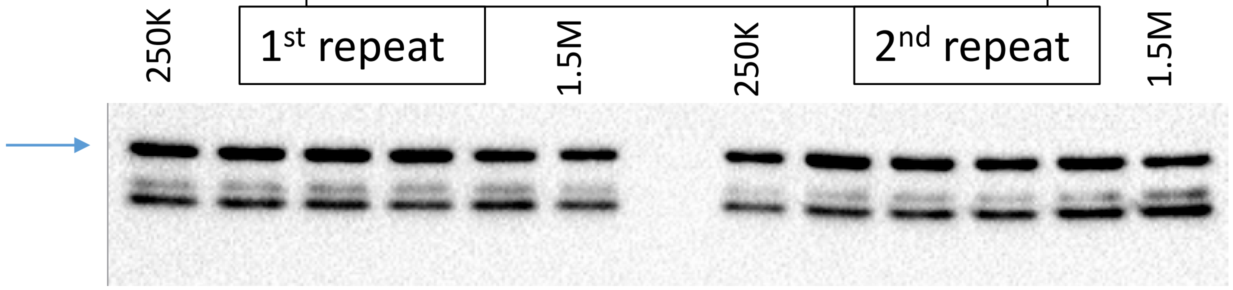

Upper bands for quantification
Lower bands are from HNRNP-A2B1 staining

Beta-actin blot-1 for size ladder

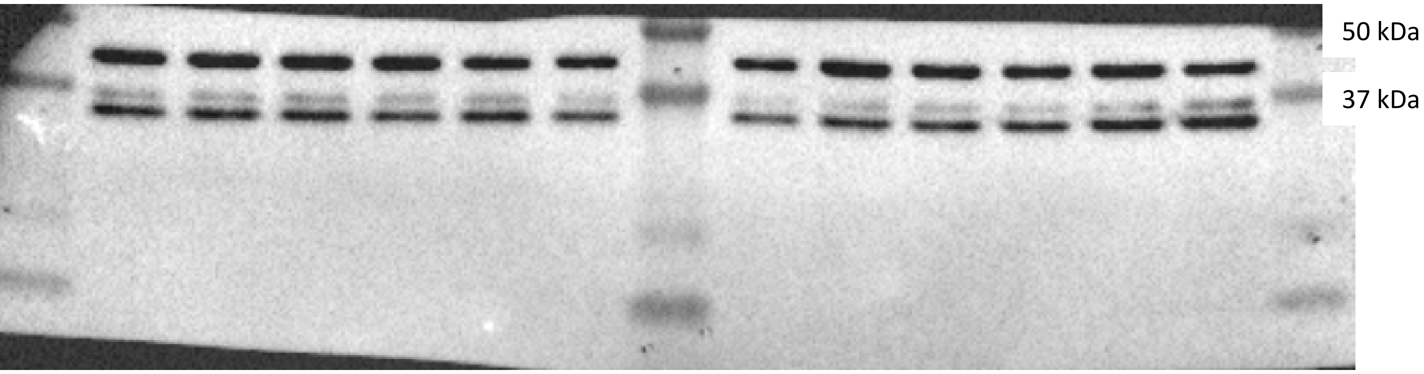

This beta-actin blot-1 was stained after stripping HNRNP-A2B1

HNRNP-A2B1 blot for size ladder

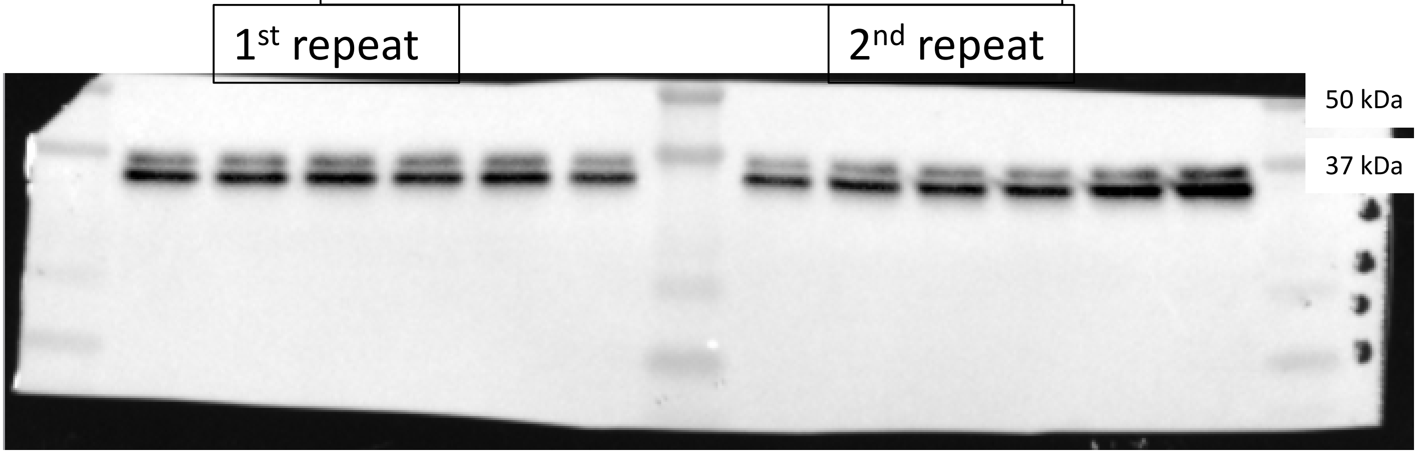

Figure 5 Immunoblot – splicing factors
Beta-actin (loading control for 1st and 2nd repeat)

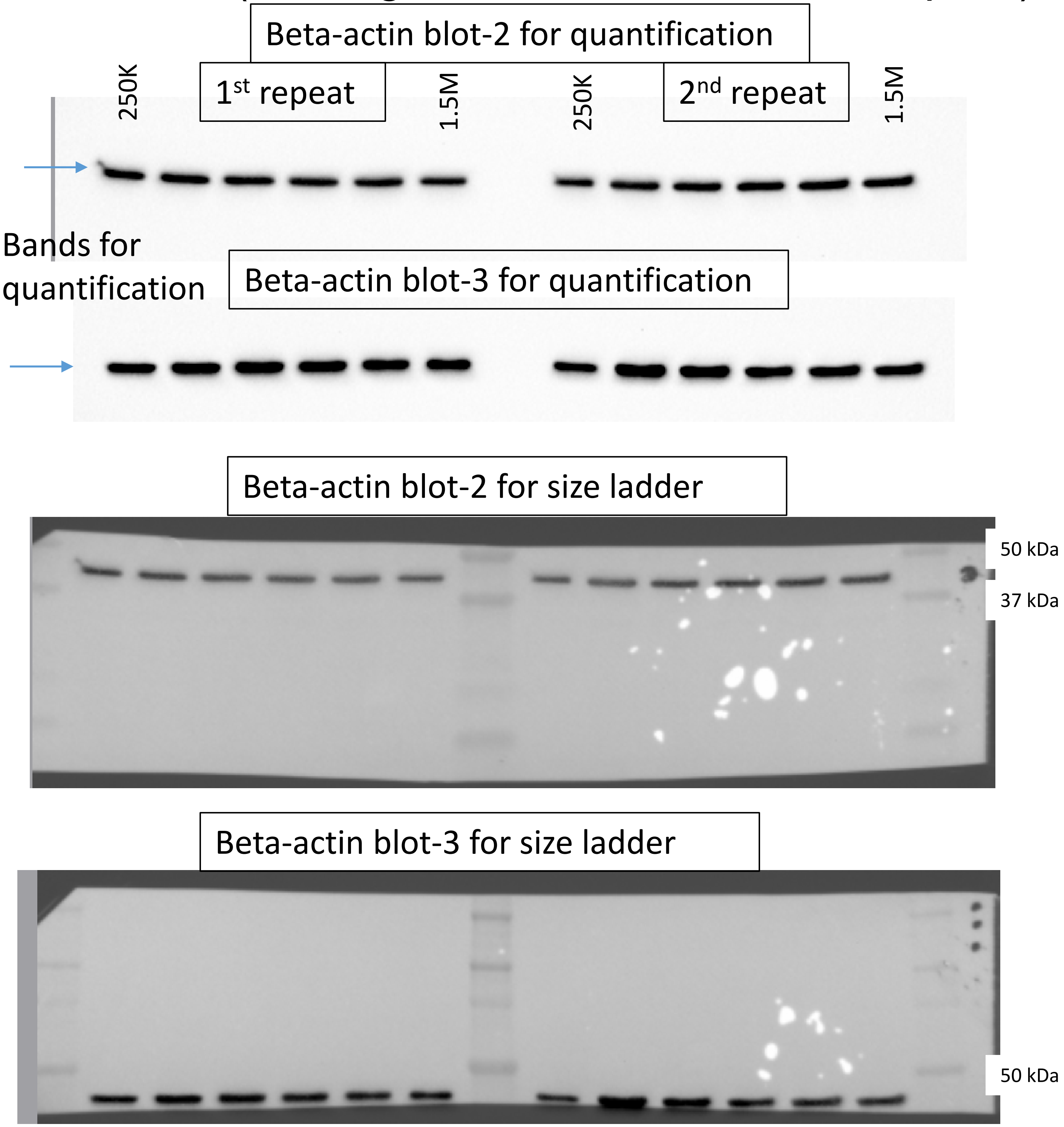

Figure 5 Immunoblot – splicing factors

Beta-actin and GAPDH (loading control for 1st and 2nd repeat)

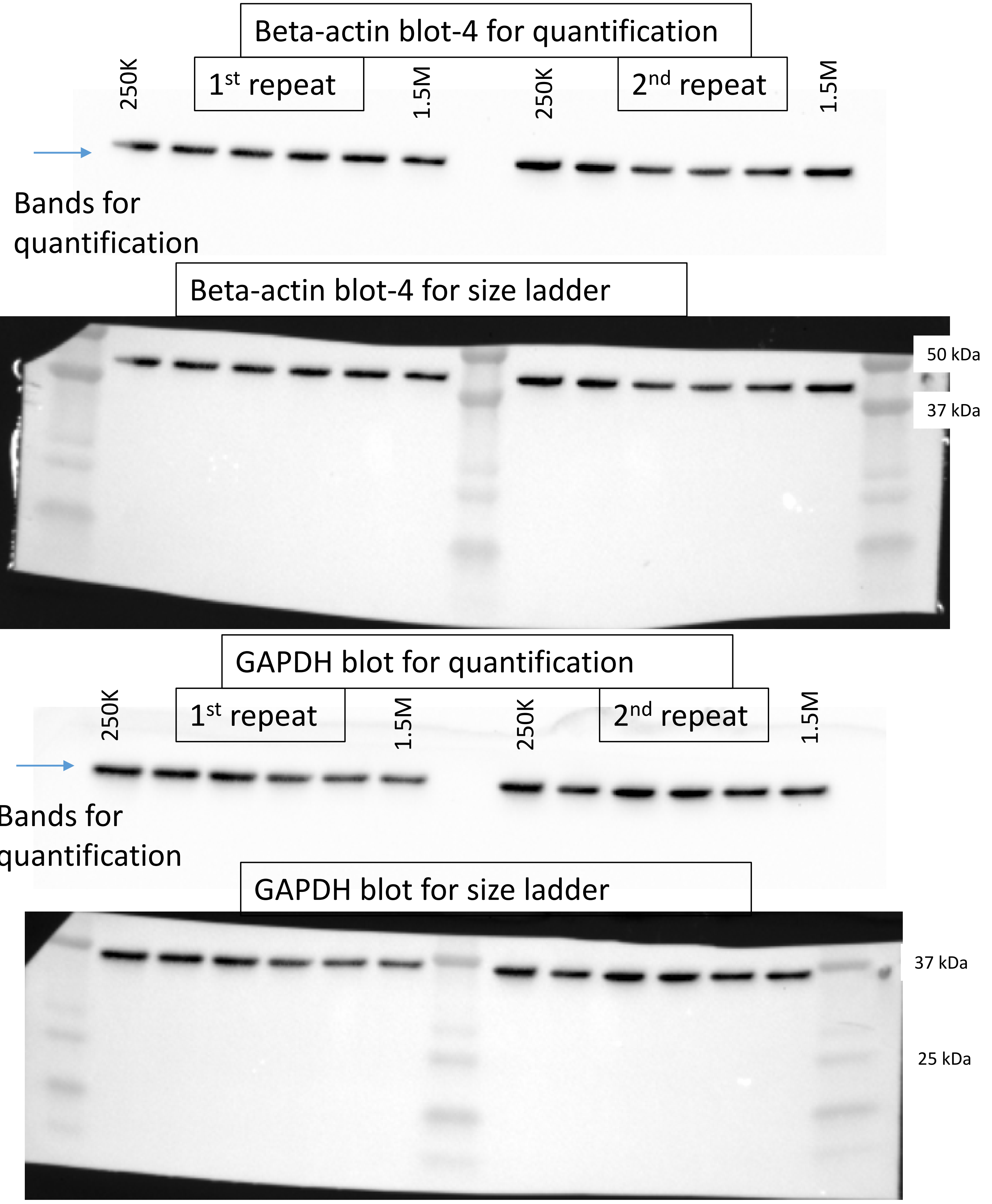

Figure 5 Immunoblot – splicing factors
Beta-actin (loading control for 1st and 2nd repeat)

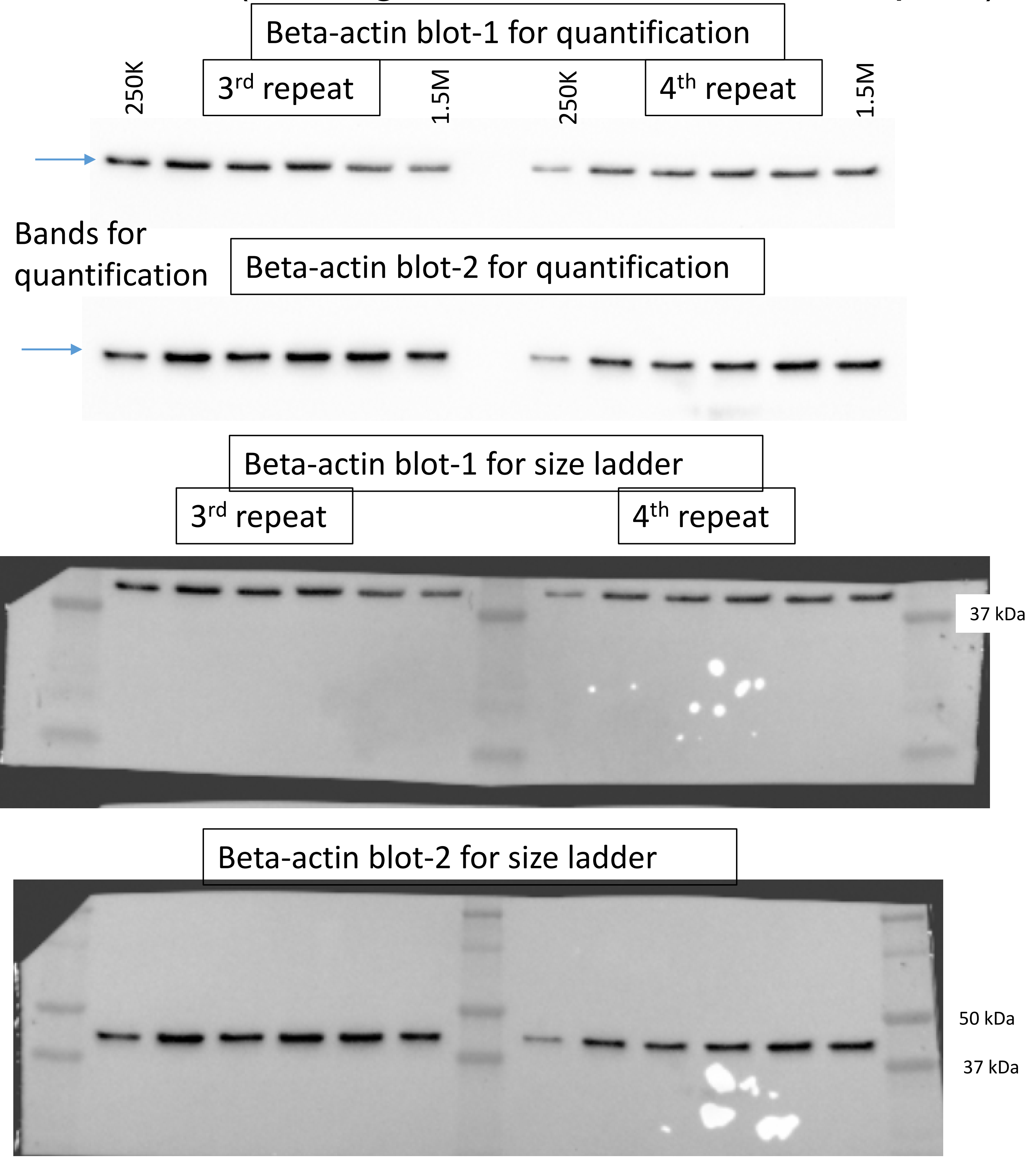

Figure 5 Immunoblot – splicing factors
Beta-actin (loading control for 3rd and 4th repeat)

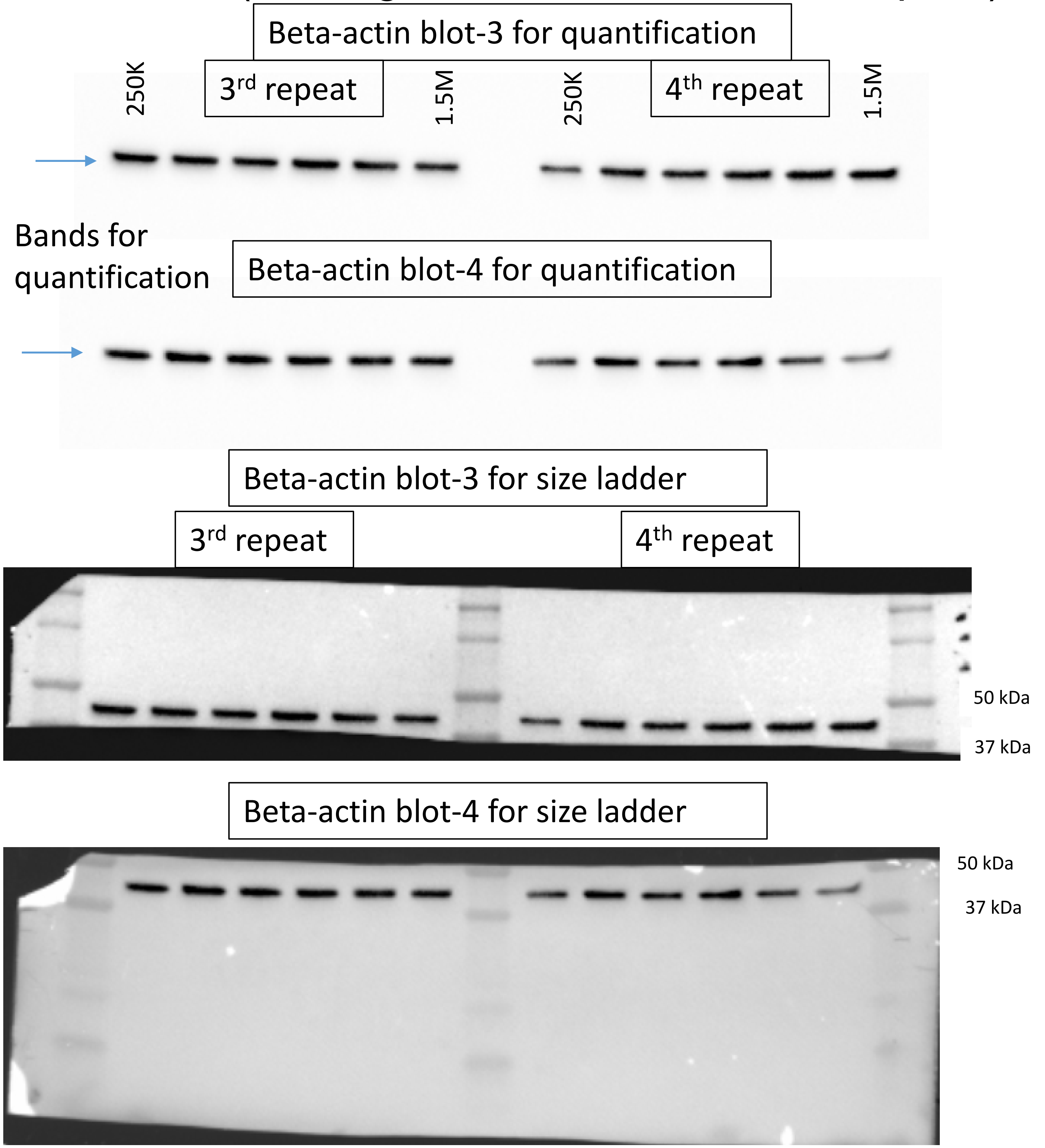

Figure 5 Immunoblot – splicing factors
Beta-actin (loading control for 3rd and 4th repeat)

Beta-actin blot-5 for quantification

250K | 1st repeat | 1.5M | 250K | 2nd repeat | 1.5M

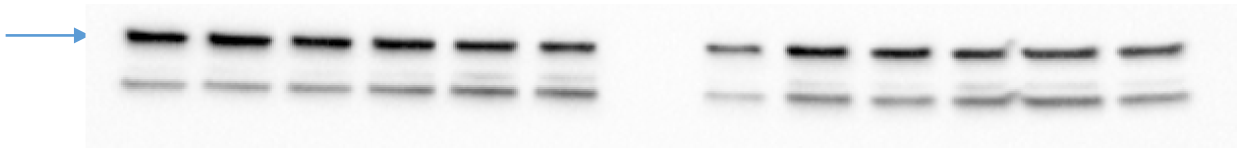

Upper bands for quantification

Lower bands are from HNRNP-A2B1 staining

Beta-actin blot-5 for size ladder

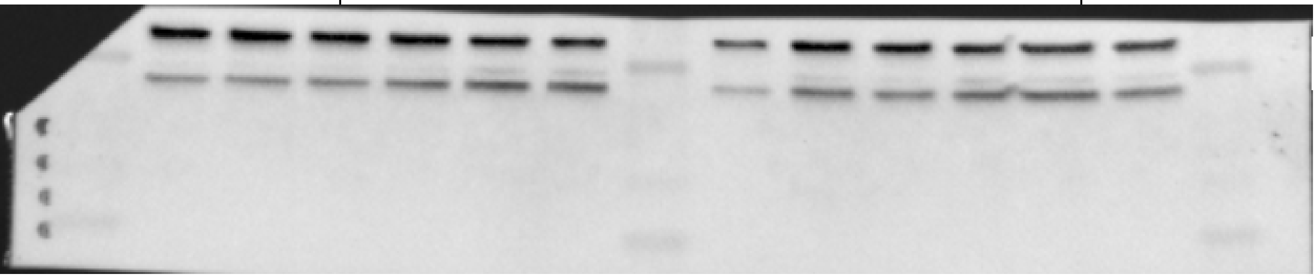

37 kDa

This beta-actin blot-5 was stained after stripping HNRNP-A2B1

HNRNP-A2B1 blot for size ladder

3rd repeat | 4th repeat

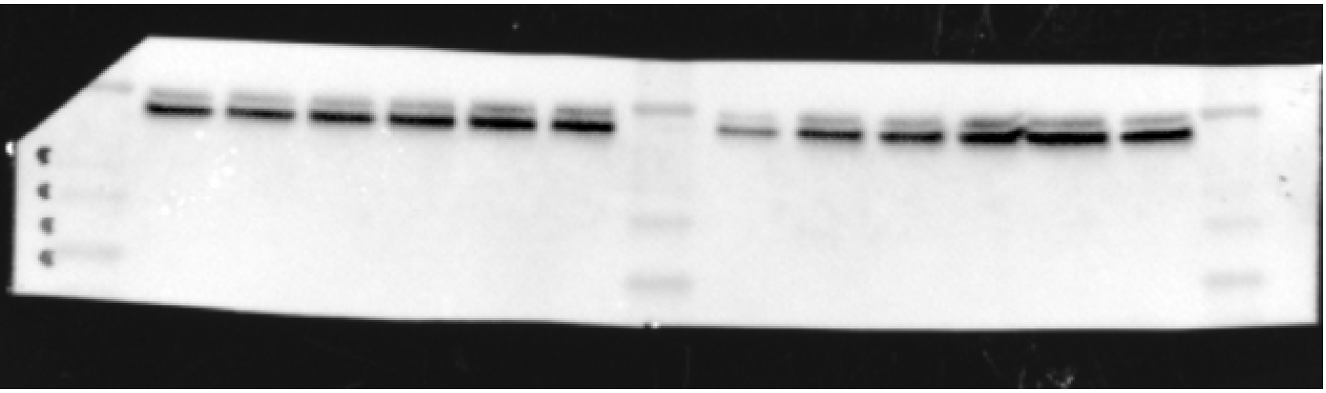

37 kDa

Figure 5 Immunoblot – splicing factors
Beta-actin (loading control for 3rd and 4th repeat)

Beta-actin blot-6 for quantification

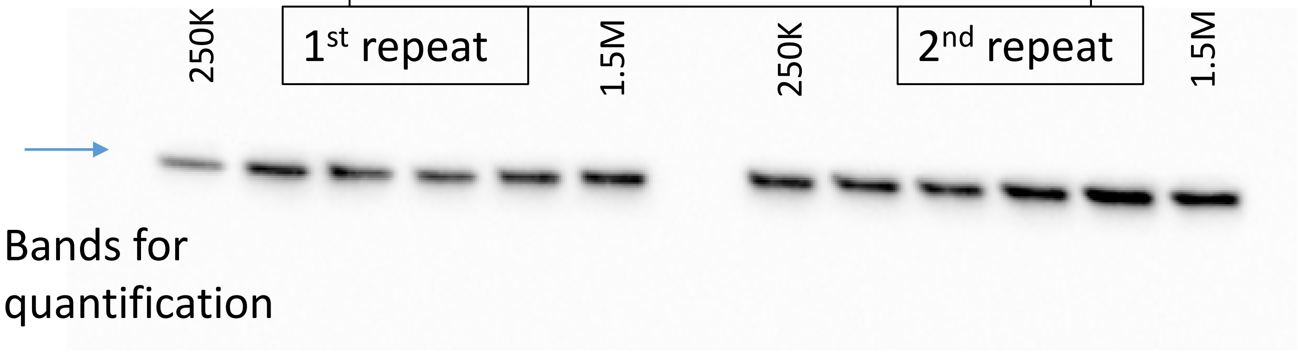

Beta-actin blot-6 for size ladder

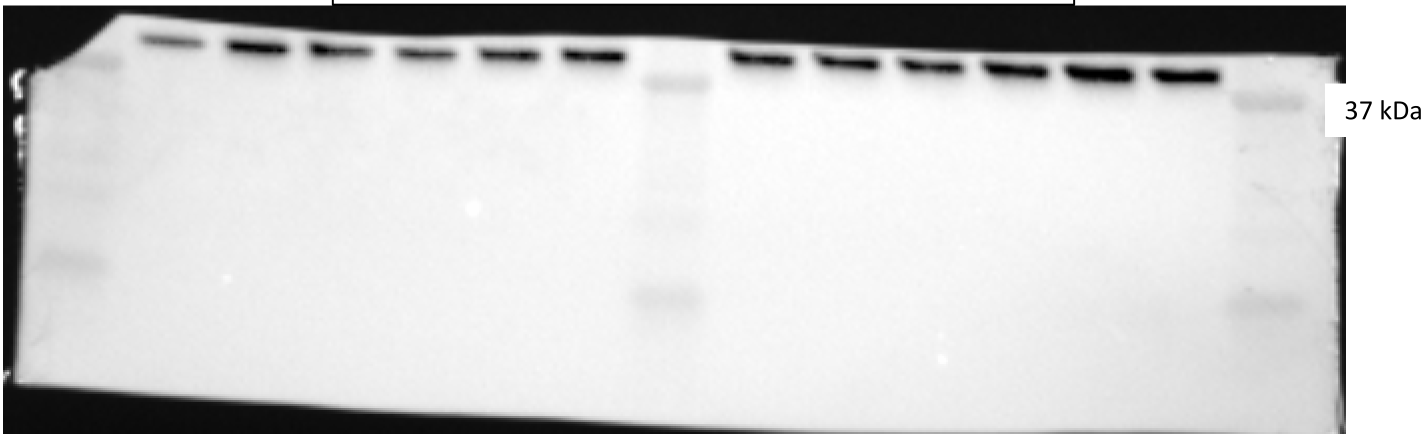

S4E Fig Immunoblot – PTBP1

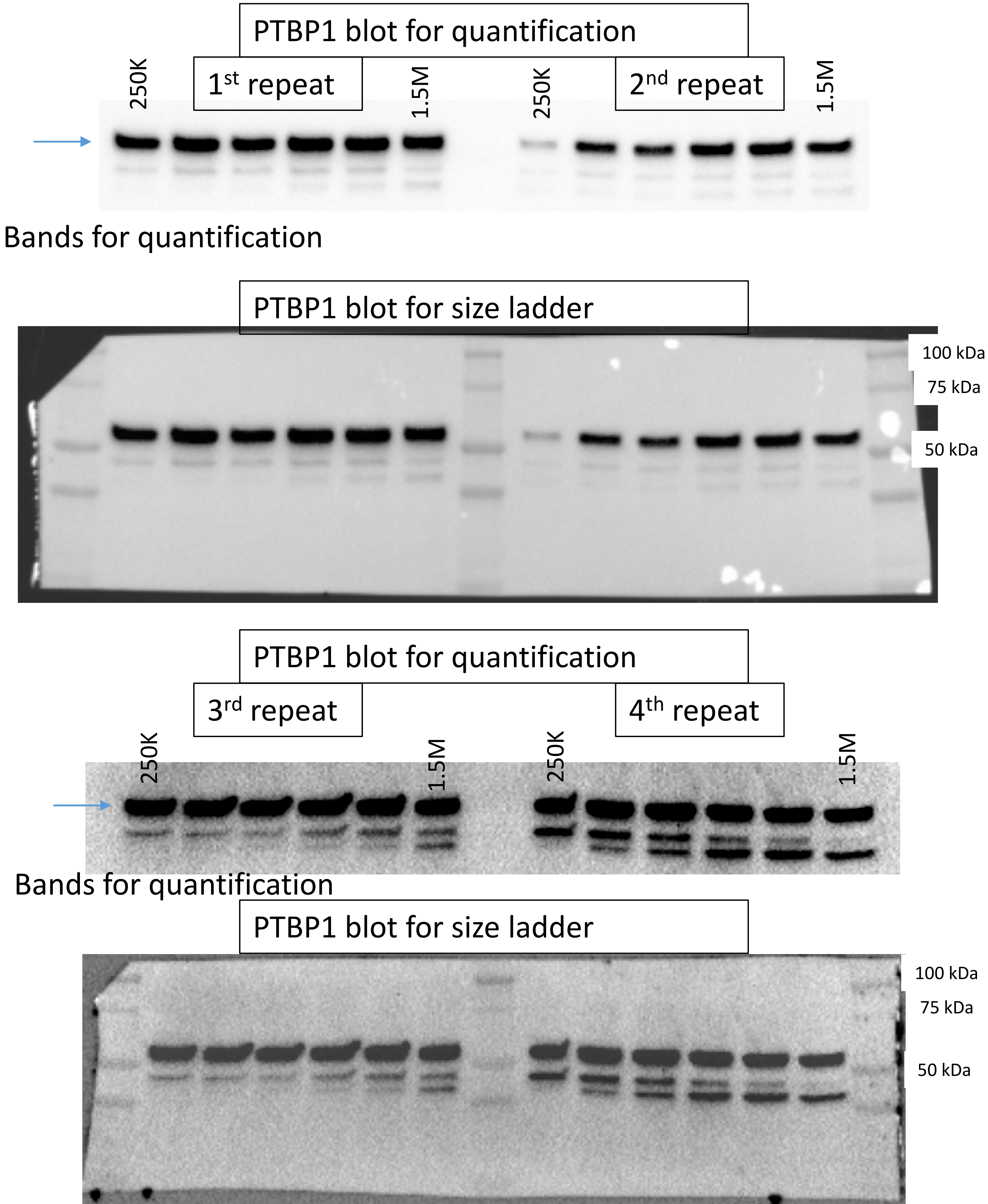

S4F Fig Immunoblot – NOVA1

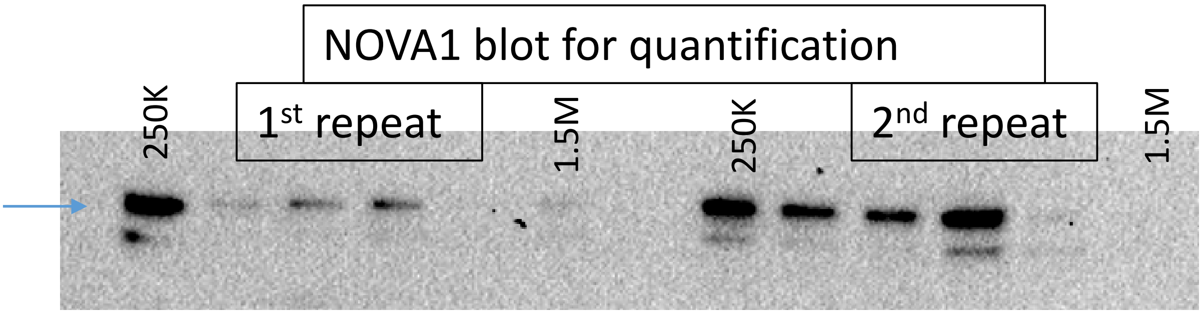

Bands for quantification

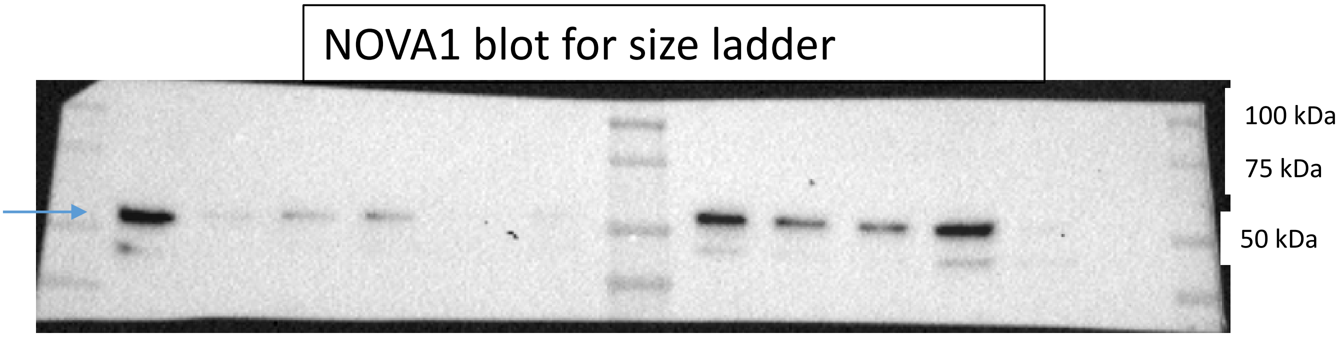

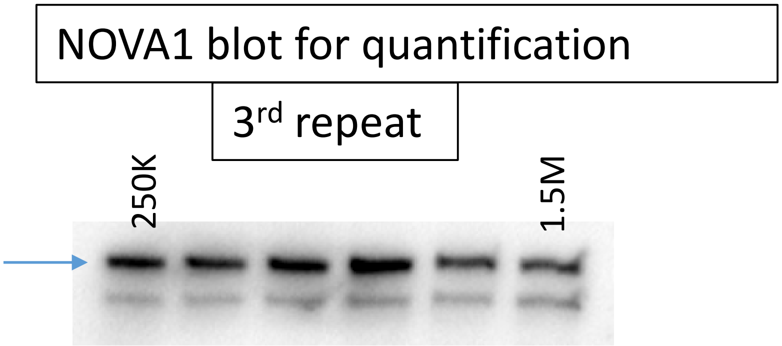

Bands for quantification

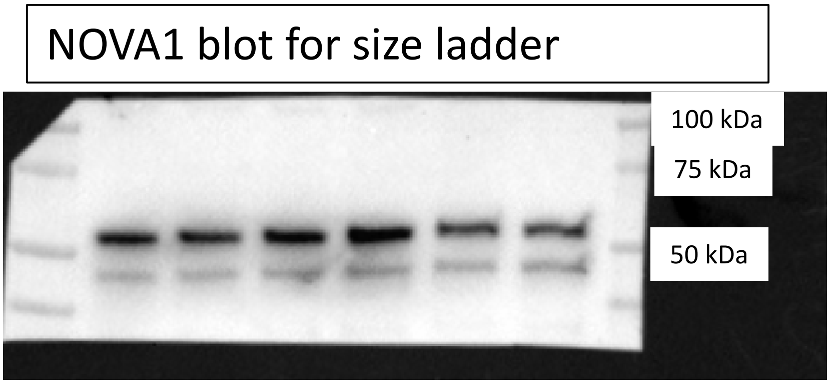

S4G Fig Immunoblot – PTBP2

PTBP2 blot for quantification

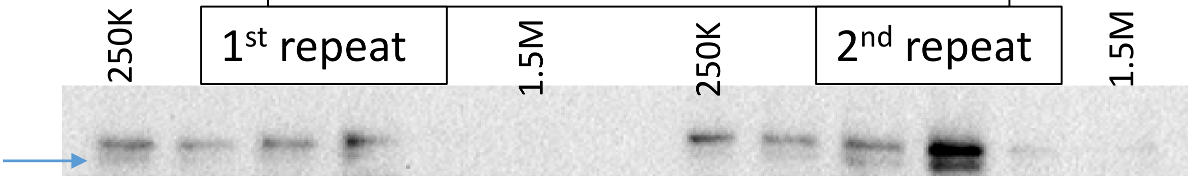

Bands for quantification
-> lower band because knockdown using siRNA targeting PTBP2 (Figure 1) resulted in the reduction of lower band

PTBP2 blot for size ladder

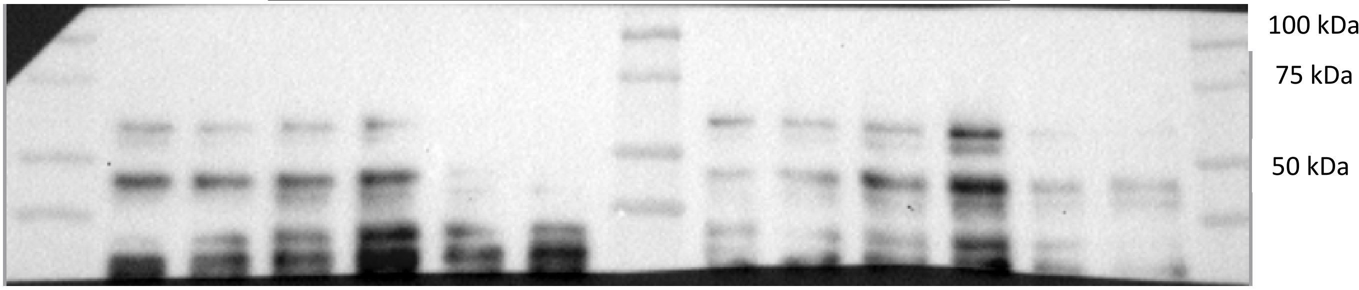

PTBP2 blot for quantification

3rd repeat

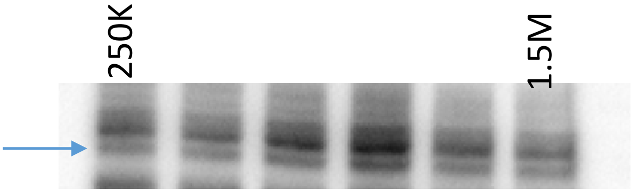

Lower Bands for quantification

PTBP2 blot for size ladder

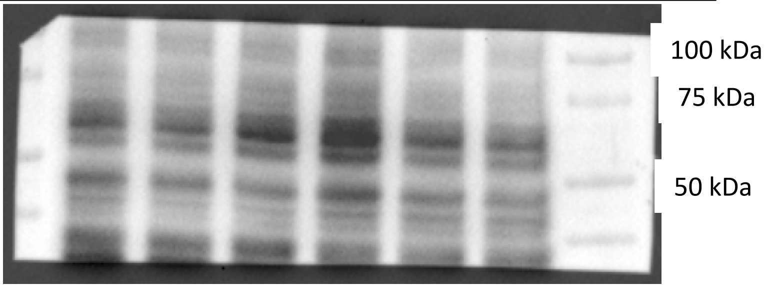

Figure 6 Immunoblot – siRNA treated Calu-6

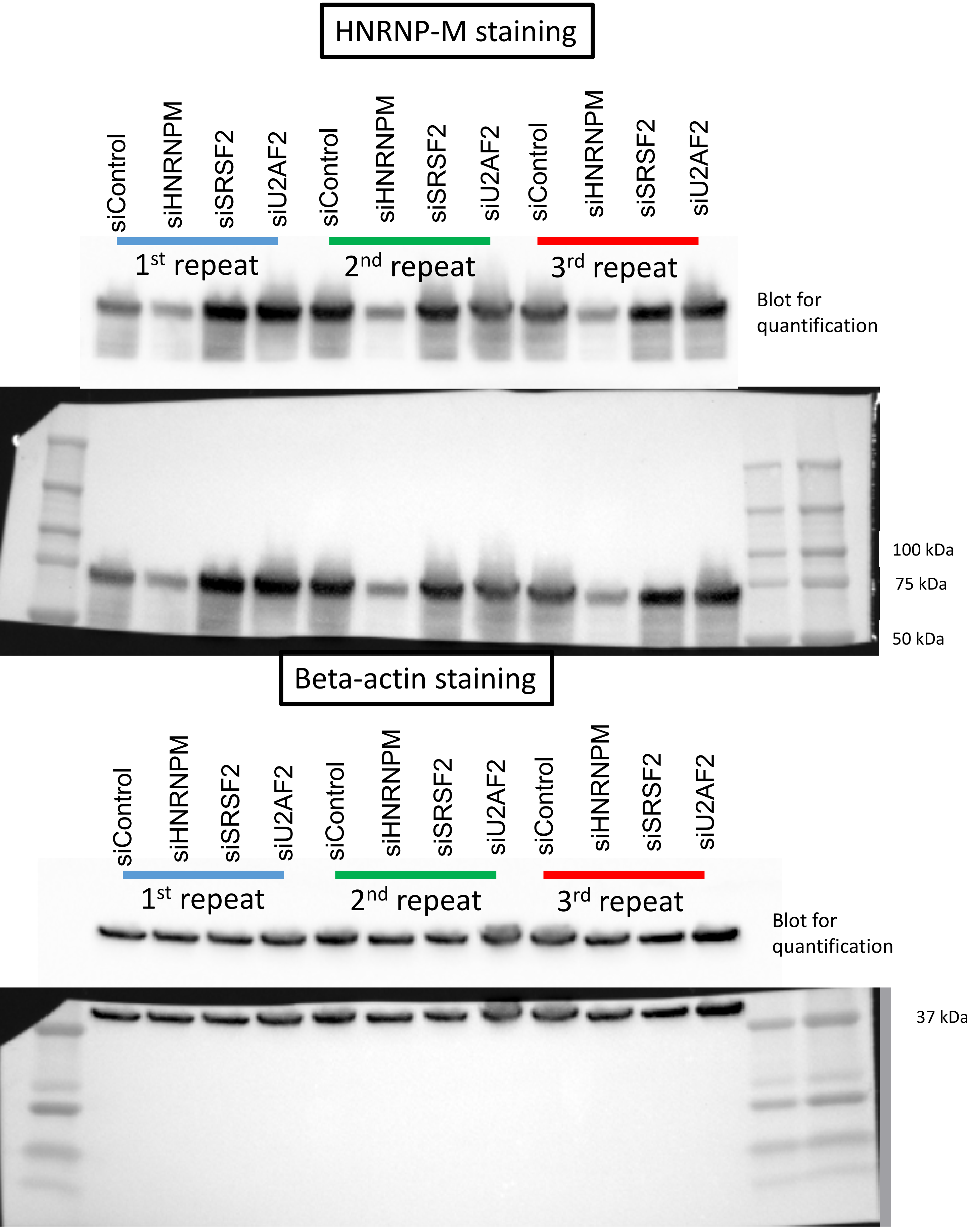

Figure 6 Immunoblot – siRNA treated Calu-6

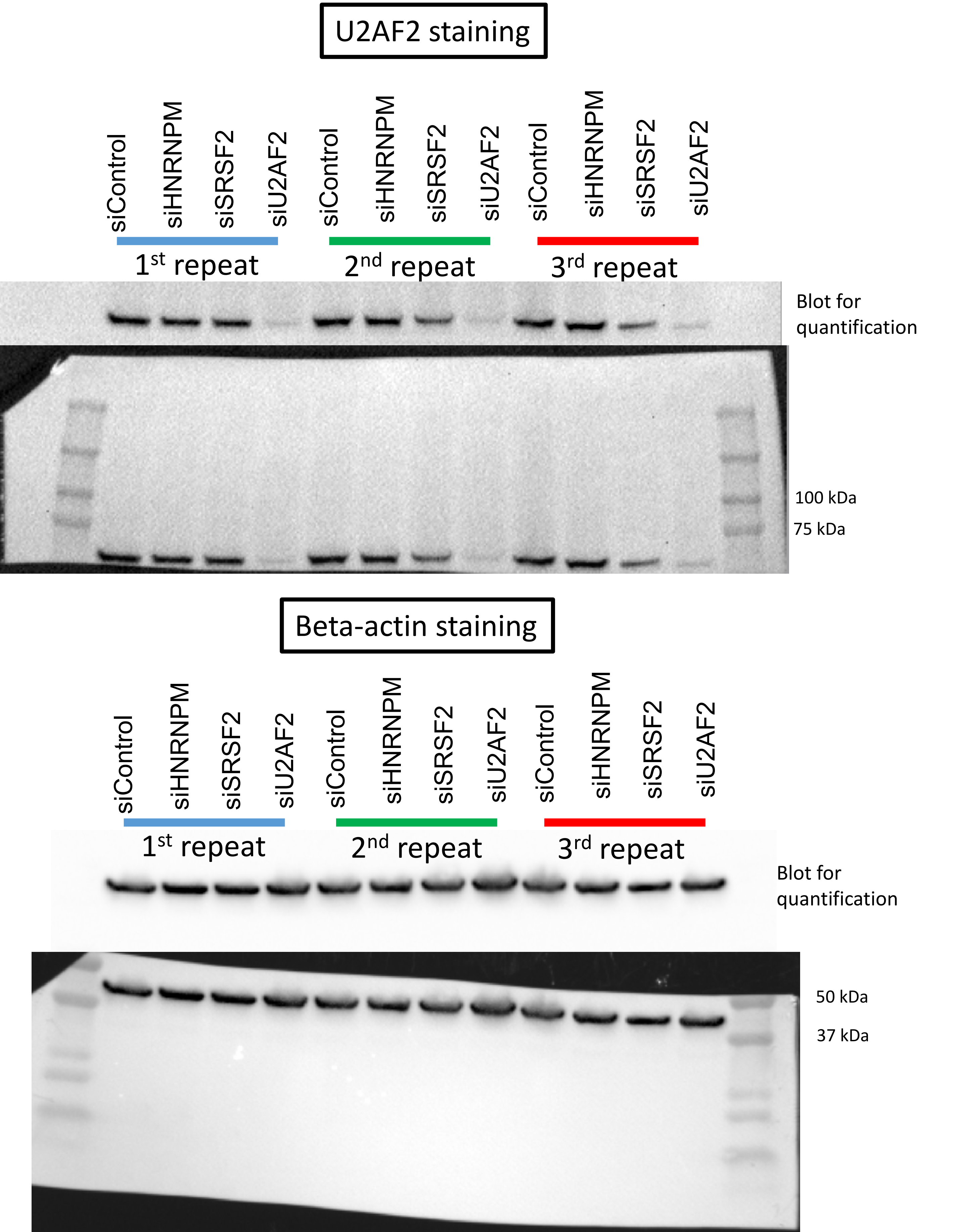

Figure 6 Immunoblot – siRNA treated Calu-6

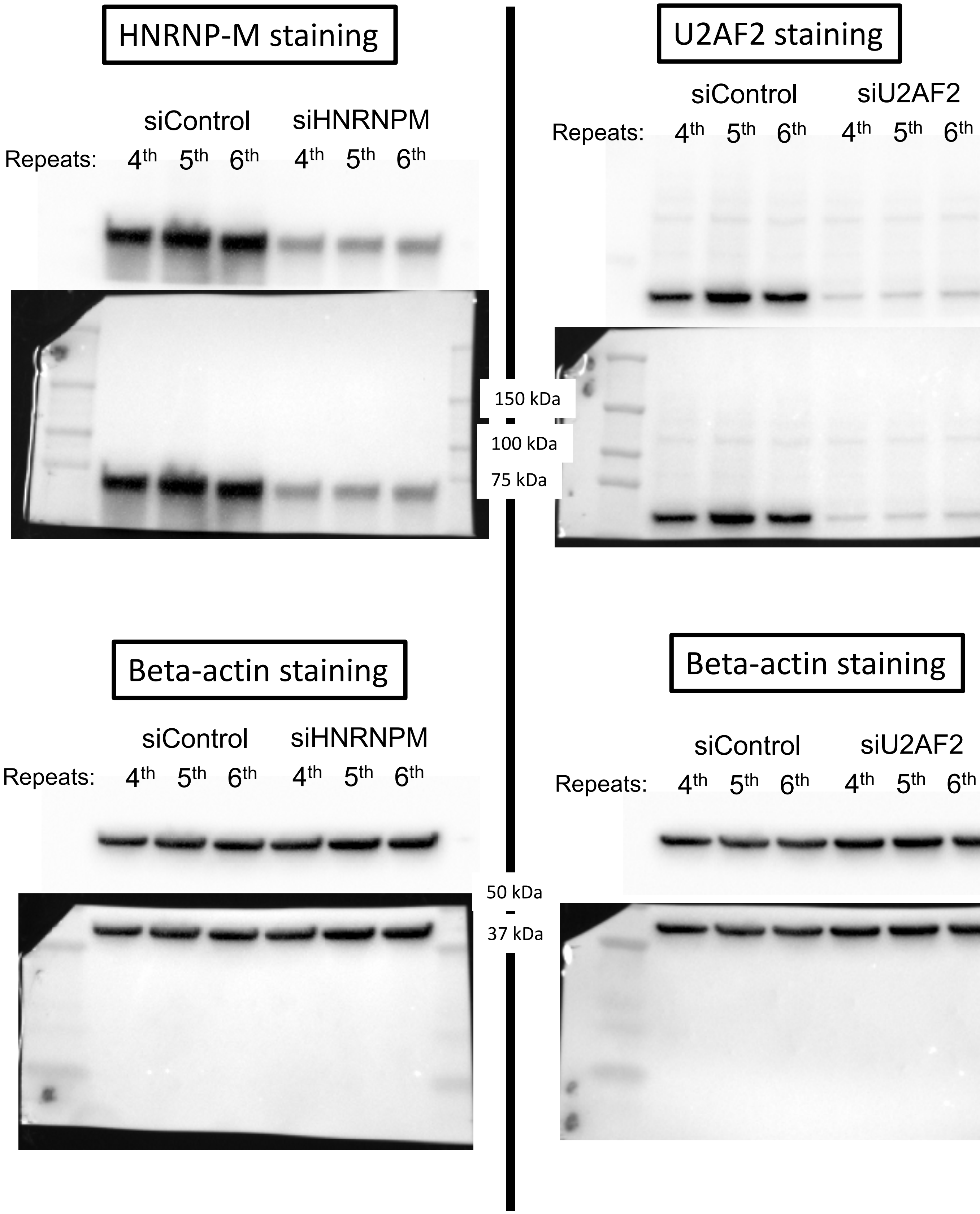

Figure 6 Immunoblot – siRNA treated Calu-6

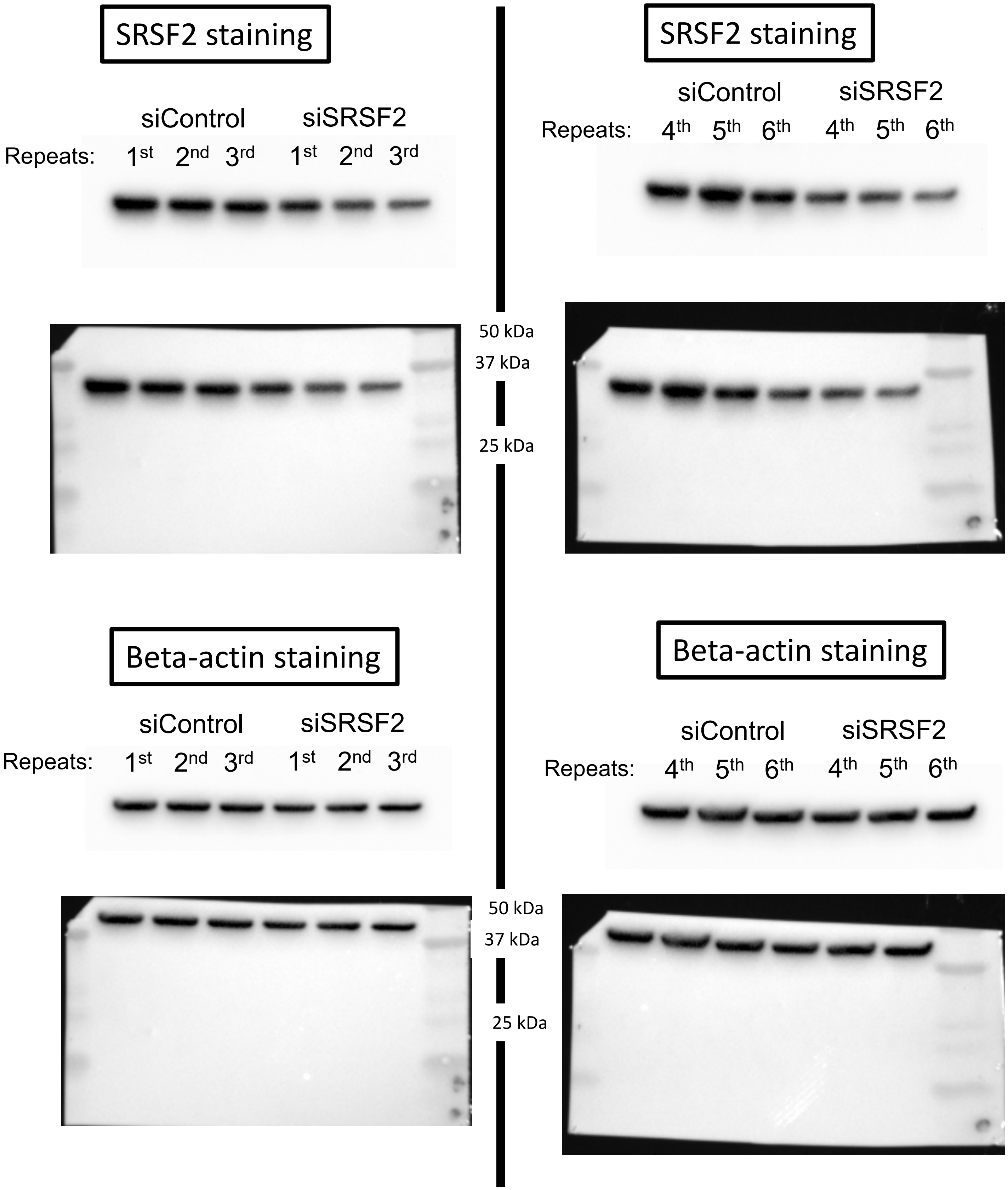

S5D and S5E Fig Immunoblot – iPSC vs NPC

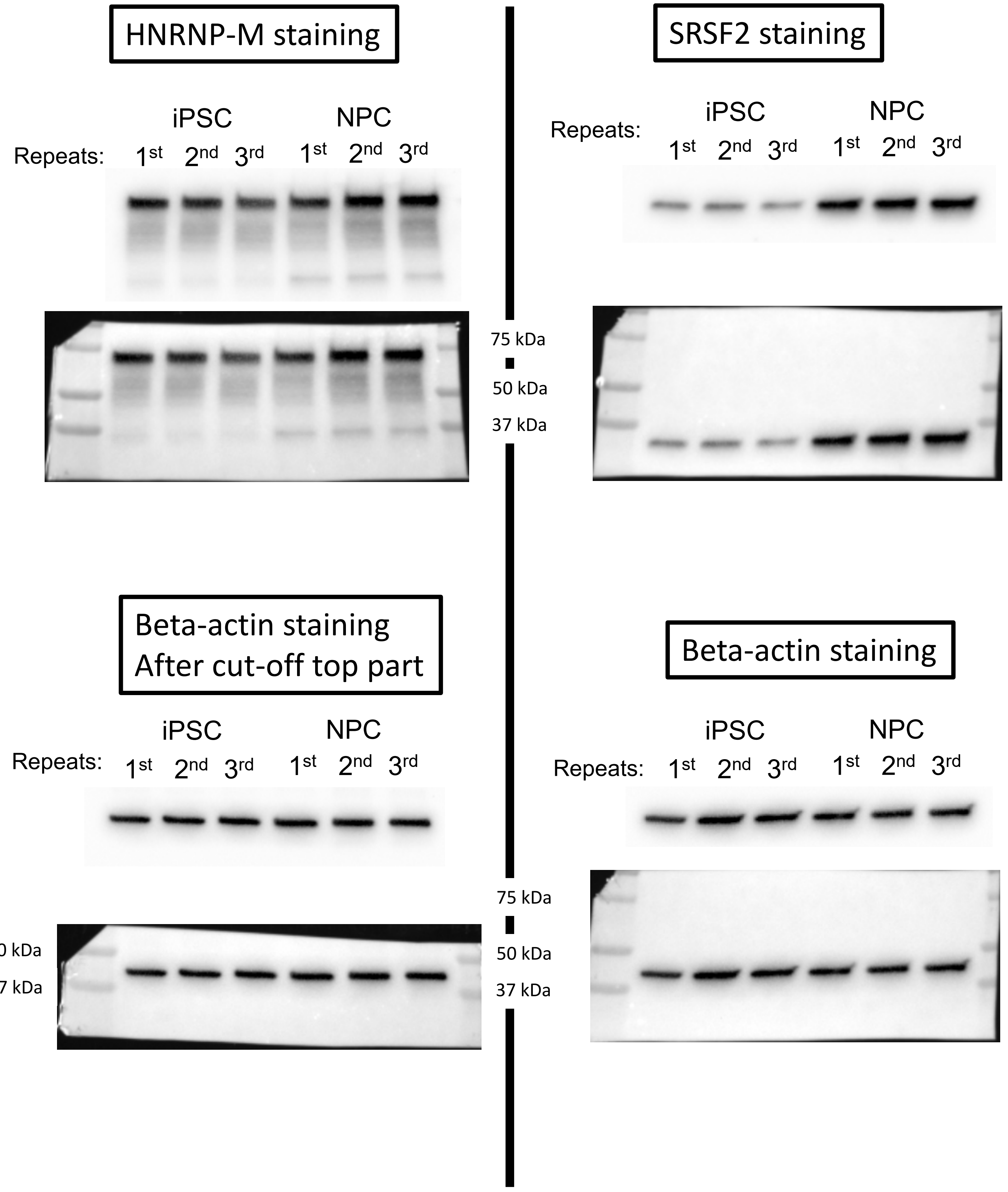

S5F Fig Immunoblot – iPSC vs NPC

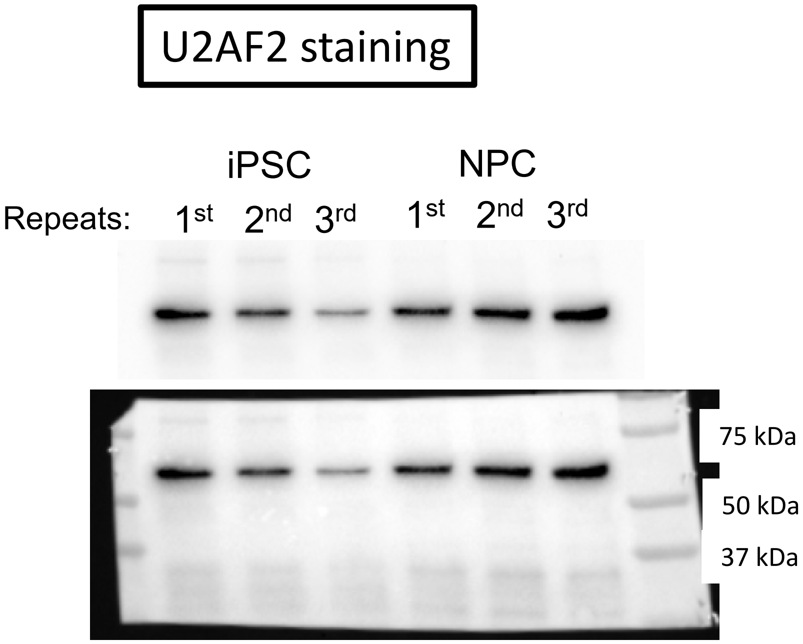

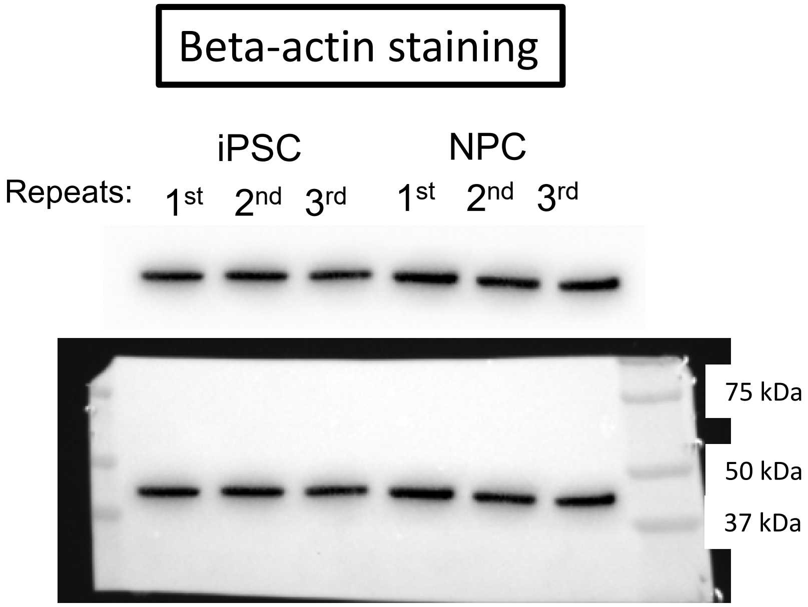

Supplement: S1 Raw images — (PDF) [file pone.0289327.s008.pdf]
